# Bladder Cancer Burden in the USA: Population Scenarios for 2040

**DOI:** 10.1016/j.euros.2025.11.013

**Published:** 2025-12-04

**Authors:** Hawre Jalal, Stella K. Kang, Fernando Alarid-Escudero, Stavroula A. Chrysanthopoulou, David Ulises Garibay-Trevino, Bruce L. Jacobs, Karen M. Kuntz, Praveen Kumar, Jonah H. Popp, Yuliia Sereda, Mutita Siriruchatanon, John B. Wong, Thomas A. Trikalinos, the CISNET Bladder Cancer Modeling Investigators

**Affiliations:** a School of Epidemiology and Public Health, University of Ottawa, Ottawa, ON, Canada; b Department of Radiology, Columbia University Irving Medical Center, New York, NY, USA; c Department of Health Policy, Stanford School of Medicine, Stanford, CA, USA; d Center for Health Policy, Freeman Spogli Institute, Stanford University, Stanford, CA, USA; e Department of Biostatistics and Center for Biostatistics and Health Data Science, School of Public Health, Brown University, Providence, RI, USA; f Division of Health Services Research, Department of Urology, University of Pittsburgh, Pittsburgh, PA, USA; g Division of Health Policy and Management, University of Minnesota School of Public Health, Minneapolis, MN, USA; h Department of Health Policy and Management, School of Public Health, University of Pittsburgh, Pittsburgh, PA, USA; i Public Health Dynamics Laboratory, School of Public Health, University of Pittsburgh, Pittsburgh, PA, USA; j Department of Health Services, Policy, and Practice and Center for Evidence Synthesis in Health, School of Public Health, Brown University, Providence, RI, USA; k Department of Medicine, Division of Clinical Decision Making, Tufts Medical Center, Tufts University School of Medicine, Boston, MA, USA

**Keywords:** Bladder cancer, Comparative modeling, Simulation, Epidemiological projections, Early detection

## Abstract

**Background and objective:**

Bladder cancer is the sixth most common cancer among men and is expensive to manage. We independently developed three microsimulation models that describe its natural history and explain epidemiological trends. We projected bladder cancer burden in the USA through 2040 to inform workforce planning.

**Methods:**

We calibrated the models to the Surveillance, Epidemiology and End Results (SEER) program incidence data and standardized key inputs. For White men, the highest-incidence subgroup, the models inferred unobservable epidemiological metrics, including lifetime risks by birth cohort and ages of the key events in the natural history. We simulated individual life histories under calibrated parameter sets and summarized the outcomes as yearly rates and counts.

**Key findings and limitations:**

Each model’s predictions reproduced SEER age- and stage-specific incidence data. Across models, the lifetime risk of bladder cancer grew from approximately 1.5–2.4% in the 1910 to 3.1–4.4% in the 2010 birth cohorts, consistent with longevity and smoking exposure patterns. Of the cancer cases, 75% instantiate after ages 61–64 yr. The median model durations from when a cancer is screen detectable to its clinical manifestation were 2.1–3.3 yr, with a wide range across individuals. Through 2040, the incidence standardized to the 2000 US population declined by 0.4–0.6%/yr (consistent with the declining smoking rates, the most important environmental risk factor), but the annual incidence and new cases increased by 1.5–1.8%/yr (because the baby boomer population is living longer). Modeling supplements incomplete data with assumptions, but similar findings across independent models suggest some robustness to assumptions.

**Conclusions and clinical implications:**

Projected cohort longevity and smoking patterns imply an increased disease burden in the future, which may benefit from commensurate increased research and resources. From the inferred natural history, we speculate a theoretical opportunity for screening, which should be investigated with dedicated modeling and empirical studies.

**Patient summary:**

Three computer simulation models predicted the future incidence of bladder cancer burden in White men, in whom this cancer is most common. The models found that although the future incidence of bladder cancer would decrease slightly over time (consistent with the declining smoking rates, the most important environmental risk factor), the overall disease burden increased because the baby boomer population is living longer.

## Take Home Message

Mathematical modeling projected slight decreases in the age-adjusted incidence of bladder cancer, ostensibly tracking historical smoking and longevity patterns, but increases in overall disease burden as the population ages and expands. Projected urology workforce shortages accentuate the trends’ adverse societal impact.

## 1 Introduction

Bladder cancer is the sixth most common cancer in the USA, with an estimated 83 190 cases in 2024, and the eighth leading cause of cancer mortality in men [1], [2]. Despite reductions in exposure to well-recognized risk factors, such as smoking and environmental carcinogens [3], [4], bladder cancer incidence and mortality have remained largely unchanged through the mid-2000s, with a 1–1.5% annual decline in age-standardized incidence since 2005 [5]. Although approximately 75% of bladder cancer cases are detected in non–muscle-invasive stages [6], their annual cost is a staggering 6.5 billion dollars in the USA alone due to the high rates of recurrence and repeated surveillance and therapy when disease is high risk [7].

Understanding the natural history of bladder cancer is useful for describing epidemiological trends and informing policy discussions, for example, by gauging possible prevention and control opportunities and projecting future disease burden. However, most of the key events in the natural history are not directly observed in empirical studies and routinely collected data. For example, the ages at which tumors first emerge, and how soon afterward these become screen detectable, cannot be observed directly, but determine whether early detection efforts are practical in the general population or high-risk subgroups [8]. Similarly, the ages at which cancer emerges and progresses in people with different exposures to carcinogens [9], men versus women [10], and White versus Black populations [11] are key for assessing the impact of carcinogen control policies and explaining variations in outcomes across sexes and racial groups.

Little empirical data exist that directly inform the timing of the key events, such as the emergence or progression of tumors, in the natural history of bladder cancer. However, empirical data on incidence and postdiagnosis surveillance provide indirect information about these unobserved events that can be explored using mathematical simulation modeling at the population level. Because mathematical models include extra-evidentiary information by making clinically plausible assumptions, it is the best practice to examine several independently developed models that are calibrated on the same data but make different assumptions [12]. This strategy of *comparative modeling* as a means of conferring some robustness over modeling assumptions has been utilized extensively by the Cancer Incidence and Surveillance Modeling Network (CISNET), a National Cancer Institute (NCI)-funded consortium that uses population-based modeling to inform cancer control efforts. Here, we introduce the CISNET Bladder Cancer site, which comprises three independently developed population models calibrated to the US population [13], [14].

The objective of this work is to characterize the natural history of bladder cancer. This information is valuable to clinical researchers who design new empirical studies. It also has practical relevance for public policy for early cancer control and workforce planning. To demonstrate the latter, we make a foray on two questions focusing on White men, the population subgroup with the highest incidence: First, what is the projected population burden of bladder cancer through 2040? Second, based on when bladder cancer initially appears and progresses, is screening for bladder cancer theoretically feasible? While the detailed treatment of either policy area requires dedicated mathematical modeling and new empirical studies, we can still get nontrivial insights.

## 2 Patients and methods

The CISNET Bladder Cancer site comprises three independently developed population models: Cancer of the Bladder R-based Analytic Simulator (COBRAS), Kystis, and the Simulation of Cancers of the Urinary Tract (SCOUT). The model profiles detailed in the Supplementary material specify each model’s mathematical structure, assumptions, data sources, simulation algorithm, and calibration. We provide succinct summaries below.

### 2.1 Model descriptions

COBRAS, Kystis, and SCOUT are microsimulation models of the incidence, natural history, surveillance, treatment, and mortality of bladder cancer, developed with feedback from clinical experts (Table 1). COBRAS and Kystis simulate events in continuous time [15], [16] and SCOUT simulates events in discrete time, in monthly cycles. All models generate hypothetical individuals born in specific years (1900 onward) or, by stacking birth cohorts, approximate the US population simulating births and deaths but not accounting for migration. The models average outcomes over simulated individuals to predict population-level outcomes of bladder cancer.

**Table 1:** Comparison of the three mathematical models of the CISNET Bladder Cancer site

Property	COBRAS	Kystis	SCOUT
*Model characteristics*			
Model type	Continuous time DES	Continuous time DES	Discrete time state transition
Population model	Yes	Yes	Yes
Model individuals (microsimulation)	Yes	Yes	Yes
Model of organ	No	Yes	No
*Lesion risk*			
Mechanism	Random-intercept zero-inflated Poisson model	Nonhomogeneous Poisson point process	Nonhomogeneous Markov model
Demographic risk factors	Age, sex, race, birth year	Age, sex, race, birth year	Age, sex, race, birth year
Modifiable risk factors			
Smoking	Yes	Yes	Yes
Occupational/environmental toxins	Yes	Yes	No
Genetic risk factors	No	No	No
Comorbidities	No	No	CKD stage, diabetes mellitus, hypertension
Varies randomly across individuals	Yes	Yes	Yes
Multiple lesions possible	Yes	Yes	Yes
*Lesion attributes*			
Transitional cell carcinoma	PUNLMP, Ta, T1–4, Tis	Lesions of low invasive potential (Ta, T1, T2+, Tis)	Ta, T1–4, Tis
Grade	Low/high (Ta only)	Low/high (Ta and T1)	Low/high (Ta only)
Nontransitional cell carcinoma	Yes	Yes	No
Morphology/configuration	Not explicitly modeled	Flat, sessile, pedunculated	No
Size	Lesion diameter and volume	Cell number and surface area on bladder	No
Location within bladder	No	Mapped to 678 unit areas/tiles on a sphere	No
*Lesion growth model*			
Mechanism	Gompertzian	Generalized logistic (Verhulst)	Not applicable
Size modeled as continuous	Yes	Yes	Not applicable
Varies randomly across individuals	Yes	Yes	Not applicable
Varies randomly across lesions	Yes	Yes	Not applicable
*Transitions among lesions*			
Initial lesion	Ta-LG, Ta-HG, Tis	Ta-LG, Ta-HG, Tis, T1-HG (for nonurothelial neoplasms)	Ta-LG, Ta-HG/Tis
Allowable transitions	Ta-LG → Ta-HG → T1 → T2 → T3 → metastasis	For both LG and HG lesions, Ta → T1 → T2/T3 → metastasis, and similar for Tis	Ta-LG → Ta-HG/Tis → T1 → T2 → T3 → nonmetastasis T4 → metastasis
Mechanism	Stochastic function of lesion size	Stochastic function of lesion size	Age-dependent stochastic matrix
Varies systematically by	Lesion stage and grade	Lesion stage and grade	Lesion stage and grade
Varies randomly across individuals	Yes	Yes	Yes
Varies randomly across same-size lesions within individuals	Yes	Yes	Yes
Nodal metastases explicitly modeled	Yes	No	No
Metastasis process explicitly modeled	Yes	Yes	Yes
*Mortality*			
Cancer deaths	Can only occur if metastasis occurs	Can occur if MIBC (T2/T3) or metastasis (T4) occurs	Can occur only if metastasis occurs
Other deaths	Same as general population	Same as general population	Risk of all-cause and cardiovascular deaths stratified by CKD stages
*Transitions to clinical disease*			
Mechanism	Time-to-event distribution	Stochastic function of lesion size	Age-dependent stochastic matrix
Symptoms modeled explicitly	No (only diagnosis modeled)	Microscopic or macroscopic hematuria, voiding symptoms	Microscopic or macroscopic hematuria (detectable state)
Varies systematically by	Sex, race, lesion T stage	Sex, race, lesion T stage	Sex, race, lesion T stage, lesion grade
Varies randomly across individuals	Yes	Yes	Yes
Varies randomly across same-size lesions within individuals	Yes	Yes	Yes
*Post-Tx recurrence and progression*			
Recurrence mechanisms			
Incomplete TUR/regrowth	No	Yes	No
New lesions	Yes	Yes	Yes
Missed lesions	Yes	Yes	Yes
Vary systematically by	Lesion histology, grade, T stage, lesion size, concomitant Tis, treatment	Lesion histology, grade, T stage, lesion size, treatment	Lesion grade, T stage, treatment
Progression mechanisms			
Transitions among preclinical new and missed lesions	Yes	Yes	Yes
Transitions among regrown lesions	No	Yes	No
Understaged lesions	Yes	Yes	Yes
Vary systematically by	Lesion histology, grade, T stage, lesion size, concomitant CIS, treatment	Lesion histology, grade, T stage, lesion size, treatment	Lesion grade, T stage, treatment
*Development*			
Software	R	R	Python
Uncertainty	Stochastic	Stochastic	Stochastic
Calibration	Bayesian	Bayesian	Bayesian

CIS = carcinoma in situ; CISNET = Cancer Incidence and Surveillance Modeling Network; CKD = chronic kidney disease; COBRAS = Cancer of the Bladder R-based Analytic Simulator; DES = discrete event simulation; HG = high grade; LG = low grade; MIBC = muscle-invasive bladder cancer; PUNLMP = papillary urothelial neoplasm of low malignant potential; SCOUT = Simulation of Cancers of the Urinary Tract; TUR = transurethral resection; Tx = treatment.

**Fig. 1:**
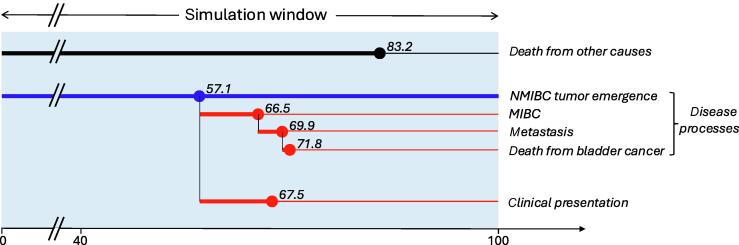
Key events in the natural history of bladder cancer considered in the three models. Simulated persons are followed up until they die from non–bladder cancer or bladder cancer causes. Some people will develop one or more bladder tumors at some point in their lives. As tumors grow, they may progress to the muscle-invasive disease, distal metastasis, and death from bladder cancer. As tumors grow and progress, these are more likely to be clinically detected. The figure shows an example of a sample path for a person who develops one tumor at 57.1 yr and dies from bladder cancer at 71.8 yr of age. In the schematic, each row represents an event process, that is, a mathematical submodel that generates the time of the next event if any occurs. Some event processes are arranged in series whereupon the next process starts only if an event occurred in the previous process (eg, the process of progressing to metastasis starts after the tumor becomes muscle invasive). Other events are competing, and their processes occur in parallel (eg, the death from non–bladder cancer causes competes with the disease processes). MIBC = muscle-invasive bladder cancer; NMIBC = non–muscle-invasive bladder cancer.

Figure 1 abstracts the key events of the natural history common to the three models. Individuals are simulated from birth and can develop bladder cancer with age-specific hazards calibrated to epidemiological data (see below). Some simulated people will develop one or more bladder tumors, which may progress to invasive and then to metastatic bladder cancer; mortality can occur from bladder cancer or other causes following age- and period-specific US mortality rates. Bladder cancer is initially undiagnosed (preclinical cancers), but may be diagnosed depending on persons’ race and sex, to account for observed racial discrepancies in the stage and mean age at diagnosis, cancer stage (all models) and tumor size (COBRAS and Kystis only), and bleeding or voiding symptoms (Kystis and SCOUT only).

All three models simulate smoking exposure. COBRAS and Kystis simulations follow historic smoking patterns in the USA, obtained from extensively verified reimplementation of CISNET’s Smoking History Generator (version 5.2.1) [17]. SCOUT’s smoking exposures are informed by National Health and Nutrition Examination Survey 2000–2018 smoking patterns among 40+ yr olds and do not vary by period.

### 2.2 Model input parameters, calibration, and verification

Model inputs or calibration targets include bladder cancer Surveillance, Epidemiology, and End-Results (SEER) program stage- and age-specific incidence, mortality, or relative survival data [18]; published and individual participant data from nine trials of early-stage bladder cancer from the European Organization for the Research and Treatment of Cancer, which inform recurrence and progression [19]; birth cohort–specific US all-cause mortality projections from the Centers for Disease Control and Prevention stratified by sex (female and male) and race (Black and White); US Census population estimates and projections [20]; and literature data identified in part through an ongoing bladder cancer evidence map [21]. The effects of treatment on bladder cancer mortality are not modeled explicitly in this analysis, which focuses on incidence outcomes. Mortality improvements due to improved patient management are not modeled explicitly, but these are captured implicitly through period-, age-, and stage-specific relative survival. In all the three models, the time interval from when a cancer is screen detectable (ie, when it first emerges in COBRAS and SCOUT, and when it reaches 2 mm in diameter for sessile and papillary lesions, and 5 mm for flat lesions in Kystis) to when it is diagnosed clinically is not modeled explicitly. This *sojourn time interval*, as it is usually called, is an emergent quantity.

All models are calibrated to the US SEER incidence data for 2010 using different optimization algorithms (Table 1) [22], [23]. Model development included extensive verification checks (eg, that simulated events are within biologically plausible ranges), logical checks (that models behave intuitively at extreme scenarios, eg, when the growth rate of tumors is very low), and dimensional analyses (that units in the left- and right-hand sides of all model equations match).

### 2.3 Simulation outcomes and analyses

We demonstrate calibration adequacy by comparing the simulated output versus the corresponding 2010 SEER estimates [18]. We simulated the 2010 US population as stacked cohorts of people born between 1910 and 2010 (covering 0–100 yr of age in 2010), with relative cohort sizes proportional to the census birth data. We then compared the simulated age-specific incidence in 5-yr age groups for all bladder cancer cases and by stage with the corresponding SEER data in 2010.

To better understand how the calibrated models differed in their simulation of the key natural history events (ie, tumor emergence, muscle-invasive bladder cancer [MIBC], metastasis, and diagnosis), we compared the age distributions at these events and the distributions of the time intervals between events for White men born in 1950 across models.

To describe population trends, we compared the models’ estimated lifetime risk of bladder cancer diagnosis for each birth cohort between 1900 and 2010. Note that the lifetime risks of each birth cohort are not directly observable, because no registry spans a birth cohort’s life length.

Finally, we use COBRAS and Kystis, the two models that account for evolving smoking patterns in the population, to project disease burden, that is, the age-standardized incidence (standardized to the US 2000 population [24]), annual incidence, and number of new cases of bladder cancer among White men between 2000 (when the surviving 1900 birth cohort becomes 100 yr old) and 2040, continuing the current trends and assuming that no screening will be introduced. This is done by extending the simulation of the US population to also include birth cohorts from 1900 through 2040.

Results of all the models are probabilistic because the occurrence and timing of the key events across simulations occur randomly. In addition, most input parameters are stochastic, that is, have a joint empirical distribution of the 1000 sets of best-fitting values in the calibration, which results in an uncertainty range in model outputs. We present results as mean prediction and range of predictions over this uncertainty set. The exceptions are the predictions of lifetime risk in the US population and the projections of bladder cancer burden, for which we projected only the predictions with the best-fitting parameter set. This is because of the large computational cost associated with repeating the latter analyses over the whole uncertainty set for parameters.

## 3 Results

### 3.1 Calibration to age- and stage-specific incidence in 2010

**Fig. 2:**
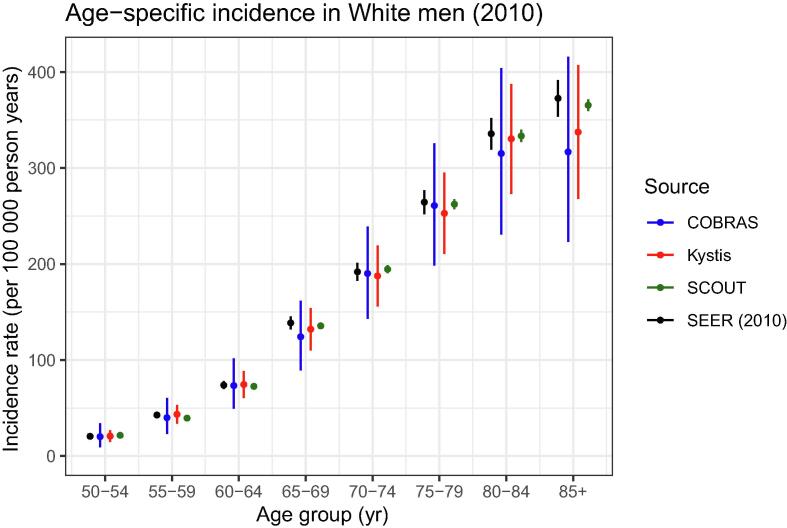
Age-specific incidence for US White men in 2010 (all models). Shown is the age-specific incidence for US White men in 2010 with the three models versus the SEER data. The models simulate the age composition of the US population of White men in 2010 by simulating cohorts of people born between 1910 (who would be 100 yr old in 2010 if they survive) and 2010, with proportions matching the relative numbers of births in each census year. Migration is ignored. The age-specific incidence is calculated as the number of new bladder cancer cases diagnosed in 2010 divided by the total number of person years observed in 2010 times 10^5^. Shown are 5-yr age groups starting at 50 yr, with those aged above 85 yr aggregated in a single group. The models agree with the SEER data for younger age groups as well (omitted to improve visualization in the chosen age ranges). The ranges are based on the joint empirical distribution of 1000 best-fitting calibrated parameters. COBRAS = Cancer of the Bladder R-based Analytic Simulator; SCOUT = Simulation of Cancers of the Urinary Tract; SEER = Surveillance, Epidemiology and End Results.

**Fig. 3:**
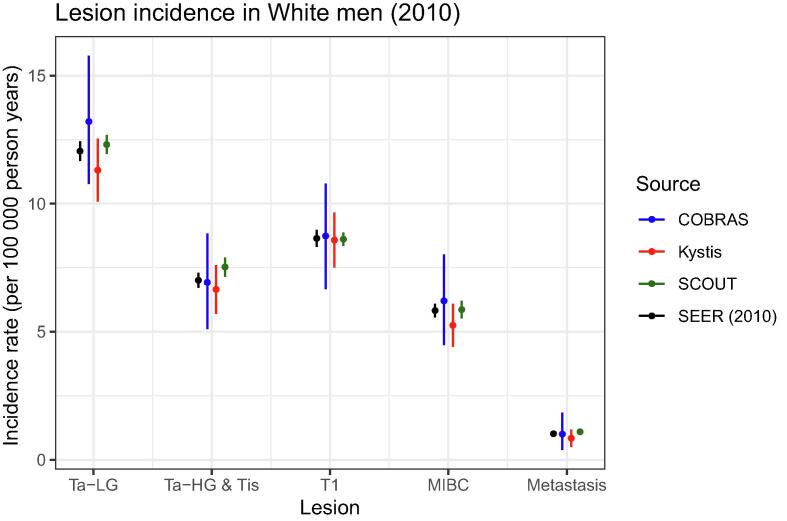
Incidence by lesion type for US White men 2010 (all models). Shown is the stage-specific incidence for US White men in 2010 with the three models versus the SEER data using a categorization common across all models. The models simulate the age composition of the US population of White men in 2010, as described in the legend of Figure 1. The ranges are based on the joint empirical distribution of 1000 best-fitting calibrated parameters. COBRAS = Cancer of the Bladder R-based Analytic Simulator; MIBC = muscle-invasive bladder cancer; SCOUT = Simulation of Cancers of the Urinary Tract; SEER = Surveillance, Epidemiology and End Results; Ta-HG = Ta high grade; Ta-LG = Ta low grade.

Figure 2 compares the simulated versus observed age-specific incidence for all cases of bladder cancer diagnosed in White men in 2010. Figure 3 compares the simulated versus observed stage-specific incidence in 2010 (see the Supplementary material for more granular categorizations per model). For almost all age groups and all models, the uncertainty intervals of the simulated and observed incidences overlap, indicating good agreement. The COBRAS and Kystis results have larger uncertainty in their projections than SCOUT results. This pattern persists in all analyses.

### 3.2 Lifetime risk of bladder cancer diagnosis by birth cohort

**Fig. 4:**
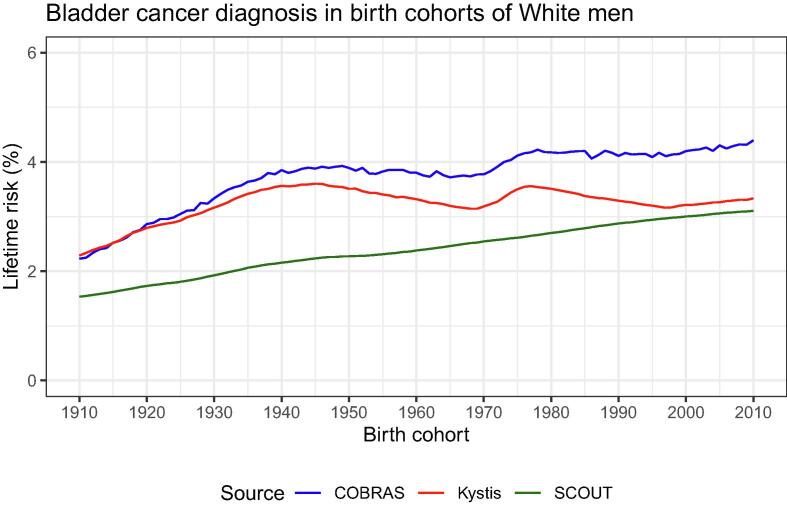
Lifetime risk of a bladder cancer diagnosis in White men per birth cohort. SCOUT lifetime risk estimates increase steadily from about 1.5% for the 1910 birth cohort to about 3.1% for the 2010 birth cohort. COBRAS and Kystis estimates increase from about 2.4% to about 3.9% and 3.6%, respectively, in the 1940–1950 birth cohorts, and then decline and peak again in the 1970–1980 birth cohorts. COBRAS and Kystis estimates are 4.4% and 3.3% in the 2010 birth cohort, respectively. Results are based on the best-fitting parameter set in the calibrations, to minimize computation cost (no uncertainty ranges depicted). COBRAS = Cancer of the Bladder R-based Analytic Simulator; SCOUT = Simulation of Cancers of the Urinary Tract.

Figure 4 compares the lifetime risk of being diagnosed with bladder cancer. The models estimated the lifetime risks of 1.5–2.4% for people born in 1910, which increased gradually following distinct patterns as life expectancy and smoking exposure rates for birth cohorts changed over time. The SCOUT estimates of the lifetime risk increased steadily to approximately 3.1% for the 2010 birth cohort. The COBRAS and Kystis estimates of the lifetime risk peaked at 3.9% and 3.6%, respectively, across the 1940–1950 birth cohorts, and then remained stable (COBRAS) or decreased (Kystis) and peaked again through the 1970–1980 birth cohorts. Their respective estimates in the 2010 birth cohort were 4.4% and 3.3%.

### 3.3 Epidemiological projections of disease burden

**Fig. 5:**
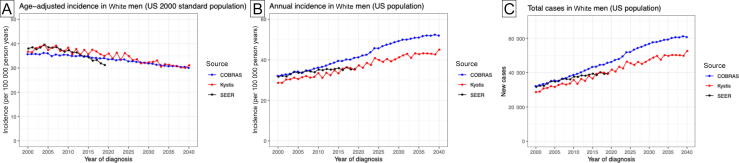
Projected population burden of bladder cancer in White men through 2040. (A) Projections of incidence standardized to the US 2000 standard population and (B) annual population incidence and (C) the number of new cases of bladder cancer in White men through 2040. Delay-adjusted SEER predictions for the age-standardized incidence in White men are shown in (A). SCOUT does not model time-varying smoking patterns and, thus, is not used in these projections. Results are based on the best-fitting parameter set in the calibrations, to minimize computation cost (no uncertainty ranges depicted). COBRAS = Cancer of the Bladder R-based Analytic Simulator; SCOUT = Simulation of Cancers of the Urinary Tract; SEER = Surveillance, Epidemiology and End Results.

Figure 5A shows the predicted age-standardized incidence of bladder cancer diagnosis with COBRAS and Kystis between 2000 and 2040, using the US 2000 standard population as a reference. The models’ predictions were qualitatively similar to the SEER data between 2000 and 2022, exhibiting a slight decrease in the age-standardized incidence. The SEER age-standardized incidence was constant through 2004, with a decline of 1.5%/yr between 2005 and 2022 [5]. COBRAS and Kystis projected smaller declines of, respectively, 0.4% and 0.6%/yr between 2005 and 2022. Their corresponding age-standardized incidence projections in 2040 were 30.0 and 31.1 new cases per 100K person years.

Age-standardized incidence removed the impact of demographic shifts in the population cohorts and does not fully reflect the actual age-related population burden of bladder cancer. Instead, Figure 5B projects the (crude) annual incidence of bladder cancer through 2040, as the age distribution of the population shifts to older ages. The SEER crude incidence changed from 31.7 to 35.5 cases per 100K person years between 2000 and 2010. In COBRAS and Kystis, the annual incidence of bladder cancer diagnosis increased continuously from 36–37 cases per 100K person years in 2010 (both models) to 52 (COBRAS) and 45 (Kystis) cases per 100K person years, respectively, in 2040. COBRAS and Kystis predicted that new cases continued to rise through 2040 (Fig. 5C), by 1.8%/yr and 1.5%/yr, respectively.

### 3.4 Ages at key events and important time intervals

Table 2 shows the median, and the 25th and 75th percentiles of the ages at which the key events in the natural history of bladder cancer occur and the time intervals between these key events for White men born in 1950. Across the three models, the first lesion appeared at median ages of 71–74 yr, MIBC first appeared at median ages of 75–77 yr, and metastasis first occurred at median ages of 77–79 yr. The median age of diagnosis ranged from 74 to 77 yr. The interquartile ranges for the ages of these key events in the population were wide and comparable across the three models.

**Table 2:** Ages of and time intervals between key events with each model (White men, 1950 cohort)

Description	COBRAS	Kystis	SCOUT
*Ages*			
First lesion emergence	71.0 (63.1, 77.9)	73.7 (64.5, 81.4)	72.7 (61.3, 81.2)
Diagnosis	76.8 (68.7, 83.7)	75.1 (66.4, 82.8)	74.0 (66.5, 81.0)
MIBC (T2)^a^	76.3 (68.6, 83.1)	76.6 (68.1, 83.7)	75.5 (68.4, 82.8)
Metastasis (T4)^a^	79.5 (71.7, 86.3)	77.2 (68.7, 84.3)	76.8 (69.8, 83.8)
*Time intervals*			
Emergence to diagnosis	2.1 (0.9, 3.7)	3.3 (2.8, 5.4)	3.1 (1.9, 4.9)
Emergence to MIBC^a^	1.3 (0.6, 2.6)	2.7 (1.6, 4.4)	2.9 (1.7, 4.6)
MIBC to metastasis^a^	2.6 (1.5, 4.2)	1.0 (0.4, 2.1)	1.4 (0.8, 2.2)

COBRAS = Cancer of the Bladder R-based Analytic Simulator; MIBC = muscle-invasive bladder cancer; SCOUT = Simulation of Cancers of the Urinary Tract.

Shown are median and (25th, 75th) percentiles for the ages of key events and time intervals between the key events among White men in the 1950 cohort. Results from simulations using the best fitting parameter sets.

a For COBRAS, the ages at T2 and T4 are used for “age at MIBC” and “age at metastasis,” respectively.

The median interval between the time when a lesion is screen detectable and the time of actual diagnosis fell between 2.1 and 3.3 yr, but with broad interquartile ranges. The median time to MIBC from lesion emergence ranged between 1.3 and 2.9 yr, and the interval from MIBC to metastasis was between 1.0 and 2.6 yr, again with a wide distribution across simulated individuals (Table 2).

## 4 Discussion

The models help explain the historical population trends of bladder cancer. The age-standardized incidence in SEER was approximately constant at 38–39 new cases per 100K person years before 2005, decreasing thereafter by approximately 1.5%/yr through 2022, ostensibly reflecting decreased smoking exposure since the 1980s [18]. Some authors have observed apparent incongruity in the age-standardized incidence patterns between bladder and other smoking-related cancers. The age-standardized incidence patterns of bladder cancer, compared with that of lung cancer, lag by about two decades and decline much less steeply [25]. COBRAS and Kystis modeling replicated the observed lag in the start of the decline and predicted that the decline should have a small magnitude.

At the same time, the burden of bladder cancer, in terms of its (crude) incidence and number of new diagnoses for White men (the group that makes up the majority of the bladder cancer population) is projected to increase. In additional analyses that are not presented here, the models projected similar increases in Black men, and White and Black women. By living longer, people increase their risk of developing bladder tumors later in life. This longevity more than offset the effect of declining smoking rates. All this implies that more resources, in terms of infrastructure, personnel, and research, may be required to manage bladder cancer in the future. The USA is currently facing a severe overall shortage of urologists [26], which is accentuated by an aging and geographically maldistributed workforce and is projected to worsen in the future [27]. The median age of practicing urologists is 55 yr, with 30% of them being 65 yr or older. As the number of new urologists is decreasing, this trend is projected to accentuate [27]. Further, most urology specialists are in urban locations, with 60% of US counties having no practicing urologist. For bladder cancer, a serious disease that often requires specialist interventional care, urologists are a de facto inelastic resource and relying on nonurologist providers is impractical. Policies that help address this emerging problem include, but are not limited to, incentivizing new physicians to become urologists and more urologists to also manage bladder cancer, and, possibly, shifting the management of other urological conditions to nonurologist providers.

In the USA, no systematic screening or other early detection effort for bladder cancer exists [28]. We did not perform dedicated modeling to explore whether and how early-detection efforts affect mortality and other clinically relevant outcomes, to assess the risk of overdiagnosis and overtreatment of indolent disease, or to estimate their potential public heath utility, including cost effectiveness. All these are the object of upcoming work. However, according to the models, 75% of bladder cancer cases could become screen detectable after the ages of 61–64 yr, and the median interval between the first time a lesion is detectable and its clinical detection is approximately 2.1–3.3 yr. Thus, there is a theoretical prospect for early detection of bladder cancer. This conjecture agrees with high-level hypothetical analyses based on aggregate epidemiological data [8], and with the conclusions of earlier and simpler models on the utility and cost effectiveness of various screening technologies [29], [30]. For example, in a recent cost-effectiveness analysis, one-time urine dipstick screening for hematuria in current and former smokers was cost effective in the UK setting [31]. Therefore, the role of early detection and control should be explored more systematically with dedicated modeling, and possibly, with empirical studies.

The three models made different assumptions about the natural history of bladder cancer and the role of risk factors. Kystis and COBRAS agree qualitatively, and often quantitatively, in their predictions of historical lifetime risks and bladder cancer burden through 2040. This comparative modeling result suggests that, despite their many differences, the models capture similar disease and risk factor dynamics at the population level. SCOUT’s predictions of historical lifetime risks are similar to those of the other two models in terms of the order of magnitude but exhibit a different time pattern. We attribute these differences to SCOUT not modeling the evolution of historical smoking patterns in the USA, assuming instead an exposure distribution that is constant over time. Distinct versions of SCOUT will incorporate detailed temporal patterns for smoking.

### 4.1 Limitations

With only limited data from screening studies [28] and from incidental findings of bladder lesions [32], direct data on the time between the emergence of bladder lesions and clinical cancer do not exist. This time interval, the sojourn time, is critical for deducing the potential benefit from early detection and the timing of screening. Although a range of sojourn times is compatible with the limited data, modeling helps bound sojourn time estimates through calibration to the incidence data.

Exposure to smoking is the most important modifiable risk factor for bladder cancer at the population level. Smoking rates have decreased since the 1960s, but vaping has recently become more popular. Evidence of increased carcinogen biomarkers in the urine of vaping enthusiasts suggests that electronic cigarette use is a risk factor [33]. We do not account for these recent trends, but the predicted combustible smoking rates are already generally low, so making them even smaller will have limited impact. Nonetheless, we will explore the projected impact of recent smoking and vaping trends on future bladder cancer outcomes in future work.

In the simulation scenarios, COBRAS and Kystis predict an annual decline in the age-specific incidence of bladder cancer that averages to 0.4–0.6%/yr between 2005 and 2022, which is smaller than the SEER trend of 1.5%/yr over the same period. The models were calibrated to the SEER data from 2010 only. Their longitudinal predictions emerge from the modeled historic mortality and smoking patterns, and cannot fully explain the longitudinal trends in the age-adjusted incidence. Apart from smoking, several other carcinogens can cause bladder cancer, albeit with smaller attributable risks [4], [34]. Our models do not yet include the impact of environmental pollutants or occupational and household exposures to other carcinogens, which may become more important as smoking rates decrease [4]. Incorporation of period effects that capture changes in unmeasured exposures (eg, occupational risks) is an important direction for future work.

We did not explicitly model the effects of bladder cancer management on disease progression or mortality, because we only focused on the projection of cancer incidence–related outcomes. Modeling cancer management and its downstream effects is necessary for assessing the outcomes of screening or carcinogen control policies, but this is out of scope for this work.

Finally, predictions with all three models involve uncertainty, which, at least in part, results from uncertainties in inputs and parameters. For example, because of the lack of screening data, there is not enough information to fully identify the natural course of bladder cancer before its clinical diagnosis: For many model parameters, there are whole ranges of values that comport with epidemiological data. In an attempt to include parameter uncertainty, we retained the 1000 best fitting parameter sets in our models’ calibrations. Even so, we are almost certainly understating the models’ predictive uncertainty substantially. The characterization and propagation of different types of uncertainty (aleatory, stemming from finite samples, vs epistemic, stemming from our incomplete understanding of the modeled system) constitute an open problem. For some analyses that are resource intensive, we present results from the best fitting parameter sets only. Approximation techniques to uncertainty propagation exist, but these are not used in this application, where we deemed that the main takeaway is about the models’ qualitative behavior [35], [36], [37].

## 5 Conclusions

Our models project that the annual incidence of bladder cancer and new cases will increase at least through 2040. Increased exposure to environmental pollutants, vaping, and occupational and household carcinogens may accentuate these trends. If future cases consume approximately the same resources as the current cases, the increase in burden implies that commensurate increases in infrastructure, personnel, and money will be necessary to provide quality care. This is troublesome given the current and projected shortage of urologists in the USA. From the inferred natural history, we speculate a theoretical opportunity for screening, which should be investigated with dedicated modeling and empirical studies.

  ***Author contributions:*** Thomas A. Trikalinos had full access to all the data in the study and takes responsibility for the integrity of the data and the accuracy of the data analysis.

  *Study concept and design*: Jalal, Kang, Alarid-Escudero, Jacobs, Kuntz, Wong, Trikalinos.

*Acquisition of data*: Jalal, Garibay-Trevino, Kumar, Popp, Sereda, Siriruchatanon, Trikalinos.

*Analysis and interpretation of data*: Jalal, Kang, Alarid-Escudero, Chrysanthopoulou, Popp, Sereda, Siriruchatanon, Trikalinos.

*Drafting of the manuscript*: Jalal, Trikalinos.

*Critical revision of the manuscript for important intellectual content*: Jalal, Kang, Alarid-Escudero, Chrysanthopoulou, Garibay-Trevino, Jacobs, Kuntz, Kumar, Popp, Sereda, Siriruchatanon, Wong, Trikalinos.

*Statistical analysis*: Jalal, Kang, Alarid-Escudero, Chrysanthopoulou, Popp, Sereda, Siriruchatanon, Trikalinos.

*Obtaining funding*: Jalal, Kang, Trikalinos.

*Administrative, technical, or material support*: Garibay-Trevino, Kumar, Sereda, Popp, Siriruchatanon.

*Supervision*: Jalal, Kang, Alarid-Escudero, Trikalinos.

*Other*: None.

  ***Financial disclosures:*** Thomas A. Trikalinos certifies that all conflicts of interest, including specific financial interests and relationships and affiliations relevant to the subject matter or materials discussed in the manuscript (eg, employment/affiliation, grants or funding, consultancies, honoraria, stock ownership or options, expert testimony, royalties, or patents filed, received, or pending), are the following: None.

  ***Funding/Support and role of the sponsor:*** The COBRAS and Kystis models were developed in the context of the Cancer Incidence and Surveillance Modeling Network (CISNET) program of the National Cancer Institute (NCI) of the National Institutes for Health (U01CA265750, principal investigators [PIs] Thomas A. Trikalinos and Hawre Jalal), and SCOUT is an NCI-funded affiliate research effort (R01CA262375, PI Stella K. Kang). Together they comprise the CISNET Bladder Cancer Incubator Site. The results described here are based on COBRAS version 1.0.0, Kystis version 0.1.7.9004, and SCOUT version 1.0.0.
