# Supplementary material for: Bladder Cancer Burden in the USA: Population Scenarios for 2040
Source: Eur Urol Open Sci. 2025 Dec 4;83:99–108. doi: 10.1016/j.euros.2025.11.013 (PMC12721285; doi:10.1016/j.euros.2025.11.013)
Supplement: Supplementary Data 1 [file mmc1.pdf]

COBRAS (University of Ottawa)

BLADDER

University of Ottawa
MPD Version:
1.0.00

uOttawa

Reader's Guide

Model Purpose

Model Overview

Assumption Overview

Parameter Overview

Component Overview

Output Overview

Results Overview

Key References

Cancer of the Bladder R-based Analytic Simulator (COBRAS)

University of Ottawa

Contact

Hawre Jalal (hjalal@uottawa.ca)

Suggested Citation

Forthcoming.

Version Table

Version	Release Date	Notes	Publications
1.0.00	2025-02-27	Initial version of the micro-simulation model (bladder cancer natural history).	N/A

All material © Copyright 2025 CISNET

University of Ottawa
Readers Guide

uOttawa

Reader's Guide
Model Purpose
Model Overview
Assumption Overview
Parameter Overview
Component Overview
Output Overview
Results Overview
Key References

Reader's Guide

Core Profile Documentation

These topics will provide an overview of the model without the burden of detail. Each can be read in about 5-10 minutes. Each contains links to more detailed information if required.

Model Purpose
This document describes the primary purpose of the model.

Model Overview
This document describes the primary aims and general purposes of this modeling effort.

Assumption Overview
An overview of the basic assumptions inherent in this model.

Parameter Overview
Describes the basic parameter set used to inform the model, more detailed information is available for each specific parameter.

Component Overview
A description of the basic computational building blocks (components) of the model.

Output Overview
Definitons and methodologies for the basic model outputs.

Results Overview
A guide to the results obtained from the model.

Validation Overview
A guide to the model validation.

Key References
A list of references used in the development of the model.

Further Reading

These topics will provide a intermediate level view of the model. Consider these documents if you are interested gaining in a working knowledge of the model, its inputs and outputs.

Advanced Reading

These topics denote more detailed documentation about specific and important aspects of the model structure.

All material © Copyright 2025 CISNET

uOttawa

Reader's Guide
Model Purpose
Model Overview
Assumption Overview
Parameter Overview
Component Overview
Output Overview
Results Overview
Key References

Model Purpose

Summary

The Cancer of Bladder R Analytic Simulator (COBRAS) is an individual-level discrete event simulation (DES) model designed to simulate the onset, progression, detection, recurrence, and treatment of bladder cancer in the U.S. population. The model was developed to provide insights into, and inform optimal health policy strategies for, reducing the incidence and mortality of bladder cancer.

Purpose

COBRAS is intended as a tool to assess bladder cancer development, as well as the costs and outcomes associated with various interventions aimed at lowering bladder cancer incidence and mortality in the United States. These interventions can include new screening regimens and the adoption of emerging detection tests.

The model's key aims are:

1. Estimate natural history parameters that govern bladder cancer development in a simulated population.
2. Quantify disease burden under changing demographic conditions, such as an aging population.
3. Evaluate the effectiveness, costs, and cost-effectiveness of a range of health strategies, including screening, surveillance, and treatment.

By integrating data on disease progression, screening performance, and treatment outcomes, COBRAS can help guide decisions about how best to allocate resources and implement policies that diminish the burden of bladder cancer in the United States.

All material © Copyright 2025 CISNET

uOttawa

Reader's Guide
Model Purpose
Model Overview
Assumption Overview
Parameter Overview
Component Overview
Output Overview
Results Overview
Key References

Model Overview

Summary

This section provides an overview of the COBRAS model's structure and its major components.

Purpose

The COBRAS model was developed to evaluate different screening and surveillance programs by comparing the cost, effectiveness, and cost-effectiveness of various bladder cancer detection and treatment strategies. To achieve this, COBRAS simulates the natural history of bladder cancer in the U.S. population and calibrates its underlying disease parameters using SEER data.

By modeling how bladder cancer lesions emerge in each individual, how they progress, and how death occurs from bladder or other causes, the COBRAS model provides insights into the potential impact of alternative policies aimed at reducing bladder cancer incidence and mortality.

Background

Bladder cancer predominantly affects older adults, with nine out of ten cases occurring in individuals older than 55 years of age, and the average age at detection being 73 [1]. Men experience bladder cancer at a rate four times higher than women [1]. Additionally, White men have twice the incidence compared with Black, Hispanic, or Asian/Pacific Islander men [1]. It remains uncertain whether these differences stem from variation in risk exposures, biological factors, or disparities in access to medical care.

Model Description

The COBRAS model is an individual-level discrete event simulation (DES) framework that generates a U.S. synthetic population and projects bladder cancer incidence and mortality. It is implemented in the R programming language [2].

Each simulated individual is assigned race, sex, and birth year, along with a time of death from causes other than bladder cancer. The model also determines who develops lesions, how many they develop, and each lesion's initial stage at onset. Based on this information, COBRAS simulates the time to death from bladder cancer using a fast vectorized algorithm [3].

The baseline version of COBRAS generates birth cohorts from 1900 onward, combining them up to create a population snapshot in the calendar year 2010 for analysis. This can be adapted to simulate cohorts up to 2060 or other time horizons. The model comprises the following components (**Figure 1**):

- a birth cohort generator module,
- a smoking history generator,
- a background mortality module,
- a tumor generator module,
- a tumor growth module,
- a nodal involvement and distant metastasis module,
- a diagnosis module,
- a bladder cancer mortality module, and
- a post diagnosis treatment and surveillance module.

All material © Copyright 2025 CISNET

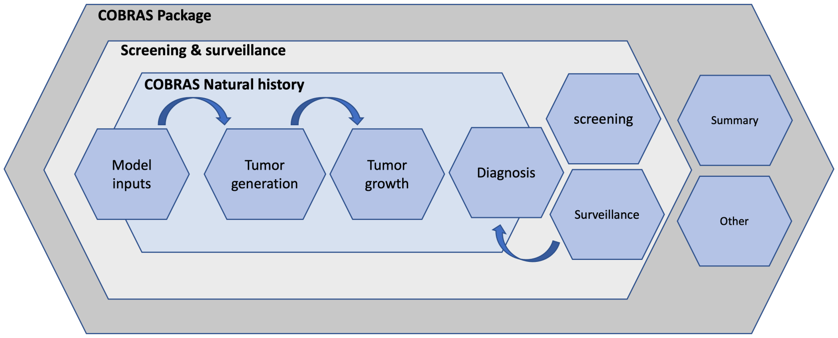

Figure 1: COBRAS model structure.

Additional details on population demographics, the natural history of bladder cancer, and the specified screening and surveillance programs appear in the Assumption Overview. The Parameter Overview describes the variables used to model natural history, screening, and surveillance.

References

1. National Cancer Institute. Surveillance, Epidemiology, and End Results Program: Cancer Stat Facts, Bladder Cancer [Internet]. 2024. Available from: https://seer.cancer.gov/statfacts/html/urinb.html/
2. R Core Team. R: A Language and Environment for Statistical Computing [Internet]. Vienna, Austria: R Foundation for Statistical Computing; 2025. Available from: https://www.R-project.org/
3. David U Garibay-Treviño, Hawre Jalal, Fernando Alarid-Escudero. A Fast Nonparametric Sampling Method for Time to Event in Individual-Level Simulation Models. Medical Decision Making. SAGE Publications Sage CA: Los Angeles, CA; 2025;0272989X241308768.

uOttawa

Reader's Guide
Model Purpose
Model Overview
Assumption Overview
Parameter Overview
Component Overview
Output Overview
Results Overview
Key References

Assumption Overview

Summary

This section outlines the key assumptions of the COBRAS Model, which are informed by both scientific literature and clinician expertise.

Background

The COBRAS model rests on several fundamental assumptions regarding bladder cancer pathogenesis and progression. It is structured to be flexible for sex- and race-specific differences yet retains a uniform modeling approach for all population subgroups.

Assumption Listing

Demographic Assumptions

Background Mortality

COBRAS samples each individual's background mortality age using U.S. Life Tables. Year-, sex-, race-, and age-specific mortality probabilities are employed in a categorical distribution to determine the age of death from causes unrelated to bladder cancer.

Natural History Assumptions

Risk of Bladder Cancer

COBRAS uses a binomial process to designate which individuals develop bladder cancer:

$$BC_i \sim \text{Binomial}(P(BC_i))$$

where the probability of developing bladder cancer in the i-th individual is given by:

$$P(BC_i) = \frac{1}{1 + \exp(-\text{odds}_i)}$$

and

$$\text{odds}_i = \alpha_0 + \alpha_1 \cdot \text{sex}_i + \alpha_2 \cdot \text{race}_i + \alpha_3 \cdot \text{smoking}_i$$

- α_0 is baseline odds for white males.
- α_1 reflects the odds difference for females relative to males (sex_i = 0 for males, 1 for females).
- α_2 is the odds difference for black individuals vs. white individuals.
- α_3 is the odds difference for smokers vs. nonsmokers.

Tumor Generation

For individuals who develop bladder cancer, the number of tumors (c_i) follows a zero-truncated Poisson distribution:

$$c_i \sim \text{Trunc Poisson}(\lambda_i | a = 0)$$

with

$$\log(\lambda_i) = \beta_{0_i} + \beta_{\text{sex}} \cdot \text{sex}_i + \beta_{\text{race}} \cdot \text{race}_i + \beta_{\text{smoking}} \cdot \text{smoking}_i$$

The baseline risk (β_{0_i}) has a normal distribution:

$$\beta_{0_i} \sim \text{Normal}(\beta_0, \sigma^2_{\beta_0})$$

All material © Copyright 2025 CISNET

Age of Patient at Lesion Onset

The age of patient at lesion onset is modeled using a Weibull distribution:

$$\text{age of patient at lesion onset}_i \sim \text{Weibull}(\tau, \theta_i)$$

where the individual-specific scale (θ_i) is drawn from a lognormal distribution:

$$\theta_i \sim \text{Lognormal}(\mu_{\text{scale}}, \sigma_{\text{scale}})$$

Allowed Initial Tumor Types

Individuals may initially develop Tis, Ta low-grade (Ta-lg), or Ta high-grade (Ta-hg) tumors (see **Figure 1**). The onset tumor type is assigned via a categorical distribution:

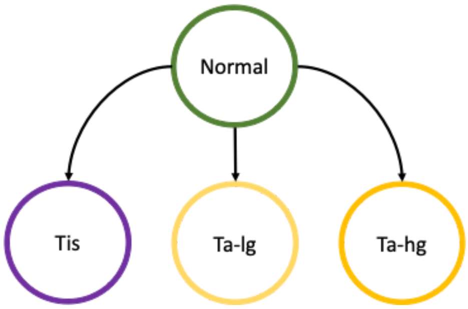

Figure 1: Allowed initial tumor types in the COBRAS model.

$$\text{type at onset} \sim \text{Categorical}(P_{\text{Tis}}, P_{\text{Ta-lg}}, P_{\text{Ta-hg}})$$

where P_{Tis} and P_{Ta-hg} are estimated from the literature, and $P_{\text{Ta-lg}} = 1 - (P_{\text{Tis}} + P_{\text{Ta-hg}})$

Possible Transitions

Once the initial tumor type is set, transitions among clinical and preclinical tumor stages occur as depicted in **Figure 2**.

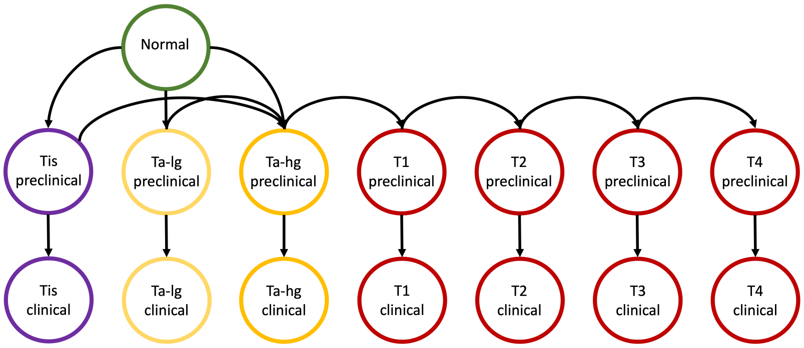

Figure 2: Allowed transitions between clinical and preclinical stages in the COBRAS model.

Tumor Growth

Tumors are classified as muscle-invasive bladder cancer (MIBC) or non-muscle-invasive bladder cancer (NMIBC). MIBC growth rates are exponential and stage-specific, while NMIBC growth follows a Gompertz model. These parameters are calibrated to SEER data.

For MIBC, we assume an exponential transition rate between tumor stages. These rates are T-stage-specific and assumed to be higher for the more advaced stages.

For individuals who develop Tis tumors, we consider a multiplicative hazard ratio applied to the MIBC growth rate. This hazard ratio was implemented considering that individuals with Tis have a different tumor development progression behavior.

For NMIBC, we assume that tumors have a spherical shape and that the tumor sizes areis defined by the diameter of the sphere. The speed of NMIBC growth is defined by a Gompertz distribution considering that all tumors have an initial size of 1 millimeter. In the model we obtain the time to arrive to the following NMIBC sizes: 0.5 centimeters (cm), 1 cm, 3 cm, 5 cm, and 8 cm. The asymptote of the Gompertz growth curve represents maximum NMIBC size, and currently has a value of 2,150 cubic centimeters, which comes from a diameter of approximately 16 cm.

Nodal Involvement and Metastasis

Lymph node involvement is modeled as a two-step process: No involvment (N0) to local involvement (N1) to distant involvement (N2). These transition rates follow exponential functions and are calibrated. Similarly, metastasis is a binary variable, with time to metastasis drawn from an exponential distribution.

Bladder Cancer Diagnosis (Detection)

MIBC tumors are detected based on stage-specific exponential rates. Detection rates for Tis, Ta-lg, Ta-hg and T1 are assumed to be unknown and are calibrated. For MIBC stages T2, T3, and T4 are derived using log hazard ratios to ensure ordinal detection rates relative to T1, such that

$$\log(r_{T_{i+1}}) = \log(r_{T_i}) + \log(\mathrm{HR}_{i+1}).$$

Competing risks are considered during detection. A tumor will be detected in a particular stage if the time to detection is lower than the transition time to the next stage.

Once a tumor is detected in an individual, we assume that this individual will have a test to detect the rest of the tumors. Currently we assume a 95% test sensitivity for all tumor types. The same test sensitivity is applied during surveillance visits.

With the information of all the detected tumors, the patient is classified for surveillance considering the most advanced detected tumor, and their metastasis and nodal involvement status of the detected tumors at symptom detection.

Bladder Cancer Mortality

Bladder cancer mortality is determined using stage-specific exponential rates driven from SEER relative risk of survival. The age of death is the minimum of the background mortality age and the bladder cancer mortality age.

Surveillance Assumptions

Surveillance Schedules

Patients are classified into risk categories based on the NCCN Clinical Practice Guidelines. Surveillance schedules are defined as follows:

- **Low Risk**: Solitary Ta-lg tumor ≤ 3 cm.
- **Intermediate Risk**: Includes solitary Ta-hg tumor ≤ 3 cm, multifocal Ta-lg, Ta-lg recurrence within a year, or Ta-lg > 3 cm.
- **High Risk**: Includes T1/CIS lesions, multifocal Ta-hg, Ta-hg recurrence within a year, or Ta-hg > 3 cm.

Surveillance schedules for each risk category are summarized in **Table 1**:

Table 1. Surveillance schedules by risk category

A. Low-risk patients

Years	Months
1	3 & 12
2 to 5	Annually
10+	As clinically indicated

B. Intermediate-risk patients

Years	Months
1	3, 6 & 12
2	Every 6 months
3 to 5	Annually
5+	As clinically indicated

C. High-risk patients

Years	Months
1 to 2	Every 3 months
3 to 5	Every 6 months
5 to 10	Annually
10+	As clinically indicated

During each surveillance visit, the following steps are performed:

1. Confirm the patient is alive.
2. Verify attendance at the appointment.
3. Check for undetected tumors.
4. Detect any new tumors and redefine the patient's classification if necessary.

These steps are summarized in **Figure 3**.

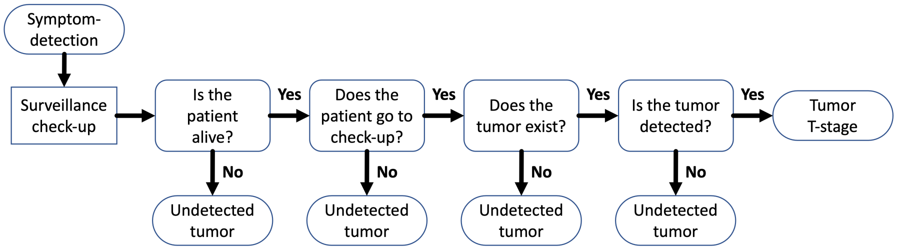

Figure 3: Surveillance flowchart.

The probability of attending a surveillance visit and detecting new tumors is modeled using binomial distributions. Test sensitivity remains consistent with the natural history module.

uOttawa

Reader's Guide
Model Purpose
Model Overview
Assumption Overview
Parameter Overview
Component Overview
Output Overview
Results Overview
Key References

Parameter Overview

Summary

This document provides an overview of the demographic and natural history parameters used in the COBRAS model.

Background

TCOBRAS integrates two major parameter sets: demographic parameters (which specify characteristics of a simulated population, such as race, sex, and mortality) and natural history parameters (which govern how bladder cancer develops and progresses). Some of these natural history parameters also affect other processes, such as tumor detection during surveillance.

Parameter Listing Overview

Population demographics

COBRAS assigns each simulated individual a birth year, race, sex, smoking status, and an age of death due to causes other than bladder cancer. These demographic variables reflect U.S. population trends and are based on published life tables [1]. Each individual is assumed to have a single outcome for background mortality (i.e., an age of death unrelated to bladder cancer).

Currently, COBRAS includes only two race categories (Black and White) and two sex categories (male and female). Future expansions may incorporate additional categories. Smoking status is binary (smoker vs. non-smoker) and is currently set to “non-smoker” in the baseline version. Population size, as well as the distribution of sex and race, are calibrated against empirical data.

Natural history parameters

All of COBRAS’s natural history parameters fall into several conceptual areas:

1. **Risk of Developing Bladder Cancer**
   Each individual has a probability of developing bladder cancer that reflects demographic differences (e.g., sex, race) and possible behavioral factors such as smoking.

2. **Number of Tumors**
   Once an individual is designated to develop bladder cancer, the model determines how many tumors appear. This number can vary due to differences in baseline risk across the population.

3. **Age at Lesion Onset**
   The time at which bladder lesions appear follows a distribution that allows for variability among individuals. COBRAS can incorporate both group-level differences (such as sex or race) and person-level random variation.

4. **Initial Tumor Types**
   When a tumor is first created, it may present as one of several possible stages, such as Ta (low- or high-grade) or Tis. The probability of each initial stage is determined by empirical data.

5. **Tumor Progression**

   - **Muscle-Invasive Bladder Cancer (MIBC)**: Progresses through clinical stages via exponential transitions. A hazard ratio can be applied to certain subgroups (e.g., Tis) to capture faster or slower progression.

- **Non–Muscle-Invasive Bladder Cancer (NMIBC)**: Uses a growth curve to capture changes in tumor volume over time.

6. **Nodal Involvement and Metastasis**
   Time to lymph node involvement (N1, N2) and time to distant metastasis are each modeled with exponential rates.
7. **Detection by Symptoms**
   Symptomatic detection occurs when a tumor has progressed sufficiently to cause clinical signs. Different stages (T1, T2, T3, T4) may have different detection rates.
8. **Test Sensitivity**
   When the model simulates a clinical exam (for example, during screening or surveillance), detection probabilities vary by tumor stage, reflecting differences in test performance.
9. **Bladder Cancer Mortality**
   The model applies stage-specific mortality rates to each tumor type. Individuals may die from bladder cancer or from other causes, whichever occurs first.

Surveillance parameters

During surveillance, patients will have appointments to be checked for new tumors. The surveillance schedule inside the COBRAS model has been defined by following the The model includes three levels of patient risk during surveillance: low, intermediate and high risk. The risk-specific schedules are defined by the following parameters:

Following detection or diagnosis, patients enter a surveillance schedule. COBRAS adapts the NCCN Clinical Practice Guidelines schedules[2] with risk-based screening intervals for follow-up:

- **Low risk:** Follow-ups at longer intervals.
- **Intermediate risk:** More frequent follow-ups than low risk.
- **High risk:** The most frequent follow-up schedule.

At each visit, a patient has a certain probability of attending (adherence), and any new or progressing tumor may be detected according to the same stage-specific test sensitivities. If newly detected lesions are found, the patient's risk category—and thus their follow-up schedule—can change accordingly.

Calibrated parameters

COBRAS includes both calibrated and fixed parameters:

- **Calibrated parameters** are estimated by matching model outputs to population-level data (e.g., SEER). These include the parameters governing tumor progression rates and the symptomatic detection process.
- **Fixed parameters** use direct estimates from literature or expert consensus. Examples include certain baseline mortality rates and default test sensitivities.

An example of a calibrated parameter is the transition rate from Tis to T1, which is estimated via calibration. An example of a fixed parameter is the maximum tumor volume assumed for NMIBC growth.

Table 1 provides a high-level look at the main parameters, indicating which ones are calibrated or fixed, as well as their plausible ranges or baseline values. (See the full table for specific numerical bounds.)

Table 1. Calibrated parameters in the COBRAS model

Parameter Group	Example Variables	Calibrated?	Notes
BC Risk & Incidence	Sex/race differences, smoking status	Yes	Key driver of who develops cancer

Parameter Group	Example Variables	Calibrated ?	Notes
Tumor Generation	Number of tumors, initial stage probabilities	Yes	Zero-truncated Poisson approach
Age of Onset	Distribution shape/scale for lesion onset	Yes	Uses Weibull or log-normal combo
Progression Rates	MIBC stage transitions, NMIBC growth parameters	Yes	Typically exponential or Gompertz
Detection Rates	Stage-specific detection by symptoms or tests	Yes	Calibrated to reflect real-world
Mortality Rates	Stage-specific hazard of BC death	No	Often derived from SEER data
Surveillance	Schedule intervals, adherence probability	No	Based on guidelines & assumptions

References

1. Elizabeth Arias, Jiaquan Xu, Kenneth Kochanek. United States life tables, 2021. 2023;
2. National Comprehensive Cancer Network, others. NCCN clinical practice guidelines in oncology. http://www nccn org/professionals/physician_gls/PDF/occult pdf. 2008;

Reader's Guide
Model Purpose
Model Overview
Assumption Overview
Parameter Overview
Component Overview
Output Overview
Results Overview
Key References

Component Overview

Summary

This section describes the major components of the COBRAS model, illustrating how each module interacts to simulate the development, detection, and outcomes of bladder cancer at the population level.

Overview

COBRAS simulates a population of individuals (by age, sex, race, and smoking history), follows them over time, and projects the incidence and mortality of bladder cancer. COBRAS is a population model, meaning it starts simulating each birth-year cohort starting in 1900. The outcomes in each calendar year is created by combining outcomes for the cohorts that contribute to the U.S. population composition in that year. For example, the population outcome for the calendar year 2010 involves birth-year cohorts born between 1910 and 2010.

COBRAS uses a series of interconnected modules, each responsible for a key aspect of disease progression, detection, and patient follow-up. The overall structure is presented in **Figure 1**.

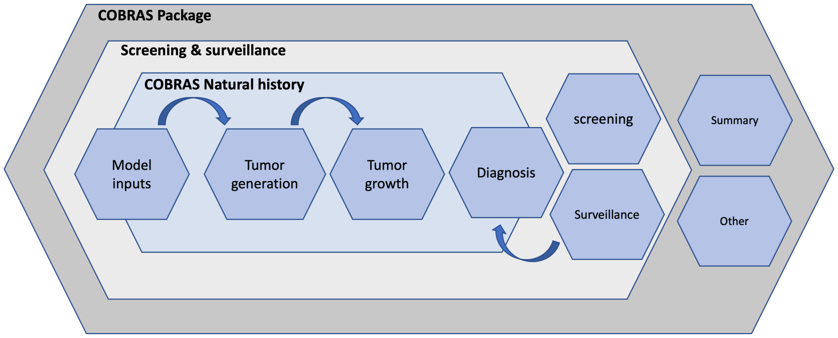

Figure 1: COBRAS model structure.

Currently, COBRAS includes the following components:

- A **population generator** to create a synthetic cohort including their history of smoking
- A **bladder cancer natural history** module
- A **mortality** module
- A **surveillance** module
- A **summary** module of key results

A **screening** module is planned for future development.

Component Listing

Population generator

The population generator produces a synthetic cohort that reflects demographic characteristics (race, sex, birth year) and smoking histories. It also assigns a background mortality age for each individual based on external life table data. Individuals are then followed in the simulation according to their demographic traits and mortality risk. In addition, smoking history for each individual is obtained from the Smoking History Generator.

Bladder Cancer natural history

All material © Copyright 2025 CISNET

The natural history module determines:

1. **Who develops bladder cancer** among the simulated population.
2. **How many tumors** each affected individual generates.
3. **Tumor characteristics**, including initial stage (e.g., Tis, Ta-low-grade, Ta-high-grade), growth patterns (muscle-invasive vs. non–muscle-invasive), and potential transitions to more advanced stages.

- **Muscle-invasive growth** is assumed stage-specific and can involve hazard ratios for tumors originating as Tis.
- **Non–muscle-invasive growth** follows a Gompertz-like curve, treating tumors as spherical until they reach a pre-defined maximum size.
- **Nodal involvement and metastasis** are allowed for tumors that start at Tis or Ta-high-grade, though these do not currently alter mortality rates or other natural history processes beyond their point of detection.

The module simulates **symptomatic detection** using stage-specific detection rates. If a tumor is detected, the individual undergoes an evaluation that can identify additional tumors. Detection triggers a risk classification process based on guidelines [1], and the individual's time to death from bladder cancer is set, using stage-specific mortality rates starting from the time of detection.

Mortality module

This module integrates:

- **Background mortality** from U.S. Life Tables (e.g., from heart disease or other causes).
- **Bladder cancer mortality** (time from symptomatic detection to death).

Each individual's final age of death is the minimum of these two ages. If an individual never develops bladder cancer, their cause of death is attributed to non-bladder causes.

Surveillance module

Once a tumor is detected, the individual enters a surveillance schedule with periodic checkups. The frequency of these visits depends on the patient's risk category at the time of detection. During each surveillance visit:

1. The individual may or may not attend (based on an adherence probability).
2. Additional tumors can be detected, potentially changing the patient's risk classification.
3. Surveillance continues until either the scheduled visits are completed or the patient dies.

Screening module

A formal screening module has not yet been implemented. Future releases of COBRAS will incorporate population-based screening strategies, enabling comparisons of different screening intervals and test modalities.

Summary module

Finally, COBRAS aggregates model outputs to facilitate validation and policy analysis. Key outcomes include:

- **Age-specific incidence rates**
- **Stage-specific incidence rates**
- **Proportion of tumors by stage**
- **Lifetime risk of bladder cancer**
- **Age of tumor onset and symptomatic detection**
- **Sojourn time** (the interval from tumor onset to its symptomatic detection)

These outputs are stratified by sex and race, providing insights into the epidemiology of bladder cancer in the simulated population.

References

1. National Comprehensive Cancer Network, others. NCCN clinical practice guidelines in oncology. http://www nccn org/professionals/physician_gls/PDF/occult pdf. 2008;

uOttawa

Reader's Guide
Model Purpose
Model Overview
Assumption Overview
Parameter Overview
Component Overview
Output Overview
Results Overview
Key References

Output Overview

Summary

This document describes the primary outputs of the COBRAS model and explains how they are defined and calculated.

Overview

COBRAS tracks each synthetic individual's life history, from birth until death for each birth-year cohort born between 1910 and 2020. In addition, the model can forecast bladder cancer outcomes through 2060 using U.S. Census projections. For those who develop bladder cancer, the model records the timing of tumor onset and detection. At the end of each simulation run, COBRAS produces a dataset representing the full natural history of bladder cancer for every simulated individual. By analyzing and summarizing these data, the model calculates its principal outputs.

Output Listing

The COBRAS model's main outputs include:

- **Age-specific incidence rate**
- **Stage-specific incidence rate**
- **Tumor proportion by stage**
- **Overall incidence**
- **Lifetime risk of developing bladder cancer**
- **Age of tumor onset**
- **Age of detection by symptoms**
- **Sojourn time**

All of these outputs can be stratified by age, sex, race, or smoking status to allow detailed analyses of subgroup differences.

Output definitions

Age-specific incidence rate

Let C_a denote the number of individuals newly diagnosed with bladder cancer in age group a, and N_a the number of individuals in that same age group who are alive. The age-specific incidence rate per 100,000 persons is:

$$CI_a = 100{,}000 \times \frac{C_a}{N_a}$$

Stage-specific incidence rates

Let C_s denote the number of individuals newly diagnosed at stage s, and let N represent the total living population. The incidence rate per 100,000 persons for stage s is:

$$CI_s = 100{,}000 \times \frac{C_s}{N}$$

Tumor proportion by stage

All material © Copyright 2025 CISNET

Let $ C $ be the total number of newly diagnosed cancer cases across all stages. The proportion of diagnoses occurring at stage $ s $ is:

$$P_s = \frac{C_s}{C}$$

Overall incidence

The overall incidence rate per 100,000 persons is:

$$CI = 100{,}000 \times \frac{C}{N}$$

Lifetime risk of developing bladder cancer

This metric reflects the proportion of individuals in the population who will eventually be diagnosed with bladder cancer. Let $ Surv_a $ be the proportion of the original cohort who survive to age $ a $. Then the lifetime risk of diagnosis is:

$$LR = \sum_{a=0}^{120} \left(Surv_a \times CI_a \right)$$

Here, $ CI_a $ is the incidence rate for age group $ a $.

Age of tumor onset

This output is the age at which tumors begin to develop in individuals who are ultimately diagnosed with bladder cancer. It focuses on the specific lesion that leads to symptomatic detection.

Age of detection by symptoms

This is the age at which bladder cancer is initially detected by symptoms during an individual's lifetime. If multiple tumors appear, only the one leading to symptomatic detection is counted in the model's records.

Sojourn time

Let $ Onset_i $ denote the tumor onset age and $ Detection_i $ the age of detection for individual $ i $. The sojourn time is:

$$Sojourn_i = Detection_i - Onset_i$$

This reflects the duration between the biological start of a tumor and the point of diagnosis through symptoms.

All material © Copyright 2025 CISNET

uOttawa

Reader's Guide
Model Purpose
Model Overview
Assumption Overview
Parameter Overview
Component Overview
Output Overview
Results Overview
Key References

Results Overview

Summary

This section describes the main results generated by the COBRAS model, including key outcomes from its Bayesian calibration process and the additional model-based metrics used to validate and interpret bladder cancer incidence patterns.

Overview

The results presented here reflect the current version of COBRAS, calibrated using the BayCANN approach (see Validation Overview for details). Future versions will expand on these findings, including long-term population trends and comparisons of various health strategies to reduce bladder cancer burden.

Results Listing

1. Metamodel Fit

The COBRAS model employs an ANN (artificial neural network) metamodel to approximate its more complex natural history processes. This ANN is trained to replicate age-specific and stage-specific incidences generated by the full simulation:

- **Figure 1** compares ANN-predicted vs. model-predicted age-specific incidences.
- **Figure 2** compares ANN-predicted vs. model-predicted stage-specific incidences.

These plots illustrate how well the metamodel approximates the outputs of the original simulation.

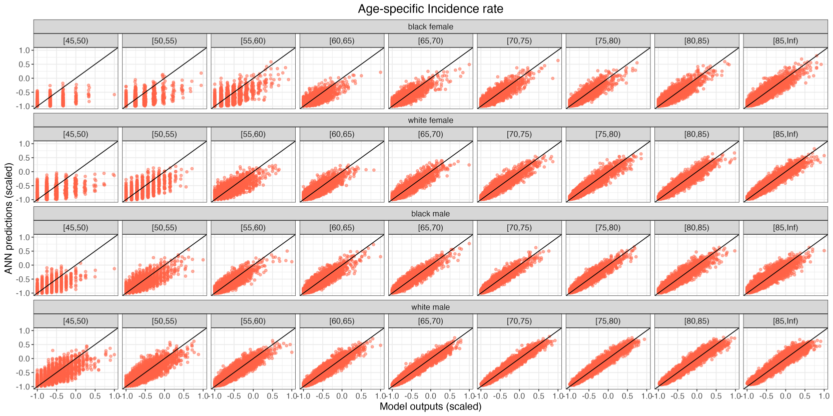

Figure 1: ANN predicted vs. model predicted age-specific incidences.

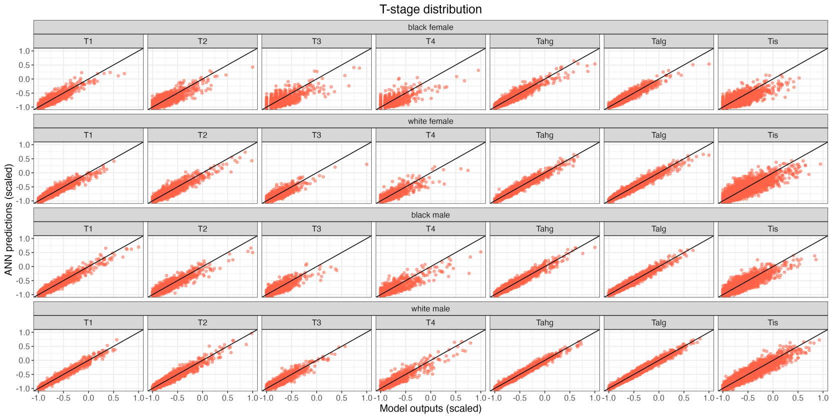

All material © Copyright 2025 CISNET

Figure 2: ANN predicted vs. model predicted stage-specific incidences.

2. Posterior Distributions of Calibrated Parameters

After training the ANN metamodel, COBRAS applies Bayesian calibration to estimate posterior distributions. The shift from prior assumptions to posterior estimates indicates how much the data inform each parameter:

Figure 3 shows prior vs. posterior distributions for the major calibrated parameters.

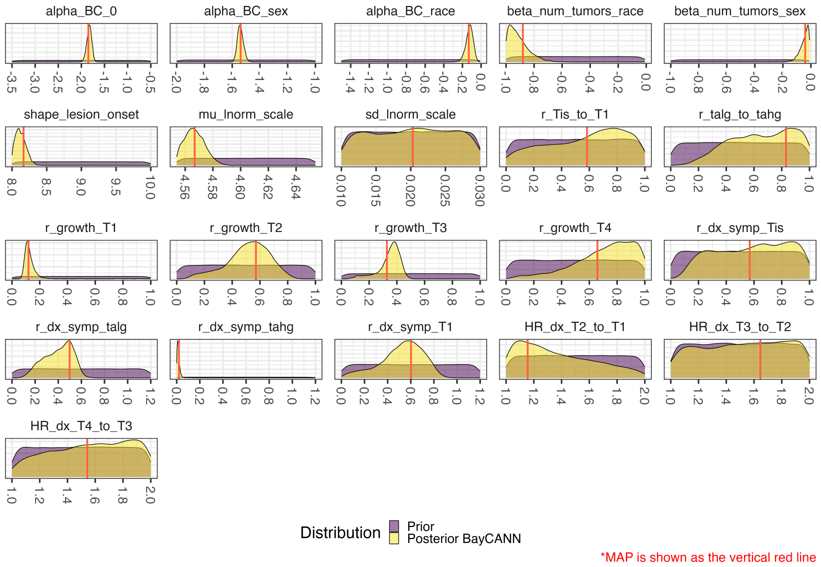

Figure 3: Prior and posterior distributions of calibrated parameters.

3. Model Validation

Using the calibrated parameter sets, COBRAS reruns the original natural history model and compares outputs with calibration targets:

- **Figure 4** presents age-specific incidence rates predicted by COBRAS relative to target data.
- **Figure 5** presents stage-specific incidence rates predicted by COBRAS relative to target data.

These comparisons help evaluate the accuracy of COBRAS in reproducing observed bladder cancer trends.

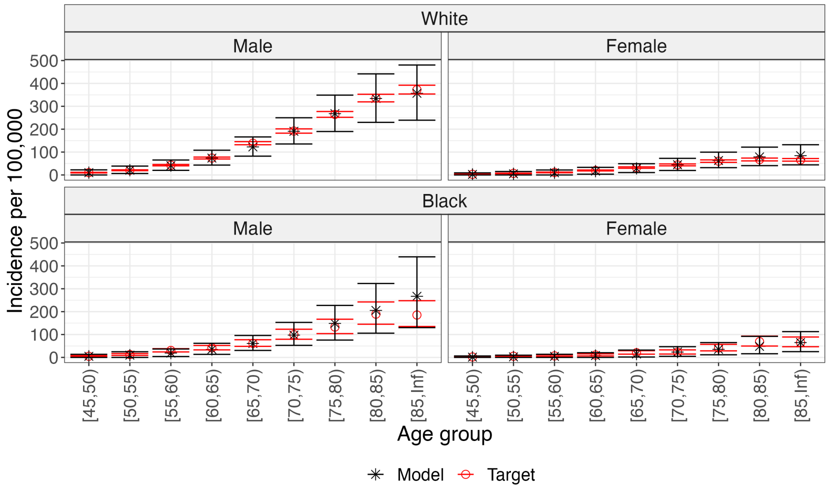

Figure 4: Age-specific incidence rates.

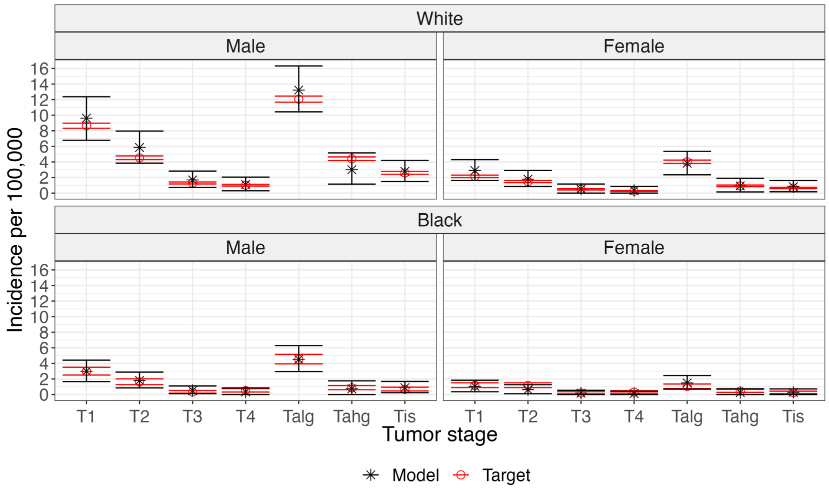

Figure 5: Stage-specific incidence rates.

4. Additional Model Outputs

Beyond the primary calibration targets, COBRAS generates a variety of supplementary results:

- **Tumor proportion by stage** (Figure 6)
- **Overall incidence** (Figure 7)
- **Lifetime risk of developing bladder cancer** (Figure 8)
- **Age of tumor onset** (Figure 9)
- **Age of symptom detection** (Figure 10)
- **Sojourn time** (Figure 11)

Each figure provides a deeper look into the natural history of bladder cancer in the simulated population.

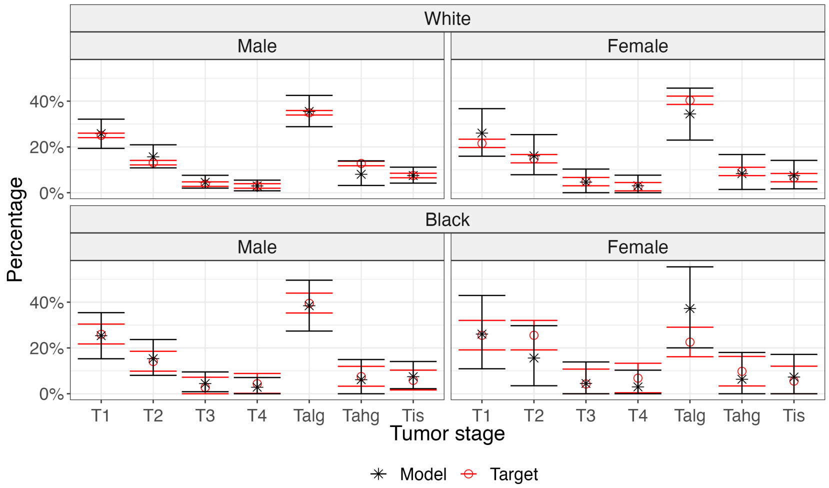

Figure 6: Tumor proportion by stage.

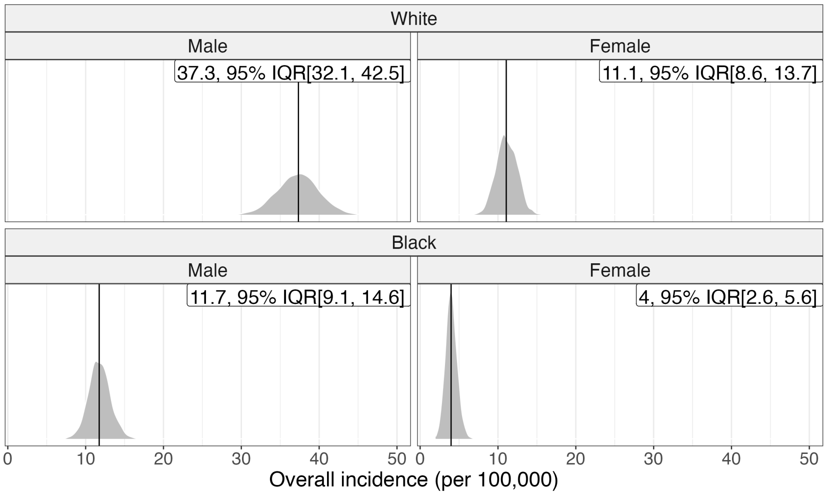

Figure 7: Overall incidence distribution.

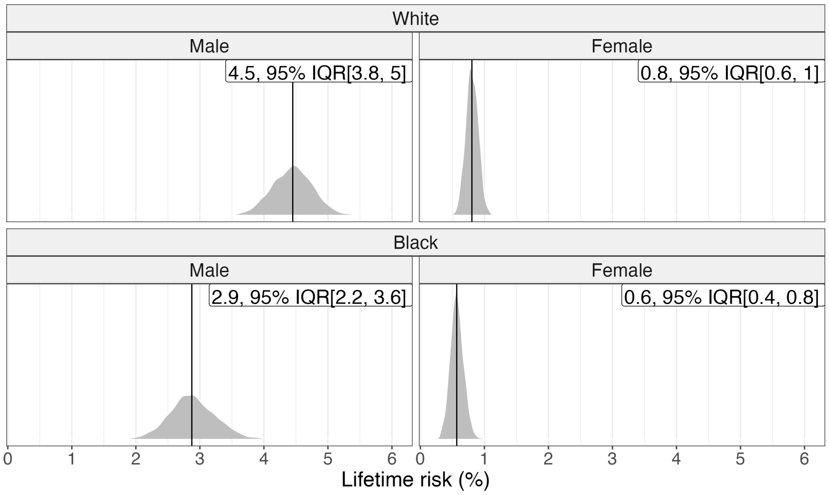

Figure 8: Lifetime risk of developing bladder cancer.

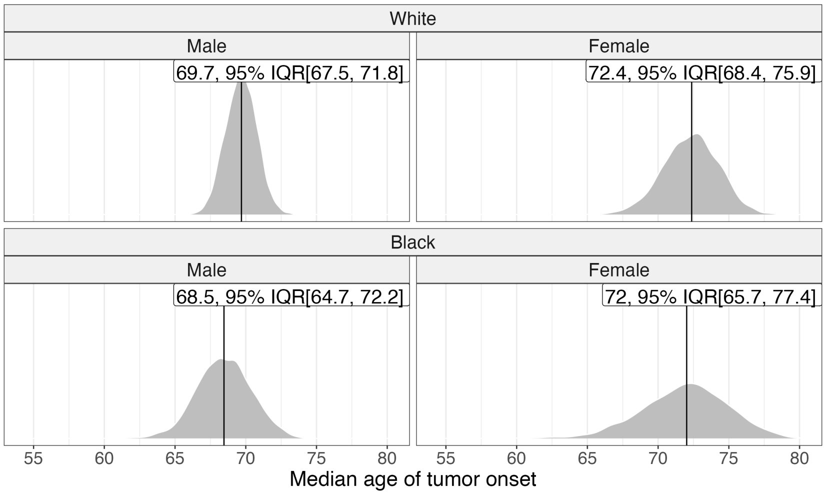

Figure 9: Age of tumor onset.

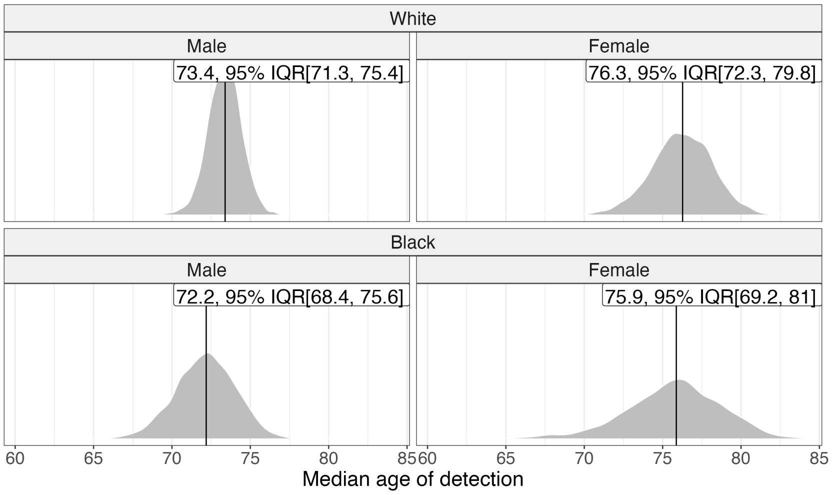

Figure 10: Age of symptom detection.

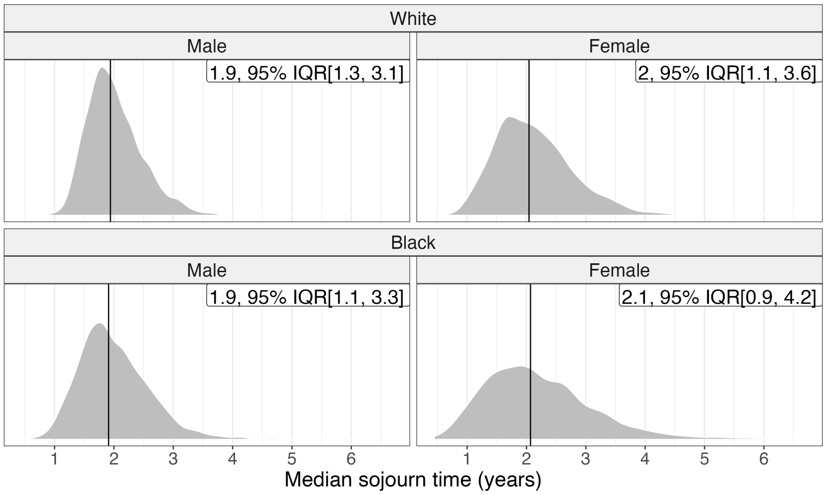

Figure 11: Median sojourn time.

uOttawa

Reader's Guide
Model Purpose
Model Overview
Assumption Overview
Parameter Overview
Component Overview
Output Overview
Results Overview
Key References

Validation Overview

Summary

This document summarizes the calibration and validation processes undertaken during the development of the COBRAS model.

Overview

COBRAS uses age- and stage-specific incidence data from 2010 Surveillance, Epidemiology, and End Results Program (SEER) statistics as primary calibration targets, stratified by sex and race for the U.S. population. To align model outputs with these targets, COBRAS employs a Bayesian Calibration using Neural Networks (BayCANN) methodology [1].

Target Definition

Age-specific incidence rates

Age-specific incidence rates for bladder cancer are computed by dividing the total number of cancer diagnoses in a given age group by the number of people alive in that age group (see the Output Overview for calculation details). COBRAS follows SEER's approach to categorizing age groups (0, 1–4, 5–9, …, 85+) but focuses on ages 45–49 and older for calibration. Rates are reported per 100,000 individuals, disaggregated by sex and race, with 95% confidence intervals constructed under a Poisson distribution assumption [2].

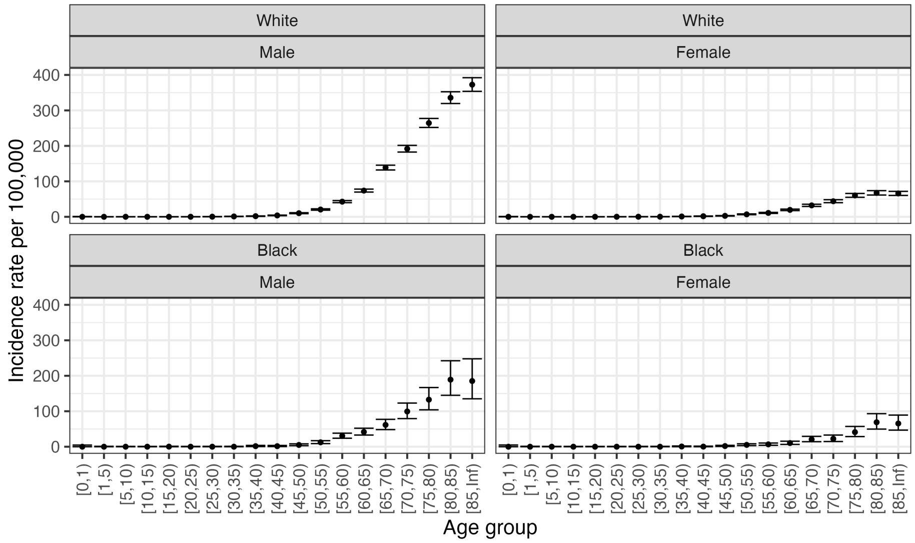

Figure 1: Age-specific incidence rate targets in the COBRAS model.

Stage-specific incidence rates (per 100,000)

Stage-specific incidence rates are computed by dividing the number of bladder cancer diagnoses at a particular stage by the number of people alive in the population. COBRAS considers the following stages: Tis, Ta–lg, Ta–hg, T1, T2, T3, and T4. These rates are stratified by sex, race, and smoking status, with 95% confidence intervals constructed similarly to the age-specific rates.

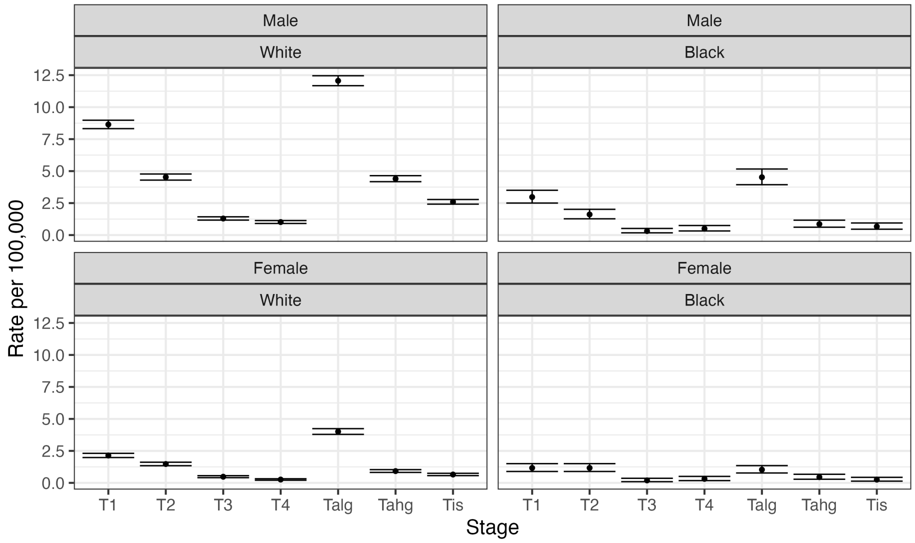

Figure 2: Stage-specific incidence rate targets in the COBRAS model.

Calibration Methodology

COBRAS relies on Bayesian Calibration using Artificial Neural Networks (BayCANN) [1] to align its outputs with target incidence data. A Bayesian approach estimates the full joint posterior distribution of parameters, capturing uncertainty more comprehensively. The BayCANN procedure consists of:

1. **Parameter Sampling**
   A set of plausible parameter values is generated using Latin Hypercube Sampling (LHS), defining lower and upper bounds for each calibrated parameter to ensure consistent sampling.

2. **Metamodel Training**
   An artificial neural network (ANN) is trained on the parameter sets and their corresponding model outputs, acting as a metamodel that can quickly approximate COBRAS output.

3. **Bayesian Calibration**
   A probabilistic programming environment is used to calibrate the ANN metamodel to observed data, treating the sampled parameters as an initial (prior) distribution.

4. **Posterior Distribution**
   Once the ANN is calibrated, it produces a posterior distribution for each parameter, which COBRAS can then use to generate updated outputs. These outputs are compared against the original targets to assess model fit.

All COBRAS parameters—calibrated or fixed—are listed in Parameter Overview, which also specifies those currently subject to calibration. The results of this calibration appear in the Results Overview.

References

1. Hawre Jalal, Thomas A Trikalinos, Fernando Alarid-Escudero. BayCANN: streamlining Bayesian calibration with artificial neural network metamodeling. Frontiers in Physiology. Frontiers Media SA; 2021;12:662314.
2. Michael P Fay. Approximate confidence intervals for rate ratios from directly standardized rates with sparse data. Communications in Statistics-Theory and Methods. Taylor & Francis; 1999;28(9):2141–2160.

uOttawa

[Reader's Guide](#)
[Model Purpose](#)
[Model Overview](#)
[Assumption Overview](#)
[Parameter Overview](#)
[Component Overview](#)
[Output Overview](#)
[Results Overview](#)
[Key References](#)

Key References

Elizabeth Arias, Jiaquan Xu, Kenneth Kochanek. United States life tables, 2021. 2023;

Michael P Fay. Approximate confidence intervals for rate ratios from directly standardized rates with sparse data. Communications in Statistics-Theory and Methods. Taylor & Francis; 1999;28(9):2141–2160.

David U Garibay-Treviño, Hawre Jalal, Fernando Alarid-Escudero. A Fast Nonparametric Sampling Method for Time to Event in Individual-Level Simulation Models. Medical Decision Making. SAGE Publications Sage CA: Los Angeles, CA; 2025;0272989X241308768.

Hawre Jalal, Thomas A Trikalinos, Fernando Alarid-Escudero. BayCANN: streamlining Bayesian calibration with artificial neural network metamodeling. Frontiers in Physiology. Frontiers Media SA; 2021;12:662314.

National Cancer Institute. Surveillance, Epidemiology, and End Results Program: Cancer Stat Facts, Bladder Cancer [Internet]. 2024. Available from: https://seer.cancer.gov/statfacts/html/urinb.html/

National Comprehensive Cancer Network, others. NCCN clinical practice guidelines in oncology. http://www nccn org/professionals/physician_gls/PDF/occult pdf. 2008;

R Core Team. R: A Language and Environment for Statistical Computing [Internet]. Vienna, Austria: R Foundation for Statistical Computing; 2025. Available from: https://www.R-project.org/
