# Supplementary material for: Bladder Cancer Burden in the USA: Population Scenarios for 2040
Source: Eur Urol Open Sci. 2025 Dec 4;83:99–108. doi: 10.1016/j.euros.2025.11.013 (PMC12721285; doi:10.1016/j.euros.2025.11.013)
Supplement: Supplementary Data 2 [file mmc2.pdf]

Kystis (Brown University)

BLADDER

Brown University
MPD Version:
1.0.00


Reader's Guide
Model Purpose
Model Overview
Assumption Overview
Parameter Overview
Component Overview
Output Overview
Results Overview
Key References


# Kystis

## Brown University

### Contact


Thomas A. Trikalinos (thomas_trikalinos@brown.edu)


### Suggested Citation

Forthcoming.

### Version Table

| Version | Release Date | Notes | Publications |
| --- | --- | --- | --- |
| 1.0.00 | 2025-02-27 | Initial version of the micro-simulation model (bladder cancer natural history). | N/A |

All material © Copyright 2025 CISNET

Kystis (Brown University)

BLADDER

Brown University
Readers Guide

BROWN


Reader's Guide

Model Purpose

Model Overview

Assumption Overview

Parameter Overview

Component Overview

Output Overview

Results Overview

Key References


# Reader's Guide

**Core Profile Documentation**

---

These topics will provide an overview of the model without the burden of detail. Each can be read in about 5-10 minutes. Each contains links to more detailed information if required.

**Model Purpose**
This document describes the primary purpose of the model.

**Model Overview**
This document describes the primary aims and general purposes of this modeling effort.

**Assumption Overview**
An overview of the basic assumptions inherent in this model.

**Parameter Overview**
Describes the basic parameter set used to inform the model, more detailed information is available for each specific parameter.

**Component Overview**
A description of the basic computational building blocks (components) of the model.

**Output Overview**
Definitons and methodologies for the basic model outputs.

**Results Overview**
A guide to the results obtained from the model.

**Key References**
A list of references used in the development of the model.

---

**Further Reading**

These topics will provide a intermediate level view of the model. Consider these documents if you are interested gaining in a working knowledge of the model, its inputs and outputs.

---

**Advanced Reading**

These topics denote more detailed documentation about specific and important aspects of the model structure

All material © Copyright 2025 CISNET

Reader's Guide
Model Purpose
Model Overview
Assumption Overview
Parameter Overview
Component Overview
Output Overview
Results Overview
Key References

# Model Purpose

## Summary

`Kystis` is a discrete event microsimulation model of bladder cancer in the U.S. population. It simulates individuals who, depending on their demographic characteristics and exposure history, may develop bladder lesions throughout their lives. The model simulates bladder cancer's natural history, including symptom development and disease progression, clinical detection, treatment, and mortality. It was developed to assess bladder cancer prevention, detection, and management strategies at both population and individual levels.

## Purpose

`Kystis` was developed to analyze bladder cancer trends and evaluate the impact of interventions, such as targeted screening, surveillance strategies for non-muscle invasive bladder cancer, and treatments for organ-confined bladder cancer.

`Kystis` aims to address the following questions:

- **Impact of carcinogens on past bladder cancer outcomes**. Smoking accounts for approximately 50% of all bladder cancer cases in men and up to 30% in women.[1-3]Despite decreases in smoking and various environmental exposure regulations, the incidence of bladder cancer has remained relatively stable over the past five decades, unlike the declining trends seen in other smoking-related cancers, such as lung cancer.[4,5] Our model aims to provide explanations for this discrepancy.
- **Population impact of widespread implementation of carcinogen control policies on bladder cancer prevention**. We will estimate the impact of existing policies that reduce smoking and/or other relevant carcinogens on the prevention of bladder cancer.
- **Effectiveness of bladder cancer screening among high-risk subgroups**. Routine bladder cancer screening is not recommended in the U.S. in part because of low disease incidence and the invasive nature of cystoscopy.[6] We will examine feasible screening strategies for high-risk subgroups, defined by combinations of screening modalities and schedules.
- **Effectiveness and cost-effectiveness of risk-based surveillance strategies for patients with non-muscle invasive bladder cancer (NMIBC)**. Although NMIBC is not life-threatening, it often recurs or progresses, requiring chronic surveillance. To identify optimal strategies, we will explore feasible surveillance policies, defined by combinations of testing modalities and schedules.
- **Comparative effectiveness of treatments for organ-confined bladder cancer**. We will compare (i) intravesical treatments for NMIBC, including bacille Calmette-Guerin (BCG) immunotherapy and chemotherapies; (ii) novel treatments such as gene therapies and antibody-drug conjugates for BCG-unresponsive NMIBC; and (iii) expanding bladder-sparing options, like immune checkpoint inhibitor-based immunotherapy, to novel bladder-preserving strategies and biomarker-driven chemotherapies for muscle-invasive bladder cancer (MIBC).

## References

1. Maximilian Burger, James WF Catto, Guido Dalbagni, H Barton Grossman, Harry Herr, Pierre Karakiewicz, et al. Epidemiology and Risk Factors of Urothelial Bladder Cancer. European Urology. Elsevier; 2013;63(2):234–241.
2. Marcus GK Cumberbatch, Ibrahim Jubber, Peter C Black, Francesco Esperto, Jonine D Figueroa, Ashish M Kamat, et al. Epidemiology of Bladder Cancer: A Systematic Review and Contemporary Update of Risk Factors in 2018. European Urology. Elsevier; 2018;74(6):784–795.
3. Neal D Freedman, Debra T Silverman, Albert R Hollenbeck, Arthur Schatzkin, Christian C Abnet. Association between smoking and risk of bladder cancer among men and women. Jama. American Medical Association; 2011;306(7):737–745.
4. NCI. SEER Cancer Stat Facts: Bladder Cancer 2019 [Internet]. 2019. Available from: https://seer.cancer.gov/statfacts/html/urinb.html

All material © Copyright 2025 CISNET 

5. Rebecca L Siegel, Kimberly D Miller, Hannah E Fuchs, Ahmedin Jemal. Cancer Statistics, 2022. CA: A Cancer Journal for Clinicians. 2022;72(1).
6. Virginia A Moyer. Screening for bladder cancer: US Preventive Services Task Force recommendation statement. Annals of internal medicine. American College of Physicians; 2011;155(4):246–251.

Reader's Guide
Model Purpose
Model Overview
Assumption Overview
Parameter Overview
Component Overview
Output Overview
Results Overview
Key References

# Model Overview

## Summary

This section provides an overview of the Kystis model structure and its components.

## Purpose

Kystis describes the natural history of bladder cancer and the impact of existing or emerging technologies for its prevention, control, and management. See Model Purpose for more details.

## Background

Bladder cancer is the sixth most common cancer in the U.S. and the fourth leading cause of cancer deaths in men, with over 83,700 new cases and 17,200 deaths annually.[1,2] The incidence of bladder cancer peaks after age 70 and is about three times higher in men than in women.[2-5] It is more commonly diagnosed in Non-Hispanic White persons compared to Non-Hispanic Black, Asian, or Hispanic persons.[2] However Non-Hispanic Black and Hispanic people are more likely to be diagnosed at a later stage and have worse outcomes.[6-8] On a per-patient basis, bladder cancer is the most expensive cancer to manage, surpassing colorectal, breast, prostate, and lung cancers.[9,10] Risk factors include environmental exposures such as cigarette smoking and chemical carcinogens found in the workplace or ingested, as well as genetic abnormalities.[11] Unlike other common cancers, the incidence and mortality rates of BC have remained relatively stable over the past fifty years.[1] The development of novel biomarkers and new treatments, including immunotherapies (checkpoint inhibitors), gene therapies, and antibody-drug conjugates, is expected to have a significant impact on bladder cancer epidemiology in the coming years.[12-14]

## Model Description

Kystis is a microsimulation model where events are simulated in continuous time. It can simulate the natural history of bladder cancer, exposures to environmental carcinogens (primarily smoking), and bladder cancer diagnosis, treatments, surveillance, and mortality in the U.S. population. The model simulates individuals with demographic attributes and bladder cancer risk factors, generate lesions and their trajectories.

Kystis models a series of events in parallel or sequentially (**Figure 1**). Hypothetical persons are instantiated with a sex and race category and a birthdate – typically the midpoint (June 30) of a calendar year. The model simulates mortality from other causes and exposure history in parallel from a person's birth. The lesion instantiation process begins when a person enters the simulation (spawn moment) and ends with a terminal event (death from bladder cancer or other causes). A person may develop zero, one, or several lesions. The risk of lesion development is a function of demographic attributes and exposure history. Once a lesion develops, it grows and progresses through a series of states, including death from bladder cancer as a terminal state. Lesion growth and transitions impact symptom development, which in turn affects the clinical detection of bladder cancer.

All material © Copyright 2025 CISNET

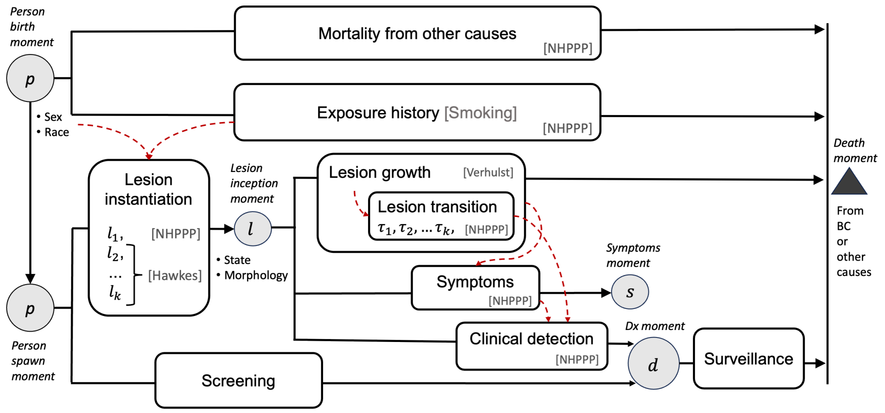


**Figure 1. Modeled processes in Kystis.**

# Implementation

`Kystis` is implemented in `R`. Users interface with a small set of classes that encapsulate all functionality:

- The `Population` class handles the simulation of the natural history of bladder cancer and the history of environmental exposures in a group of people. It includes methods to set up groups with user-defined demographics (birthdate, sex, race), and simulates their smoking and other exposure histories; initiates bladder cancer lesions and their evolution; and tracks symptoms and clinical diagnoses in individuals with bladder cancer.

- The `LesionSet` class handles the complete counterfactual history for the evolution of each lesion from its inception, to possibly, bladder cancer death.

- The states that bladder lesions can take and the allowed transitions between the lesion states are prescribed by the `StatesGraph` class. This object includes a graph of lesion states, with edges for the allowable transitions.

- An `RNGStreams` class instantiates independent random number streams that enables using Common Random Numbers or Antithetic Random Numbers for different parts of the simulation, using the L'Ecuyer-Lécot generator.[15,16] For example, there are different random number streams for smoking exposures, other toxin exposures, background mortality, lesion generation, lesion progression, testing strategies and treatment strategies.

- The `Simulator` class is responsible for setting up and running a simulation and, as needed, updating the simulation to run with alternative scenarios.

- The `Calibrator` class provides a convenient interface for handling calibration tasks. It generates calibration designs *de novo* or augments an existing design using various methods; updates the simulated population for (a subset of) the design, records results, and evaluates objective functions.

`Kystis` is implemented in `R` as a standalone package. The model is fully vectorized and some of its calulations are multi-threaded. A set of ancillary `R` packages abstract functionality that can be used by other models or more generally, and are imported into `Kystis`. Specifically,

- The `nhppp` package implements high performance algorithms written in `C` and `C++` for the vectorized simulation of event times from non-Homogeneous Poisson Point Processes. Simulating point processes is the computational workhorse with user-specified intensity and cumulative intensity functions.[17,18]

- The `mortality` package, which simulates death from any cause in the U.S. population based on historic data from various user-selectable sources.

- Exposure generators, currently represented by `smokingHxGen`, an extensively verified mathematically and numerically equivalent adaptation of the CISNET Lung Group Smoking History Generator version 5.2.1.[19]

Modules for bladder cancer screening, surveillance, and treatment are under development.

# References


1. NCI. SEER Cancer Stat Facts: Bladder Cancer 2019 [Internet]. 2019. Available from: https://seer.cancer.gov/statfacts/html/urinb.html
2. Rebecca L Siegel, Kimberly D Miller, Hannah E Fuchs, Ahmedin Jemal. Cancer Statistics, 2022. CA: A Cancer Journal for Clinicians. 2022;72(1).
3. Maximilian Burger, James WF Catto, Guido Dalbagni, H Barton Grossman, Harry Herr, Pierre Karakiewicz, et al. Epidemiology and Risk Factors of Urothelial Bladder Cancer. European Urology. Elsevier; 2013;63(2):234–241.
4. Marcus GK Cumberbatch, Ibrahim Jubber, Peter C Black, Francesco Esperto, Jonine D Figueroa, Ashish M Kamat, et al. Epidemiology of Bladder Cancer: A Systematic Review and Contemporary Update of Risk Factors in 2018. European Urology. Elsevier; 2018;74(6):784–795.
5. Jakub Dobruch, Siamak Daneshmand, Margit Fisch, Yair Lotan, Aidan P Noon, Matthew J Resnick, et al. Gender and Bladder Cancer: A Collaborative Review of Etiology, Biology, and Outcomes. European Urology. Elsevier; 2016;69(2):300–310.
6. Adam B Weiner, Mary-Kate Keeter, Adarsh Manjunath, Joshua J Meeks. Discrepancies in staging, treatment, and delays to treatment may explain disparities in bladder cancer outcomes: an update from the National Cancer Data Base (2004–2013). Urologic Oncology: Seminars and Original Investigations. Elsevier; 2018. p. 237-e9.
7. Yu Wang, Qian Chang, Yang Li. Racial differences in urinary bladder cancer in the United States. Scientific Reports. Nature Publishing Group UK London; 2018;8(1):12521.
8. Wesley Yip, Giovanni Cacciamani, Sumeet K Bhanvadia. Disparities in bladder cancer outcomes based on key sociodemographic characteristics. Current Urology Reports. Springer; 2020;21:1–6.
9. Elenir BC Avritscher, Catherine D Cooksley, H Barton Grossman, Anita L Sabichi, Lois Hamblin, Colin P Dinney, et al. Clinical Model of Lifetime Cost of Treating Bladder Cancer and Associated Complications. Urology. Elsevier; 2006;68(3):549–553.
10. Robert S Svatek, Brent K Hollenbeck, Sten Holmäng, Richard Lee, Simon P Kim, Arnulf Stenzl, et al. The Economics of Bladder Cancer: Costs and Considerations of Caring for This Disease. European Urology. Elsevier; 2014;66(2):253–262.
11. Marcus GK Cumberbatch, Aidan P Noon. Epidemiology, Aetiology and Screening of Bladder Cancer. Translational Andrology and Urology. AME Publications; 2019;8(1):5.
12. Dharmesh Gopalakrishnan, Vadim S Koshkin, Moshe C Ornstein, Athanasios Papatsoris, Petros Grivas. Immune Checkpoint Inhibitors in Urothelial Cancer: Recent Updates and Future Outlook. Therapeutics and Clinical Risk Management. Taylor & Francis; 2018;14:1019–1040.
13. Jonathan J Duplisea, Sharada Mokkapati, Devin Plote, Kimberly S Schluns, David J McConkey, Seppo Yla-Herttuala, et al. The development of interferon-based gene therapy for BCG unresponsive bladder cancer: from bench to bedside. World Journal of Urology. Springer; 2019;37:2041–2049.
14. Panagiotis J Vlachostergios, Christopher D Jakubowski, Muhammad J Niaz, Aileen Lee, Charlene Thomas, Amy L Hackett, et al. Antibody-drug conjugates in bladder cancer. Bladder Cancer. IOS Press; 2018;4(3):247–259.
15. Jack PC Kleijnen. Antithetic variates, common random numbers and optimal computer time allocation in simulation. Management Science. INFORMS; 1975;21(10):1176–1185.
16. Pierre L'Ecuyer, Christian Lécot, Bruno Tuffin. A randomized quasi-Monte Carlo simulation method for Markov chains. Operations research. INFORMS; 2008;56(4):958–975.
17. Thomas A Trikalinos, Yuliia Sereda. The nhppp package for simulating non-homogeneous Poisson point processes in R. PLoS One. Public Library of Science San Francisco, CA USA; 2024;19(11):e0311311.
18. Thomas A Trikalinos, Yuliia Sereda. nhppp: Simulating Nonhomogeneous Poisson Point Processes in R. arXiv preprint arXiv:240200358. 2024;


All material © Copyright 2025 CISNET 

19. Jihyoun Jeon, Rafael Meza, Martin Krapcho, Lauren D Clarke, Jeff Byrne, David T Levy. Chapter 5: Actual and counterfactual smoking prevalence rates in the US population via microsimulation. Risk Analysis: An International Journal. Wiley Online Library; 2012;32:S51–S68.

Kystis (Brown University)

BLADDER

Brown University
Assumption Overview

BROWN

Reader's Guide
Model Purpose
Model Overview
Assumption Overview
Parameter Overview
Component Overview
Output Overview
Results Overview
Key References

# Assumption Overview

## Summary

This section is an overview of assumptions for the `Kystis` natural history model.

---

## Background

`Kystis` simulates persons who have a urinary bladder. As persons age, and depending on their exposure histories and baseline risk, lesions may appear on the surface of the bladder. Lesions grow following one of four growth patterns that correspond to the WHO 2004 classification of urothelial and non-urothelial carcinomas. Lesions with different growth patterns have different growth curves, morphology, and invasive potential. Symptoms may appear depending on the lesions' size, location (e.g., involving the trigone of the bladder), and morphology.

---

## Assumption Listing

### Sampling times

We use Nonhomogeneous Poisson Point Processes (NHPPP) to sample event times. An NHPPP has the properties that the number of events in all non-overlapping time intervals are independent random variables and that, within each time interval, the number of events is Poisson distributed. `Kystis` model utilizes *nhppp* package to generate event times using the following algorithms: (i) time-transformation of a homogeneous Poisson process via the inverse of the integrated intensity function, and (ii) thinning of a majorizing NHPPP via an acceptance-rejection scheme.[1,2]

---

### Lesion risk

We model the occurrence of lesions using NHPPPs. Let $t$ measure time for the $n$-th person. We assume that the numbers of lesions in any finite set of non-overlapping time intervals are independent random variables, and that the number of lesions at any interval has a Poisson distribution. The instantaneous rate function of the process is a continuous positive function that is bounded on any finite interval.

$$\lambda_n(t) = \exp\left(\text{BASELINE}_n + \alpha_{\text{sex}}\text{sex}_n + \alpha_{\text{race}}\text{race}_n + \sum_{g \in \mathcal{G}_a} \alpha_{\text{age},g} \mathbb{1}(\text{age}_n(t) \in \text{age\_group}_g) + \text{SMOKING}_n(t) + \text{TOXINS}_n(t)\right)$$

Above, $\mathbb{1}(\cdot)$ is the indicator function, $\mathcal{G}_a$ is the set of covariates encoding age. The risk contribution $BASELINE_n(t)$ may include genetic information and it is currently limited to having Lynch Syndrome vs not. $SMOKING_n(t)$ encodes smoking status or intensity variables. $TOXINS_n(t)$ encodes environmental exposure histories, which are under development. These risk contributions have the following functional forms:

$$BASELINE_n = \alpha_{0n} + \alpha_{Lynch}\ Lynch_n \alpha_{0n} \sim N(\mu_{\alpha,baseline}, \sigma^2_{\alpha,baseline})$$
$$SMOKING_n(t) = \sum_{g \in \mathcal{G}_s} \alpha_{smoking,g}\ smoking_{n,g}(t)$$
$$TOXINS_n(t) = \sum_{g \in \mathcal{G}_x} \alpha_{toxins,g}\ toxins_{n,g}(t)$$

where, $\mu_{\alpha,baseline}, \sigma^2_{\alpha,baseline}$ are hyperparameters for the random intercept, and $\mathcal{G}_s$ and $\mathcal{G}_x$ are sets of covariates encoding smoking and environmental exposures, respectively.

The integrated rate of the process in the interval $(t_0, t]$ is the bounded non-decreasing function $\Lambda_n(t_0, t) = \int_{t_0}^{t} \lambda_n(s)\ d(s)$.

It is the expected number of lesions in $(t_0, t]$.

---

### Lesion growth

All material © Copyright 2025 CISNET

We use a Verhulst model to simulate lesion growth.[3] We assume that the rate of tumor growth (in terms of number of cells per year) is

$$N(t) = \frac{N_0 e^{rt}}{1+N_0(e^{rt}-1)/N_\infty},$$

where $N_0 = N(0)$ is the tumor's cell population at time 0, $r$ is a proportionality constant with units $year^{-1}$, and $N_\infty$ is the "carrying capacity" of the tumor, or the maximum number of cells that the tumor can attain (measured in cells). If the starting size $N_0$ and the maximum size $N_\infty$ of the lesion are fixed, the growth curve depends only on the parameter $r$.

The time $t_z$, measured in $days$, needed to reach a critical number of cells $N_z$ is obtained from

$$t_z = r^{-1}\left(\log\frac{N_\infty - N_0}{N_0} - \log\frac{N_\infty - N_z}{N_z}\right).$$

In the beginning the growth is almost exponential. The growth curve is convex until the inflection point at $N_\infty/2$ and then it becomes concave approaching asymptotically the carrying capacity $N_\infty$.

We make the following assumptions for the growth curve:

1. We instantiate lesions a size of $10^6$ cells, which corresponds to a volume of 1 $mm^3$.[4]
2. The carrying capacity for VLIP, HIP, NUC is assumed to be about $1.8 \cdot 10^{11}$ cells, corresponding to a spherical tumor of radius 3.5 $cm$.
3. The carrying capacity for CIS is assumed to be $4.57 \cdot 10^8$ cells. This corresponds to a CIS covering half of the bladder surface at a depth of 3 cellular layers.[5]
4. VLIP, HIP, and NUC lesions become visible when they are about 3 $mm$ in diameter, which corresponds to a size of $1.18 \cdot 10^8$ cells.
5. CIS lesions are assumed to become visible when they occupy an area of 0.45 $cm^2$ (roughly 7-8 $mm$ circumscribed circle diameter), which is the average size of a tile. This corresponds to approximately $1.35 \cdot 10^6$ cells - so CIS becomes visible soon after it appears.
6. Lesions become visible after $4.60616/r_c$ years for $c \in \{\text{VLIP}, \text{HIP}, \text{NUC}\}$ and after $0.30014/r_{\text{CIS}}$ years for CIS.
7. **An average growth rate for bladder cancer lesions has been estimated at $r_c \approx 0.043\ day^{-1}$ for all $c$.[6] This would suggest that from inception, non-CIS lesions would become visible within approximately 107 days (3.6 months), but CIS lesions would be visible at inception (more accurately, after about 7 days).**

## Lesion starting states and transitions

**Figure 1** shows the modeled transitions from lesion inception to death from bladder cancer. Lesions first appear in states 0, 1, 2, 4, 6, or 8. Arrows represent allowable transitions that follow a NHPPP law. PUMLNPs (state 0) do not commonly advance to Ta lesions.[5] VLIP lesions (state 1) do not transition to other states. However they can grow in size and give symptoms like all other lesions.

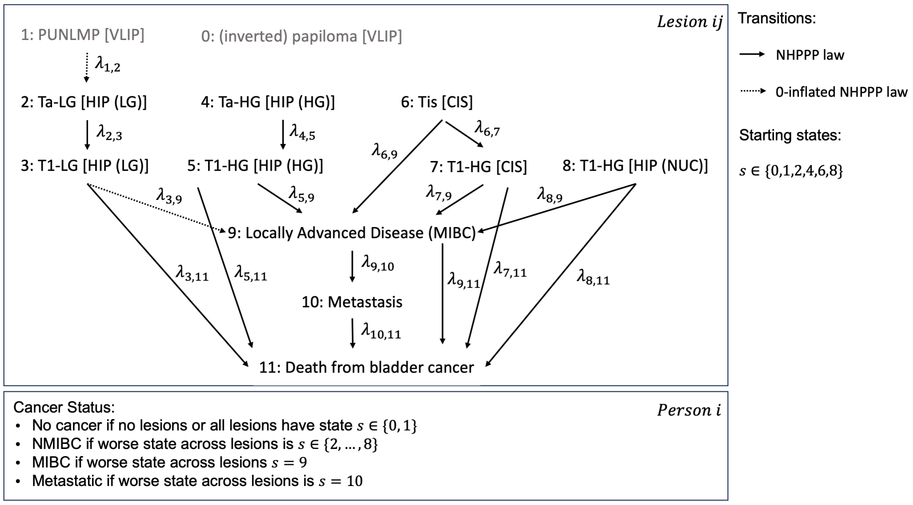

**Figure 1. Transitions between states for lesions in *Kystis* and designation of cancer status at the person level.**

We assume that

- HIP (HG) is more aggressive than HIP (LG) of the same size, so that $\zeta_{4,5} > \zeta_{2,3}$ and $\zeta_{5,9} \geq \zeta_{3,9}$.
- We assume that NUC is at least as aggressive as HIP (HG) of the same size, so that $\zeta_{8,9} \geq \zeta_{4,5}$ and $\zeta_{8,9} \geq \zeta_{5,9}$
- CIS lesions are flat and their tumor cell size tends to be smaller that that of other growth patterns. For example, for CIS the carrying capacity is $N_\infty \approx 4.57 \cdot 10^8$ cells, where as for other growth patterns $N_\infty \approx 1.8 \cdot 10^{11}$ cells. Thus the $\zeta$'s for CIS are not explicitly constrained with respect to those of the other growth patterns and are set through calibration.

The instantaneous rates for transitions are a function of the size of the lesion.
$\lambda_{ij}(t) = \zeta_{ij} \log(N(t)),\ \text{with } \zeta_{ij} > 0$

Transitions from PUMLNP to Ta-LG ($1 \rightarrow 2$) are considered uncommon.[5] Analogously, transitions from low grade tumors to Advanced disease may be uncommon or rare. Such uncommon or rare transitions are modelled with zero-inflated NHPPP.
$z \sim \mathit{Bernoulli}(\Pr[i \rightarrow j])$,
$\lambda_{ij}(t) = z\, \zeta_{ij}\ \log\Big(N(t)\Big)$, with $\zeta_{ij} > 0$ ,

where the probability that a transition will occur $\Pr[i \rightarrow j]$ is obtained via calibration.

---

## Symptom onset

We make the following assumptions:

- The majority of patients who present with symptoms have gross hematuria (80%); we assume that the rest report primarily symptoms during micturition.[5]
- A person becomes symptomatic when they develop either hematuria or symptoms during micturition.
- The probability of hematuria is related to the size of individual lesions.
- The probability of symptoms during micturition is related to
    1. the fraction of the total surface area of the bladder that is occupied by at least one lesion.
    2. the fraction of the surface area of the trigone that is occupied by at least one lesion
- We assume that we can model onset of symptoms and clinical detection using NHPPP.

### Onset of voiding symptoms

The time that the $n$-th person will experience voiding symptoms is measured on a person clock starting from the instantiation of the first lesion ($t > t_{l1}$).

We assume that the time to symptom onset is modeled with a NHPPP with instantaneous rate
$\eta_m(t) = \exp(\delta_{m0} + \delta_{m1}\, S^{-1}_{bladder} \sum_{j \in \mathcal{J}_t} S_j(t) + \delta_{m2}\, S^{-1}_{trigone} \sum_{j \in \mathcal{J}_t} S_{trig,j}(t))$ .

Above, $j$ indexes lesions in $\mathcal{J}_t$, $\mathcal{J}_t$ is the set of lesions that are present at time $t$, and involve at least $S_{min} = 1\ cm^2$ on the surface of the bladder, $S_j(t)$ is the area of the bladder's surface that is occupied by lesion $j$, and $S_{trig,j}(t)$ is the area of the trigone that is occupied by lesion $j$. The coefficients $\delta_{m1} > 0$ and $\delta_{m2} > 0$ determine the association between symptoms during micturition and the proportion of the surface of the bladder of the trigone that is occupied by a lesion, and $\delta_{m0}$ is an intercept term.
To facilitate calibration, we assume that $\delta_1 = \delta_2$ are global constants.

### Onset of macroscopic hematuria

For the $j$-thlesion of the $n$-th person we model the onset of macroscopic hematuria, as measured on the lesion clock, as the first event from a NHPPP with instantaneous rate
$\eta_h(t) = \exp(\delta_{h0} + \delta_h \cdot \log\left(N_j(t)\right))$ ,

where the coefficient $\delta_h > 0$ determines the association between symptoms of hematuria and the size of the lesion. To facilitate calibration, we assume that the intercept $\delta_{h0}$ and $\delta_h$ are fixed parameters.

## Clinical detection

The probability of clinical detection by time $t$, measured on the person clock starting from the instantiation of the first lesion $t_{l1}$. It is modelled as the time to the first event in a NHPPP with instantaneous rate $\eta_s(t) = \exp\left(\delta_{s1} + \delta_{s2}\mathbb{1}(sex = \text{Female}) + \delta_{s3}\mathbb{1}(race = \text{Black}) + \delta_{s4}(t - t_{\text{micturition}})_+ + \delta_{s5}(t - t_{\text{hematuria}})_+\right)$ ,

where $t$ is measured on the person clock, $(t - t_{\text{micturition}})_+$, is the time after the onset of voiding symptoms and zero otherwise, and $(t - t_{\text{hematuria}})_+$ the time after the earliest onset of hematuria symptoms and 0 otherwise. We assume that delays in diagnosis are in part a misattribution of symptoms to urinary infections (for women) or barriers to seeking care (in Black individuals).[7]

## Sojourn time

The sojourn time is the length of the time interval between the moment that a malignant lesion $j$ becomes detectable by cystoscopy, $t_{cystoscopy\_detectable,j}$, and the time that a person is symptomatic and has clinically detectable cancer, $t_{clinical\_detection}$. It is defined for lesions that have muscle-invasive potential. It is not modeled explicitly, but it is calculated based on other quantities.

## Time to death

We assume that a person can die from causes related to bladder cancer and other causes. We model time to each cause of death with competing NHPPP. The earliest time of death (due to bladder cancer or other causes) is the one that is realized. Another assumption is that death from other causes than bladder cancer is approximately equal to that of all causes in the general population stratified by age, sex, race, and smoking history.

When modeling time to death from bladder cancer, we assume that: (i) patients with NMIBC or earlier stages of the disease have a very low probability of dying from bladder cancer; (ii) patients die of bladder cancer if they have MIBC or metastatic disease; and (iii) patients with metastatic disease experience shorter bladder cancer survival rates.

# References

1. Thomas A Trikalinos, Yuliia Sereda. The nhppp package for simulating non-homogeneous Poisson point processes in R. PLoS One. Public Library of Science San Francisco, CA USA; 2024;19(11):e0311311.
2. Thomas A Trikalinos, Yuliia Sereda. nhppp: Simulating Nonhomogeneous Poisson Point Processes in R. arXiv preprint arXiv:240200358. 2024;
3. Nicolas Bacaër. Verhulst and the logistic equation (1838). A short history of mathematical population dynamics. London: Springer London; 2011. p. 35–39.
4. John A Spratt, D Von Fournier, John S Spratt, Ernst E Weber. Decelerating growth and human breast cancer. Cancer. Wiley Online Library; 1993;71(6):2013–2019.
5. Petrisor Aurelian Geavlete. Endoscopic Diagnosis and Treatment in Urinary Bladder Pathology: Handbook of Endourology. Academic Press; 2016.
6. Cyrill A Rentsch, Claire Biot, Joël R Gsponer, Alexander Bachmann, Matthew L Albert, Romulus Breban. BCG-Mediated Bladder Cancer Immunotherapy: Identifying Determinants of Treatment Response Using a Calibrated Mathematical Model. PloS one. 2013 Feb;8(2):e56327-17.
7. NCI. SEER Cancer Stat Facts: Bladder Cancer 2019 [Internet]. 2019. Available from: https://seer.cancer.gov/statfacts/html/urinb.html

All material © Copyright 2025 CISNET 

Reader's Guide
Model Purpose
Model Overview
Assumption Overview
Parameter Overview
Component Overview
Output Overview
Results Overview
Key References

# Parameter Overview

## Summary

Describes the basic parameter set used to inform the model, more detailed information is available for each specific parameter.

---

## Background

The `Kystis` model uses two types of parameters:

- **Fixed parameters**: These include global constants such as the maximum age of a person or baseline specifications of the urinary bladder. These parameters are detailed in the Component Overview.
- **Calibrated parameters** These parameters are used to define and adjust model processes, such as how lesions start and progress. They are fine-tuned to ensure the model's output closely matches observed data. This page focuses on calibrated parameters.

For more information on the equations used, refer to the Assumption Overview.

---

## Parameter Listing Overview

**Table 1** summarizes the parameters used in the Kystis model to simulate bladder cancer onset, lesion growth and transitions, symptom development, and clinical detection.

- *Lesion instantiation* rate depends on factors such as sex, race, age, and exposure history. Exposure history is limited to smoking in the current version of the model and accounts for both smoking status and smoking intensity. The age is modeled as a continuous covariate with additional risk after specific age thresholds (i.e., greater than {50,55,60,65,70,75,80,85}). The risk is capped at older ages ( `truncation_age`) to match the plateau observed in age-specific bladder cancer incidence curves. Lesion instantiation parameters vary by the tumor grade (low or high grade).
- *Lesion growth* is a Verhulst model, which is based on a time-varying tumor growth rate measured in the number of cells per day. Two additional parameters (`growth_mult_VLIP` and `growth_mult_HIP_LG`) scale growth curves for lesions with VLIP and HIP growth patterns.
- *Voiding symptoms* rate is a function of lesion spread on the urinary bladder surface. Lesions located at the bladder trigone are assumed to increase the likelihood of voiding symptoms.
- *Macroscopic bleeding* is modeled as a function of lesion size measured in cells.
- *Bladder cancer clinical detection* depends on the time since symptom onset (if symptoms are present), sex, race, and the development of advanced disease (e.g., MIBS or metastasis).
- *Lesion transitions* are modeled using a graph consisting of vertices (nodes) and edges connecting pairs of vertices. Vertex parameters include the names of allowed states, starting state probabilities, and morphology probabilities. There are 16 allowed edges in the graph, each as a function of lesion size. Two edges ('PUNLMP [VLIP]' to 'Ta-LG [HIP LG]' and 'T1-LG [HIP LG]' to 'Locally advanced disease') are considered uncommon and are modeled with zero-inflated NHPPP, using zero mass probability as an additional parameter.

**Table 1. `Kystis` calibrated parameters.**

**A. Lesion instantiation.**

| Parameter | Hyperparameters | Description |
|---|---|---|
| `intercept` | $N(\mu, \sigma^2)$ | Log risk of bladder cancer among unexposed White Men at birth |
| `lynch_syndrom` | $N(\mu, \sigma^2)$ | Change in log risk of bladder cancer for Lynch syndrome vs. not |
| `sex_female` | $N(\mu, \sigma^2)$ | Change in log risk of bladder cancer for Female vs. Male biological sex |

All material © Copyright 2025 CISNET

| Parameter | Hyperparameters | Description |
| --- | --- | --- |
| `race_black` | $N(\mu, \sigma^2)$ | Change in log risk of bladder cancer for Black vs. White Race |
| `age` | $U(a, b)$ | Change in log risk of bladder cancer per unit [year] increase in age, for ages below `truncation_age` |
| `age_ge_X` | $N(\mu, \sigma^2)$ | If age is ≥ X years, these terms add a constant value to the log risk of bladder cancer |
| `truncation_age` | $U(a, b)$ | Age beyond which bladder cancer risk is capped |
| `past_smoker` | $N(\mu, \sigma^2)$ | Change in log risk of bladder cancer for former smokers vs. not |
| `smoking_exposure` | $N(\mu, \sigma^2)$ | Change in log risk of bladder cancer per unit increase in smoking cumulative intensity |
| `toxin_exposure` | $N(\mu, \sigma^2)$ | Change in log risk of bladder cancer per unit increase in toxin cumulative intensity |

### B. Lesion growth.

| Parameter | Hyperparameters | Description |
| --- | --- | --- |
| `growth_rate` | $U(a, b)$ | Rate of tumor growth, number of cells per day |
| `growth_mult_VLIP` | $U(a, b)$ | Multiplyer for the rate of tumor growth in lesions with VLIP growth pattern |
| `growth_mult_HIP_LG` | $U(a, b)$ | Multiplyer for the rate of tumor growth in low-grade lesions with HIP growth pattern |
| `log_N0` | $U(a, b)$ | Log of tumor's cell population at time 0 |
| `N0_to_Ninf_ratio_nonCIS` | $U(a, b)$ | Ratio of tumor's cell population at time 0 and the maximum number of cells that the tumor can attain (measured in cells) in lesions with VLIP, HIP or NUC growth pattern |
| `N0_to_Ninf_ratio_CIS` | $U(a, b)$ | Ratio of tumor's cell population at time 0 and the maximum number of cells that the tumor can attain (measured in cells) in lesions with CIS growth pattern |

### C. Voiding symptoms.

| Parameter | Hyperparameters | Description |
| --- | --- | --- |
| `intercept` | $U(a, b)$ | Risk of voiding symptoms at lesion instantiation |
| `S_bladder_fraction` | $U(a, b)$ | Risk of voiding symptoms per unit change in the area of the bladder's surface that is occupied by lesion |
| `S_trigone_fraction` | $U(a, b)$ | Risk of voiding symptoms per unit change in the area of the bladder's trigone that is occupied by lesion |

### D. Macroscopic bleeding.

| Parameter | Hyperparameters | Description |
| --- | --- | --- |
| `intercept` | $U(a, b)$ | Risk of macroscopic bleeding at lesion instantiation |
| `logN` | $U(a, b)$ | Risk of macroscopic bleeding per unit change in the log of tumor's cell population |

### E. Clinical detection.

| Parameter | Hyperparameters | Description |
| --- | --- | --- |
| `intercept` | $U(a, b)$ | Risk of clinical detection of NMIBC in White Males without symptoms |
| `sex_female` | $U(a, b)$ | Change in risk of clinical detection for Female vs. Male biological sex |
| `race_black` | $U(a, b)$ | Change in risk of clinical detection for Black vs. White Race |
| `macroscopic_bleeding` | $N(\mu, \sigma^2)$ | Change in risk of clinical detection per unit increase in time since macroscopic bleeding started or zero otherwise |
| `voiding_symptoms` | $N(\mu, \sigma^2)$ | Change in risk of clinical detection per unit increase in time since voiding symptoms started or zero otherwise |
| `mibc` | $U(a, b)$ | Change in risk of clinical detection for developing MIBC vs. NMIBC or precancerous states |
| `metastasis` | $U(a, b)$ | Change in risk of clinical detection for developing metastatic disease vs. NMIBC or precancerous states |

### F. Lesion graph vertices.

| Parameter | Hyperparameters | Description |
| --- | --- | --- |
| `prop_LG` | $U(a, b)$ | Proportion of low grade lesions at lesion instantiation |

*All material © Copyright 2025 CISNET*

| Parameter | Hyperparameters | Description |
|---|---|---|
| `state_names` | {'Papilloma [VLIP]', 'PUNLMP [VLIP]', 'Ta-LG [HIP LG]', 'T1-LG [HIP LG]', 'Ta-HG [HIP HG]', 'T1-HG [HIP HG]', 'Tis [CIS]', 'T1-HG [CIS]', 'T1-HG [NUC]', 'Locally advanced disease', 'Metastasis', 'BC death'} | Allowed lesion states |
| `starting_state_probs` | {0, 0, 0.675, 0, 0.225, 0, 0.09, 0, 0.01, 0, 0, 0} | Probabilities of lesion states at onset, in the order of `state_names` |
| `flat_morphology_probs` | {0, 0, 0, 0, 0, 0, 1, 0, 0, 0, 0, 0} | Probabilities of flat morphology at lesion onset, in the order of `state_names` |
| `stalked_morphology_probs` | {1, 1, 0.8, 0.8, 0.2, 0.2, 0, 0.2, 0, 0, 0, 0} | Probabilities of stalked morphology at lesion onset, in the order of `state_names` |

### G. Lesion graph edges.

| Parameter | Hyperparameters | Description |
|---|---|---|
| `punlmp__to__talg_logN` | $U(a, b)$ | Change in risk of lesion transition from 'PUNLMP [VLIP]' to 'Ta-LG [HIP LG]' per unit increase in log of lesion size measured in cells |
| `talg__to__t1lg_logN` | $U(a, b)$ | Change in risk of lesion transition from 'Ta-LG [HIP LG]' to 'T1-LG [HIP LG]' per unit increase in log of lesion size measured in cells |
| `t1lg__to__lad_logN` | $U(a, b)$ | Change in risk of lesion transition from 'T1-LG [HIP LG]' to 'Locally advanced disease' per unit increase in log of lesion size measured in cells |
| `t1lg__to__bcdeath_logN` | $U(a, b)$ | Change in risk of lesion transition from 'T1-LG [HIP LG]' to 'BC death' per unit increase in log of lesion size measured in cells |
| `tahg__to__t1hg_logN` | $U(a, b)$ | Change in risk of lesion transition from 'Ta-HG [HIP HG]' to 'T1-HG [HIP HG]' per unit increase in log of lesion size measured in cells |
| `t1hg__to__lad_logN` | $U(a, b)$ | Change in risk of lesion transition from 'T1-HG [HIP HG]' to 'Locally advanced disease' per unit increase in log of lesion size measured in cells |
| `t1hg__to__bcdeath_logN` | $U(a, b)$ | Change in risk of lesion transition from 'T1-HG [HIP HG]' to 'BC death' per unit increase in log of lesion size measured in cells |
| `tis__to__t1cis_logN` | $U(a, b)$ | Change in risk of lesion transition from 'Tis [CIS]' to 'T1-HG [CIS]' per unit increase in log of lesion size measured in cells |
| `tis__to__lad_logN` | $U(a, b)$ | Change in risk of lesion transition from 'Tis [CIS]' to 'Locally advanced disease' per unit increase in log of lesion size measured in cells |
| `t1cis__to__lad_logN` | $U(a, b)$ | Change in risk of lesion transition from 'T1-HG [CIS]' to 'Locally advanced disease' per unit increase in log of lesion size measured in cells |
| `t1cis__to__bcdeath_logN` | $U(a, b)$ | Change in risk of lesion transition from 'T1-HG [CIS]' to 'BC death' per unit increase in log of lesion size measured in cells |
| `t1nuc__to__lad_logN` | $U(a, b)$ | Change in risk of lesion transition from 'T1-HG [NUC]' to 'Locally advanced disease' per unit increase in log of lesion size measured in cells |
| `t1nuc__to__bcdeath_logN` | $U(a, b)$ | Change in risk of lesion transition from 'T1-HG [NUC]' to 'BC death' per unit increase in log of lesion size measured in cells |
| `lad__to__meta_logN` | $U(a, b)$ | Change in risk of lesion transition from 'Locally advanced disease' to 'Metastasis' per unit increase in log of lesion size measured in cells |
| `lad__to__bcdeath_logN` | $U(a, b)$ | Change in risk of lesion transition from 'Locally advanced disease' to 'BC death' per unit increase in log of lesion size measured in cells |
| `meta__to__bcdeath_logN` | $U(a, b)$ | Change in risk of lesion transition from 'Metastasis' to 'BC death' per unit increase in log of lesion size measured in cells |
| `punlmp__to__talg_pr0mass` | $U(a, b)$ | Probability of observing no transitions from 'PUNLMP [VLIP]' to 'Ta-LG [HIP LG]' in a given interval in zero-inflated NHPPP |
| `talg__to__t1lg_pr0mass` | $U(a, b)$ | Probability of observing no transitions from 'Ta-LG [HIP LG]' to 'T1-LG [HIP LG]' in a given interval in zero-inflated NHPPP |
| `t1lg__to__lad_pr0mass` | $U(a, b)$ | Probability of observing no transitions from 'T1-LG [HIP LG]' to 'Locally advanced disease' in a given interval in zero-inflated NHPPP |
| `t1lg__to__bcdeath_pr0mass` | $U(a, b)$ | Probability of observing no transitions from 'T1-LG [HIP LG]' to 'BC death' in a given interval in zero-inflated NHPPP |
| `tahg__to__t1hg_pr0mass` | $U(a, b)$ | Probability of observing no transitions from 'Ta-HG [HIP HG]' to 'T1-HG [HIP HG]' in a given interval in zero-inflated NHPPP |
| `t1hg__to__lad_pr0mass` | $U(a, b)$ | Probability of observing no transitions from 'T1-HG [HIP HG]' to 'Locally advanced disease' in a given interval in zero-inflated NHPPP |
| `t1hg__to__bcdeath_pr0mass` | $U(a, b)$ | Probability of observing no transitions from 'T1-HG [HIP HG]' to 'BC death' in a given interval in zero-inflated NHPPP |
| `tis__to__t1cis_pr0mass` | $U(a, b)$ | Probability of observing no transitions from 'Tis [CIS]' to 'T1-HG [CIS]' in a given interval in zero-inflated NHPPP |

All material © Copyright 2025 CISNET 

| Parameter | Hyperparameters | Description |
| --- | --- | --- |
| `tis__to__lad_pr0mass` | $U(a, b)$ | Probability of observing no transitions from 'Tis [CIS]' to 'Locally advanced disease' in a given interval in zero-inflated NHPPP |
| `t1cis__to__lad_pr0mass` | $U(a, b)$ | Probability of observing no transitions from 'T1-HG [CIS]' to 'Locally advanced disease' in a given interval in zero-inflated NHPPP |
| `t1cis__to__bcdeath_pr0mass` | $U(a, b)$ | Probability of observing no transitions from 'T1-HG [CIS]' to 'BC death' in a given interval in zero-inflated NHPPP |
| `t1nuc__to__lad_pr0mass` | $U(a, b)$ | Probability of observing no transitions from 'T1-HG [NUC]' to 'Locally advanced disease' in a given interval in zero-inflated NHPPP |
| `t1nuc__to__bcdeath_pr0mass` | $U(a, b)$ | Probability of observing no transitions from 'T1-HG [NUC]' to 'BC death' in a given interval in zero-inflated NHPPP |
| `lad__to__meta_pr0mass` | $U(a, b)$ | Probability of observing no transitions from 'Locally advanced disease' to 'Metastasis' in a given interval in zero-inflated NHPPP |
| `lad__to__bcdeath_pr0mass` | $U(a, b)$ | Probability of observing no transitions from 'Locally advanced disease' to 'BC death' in a given interval in zero-inflated NHPPP |
| `meta__to__bcdeath_pr0mass` | $U(a, b)$ | Probability of observing no transitions from 'Metastasis' to 'BC death' in a given interval in zero-inflated NHPPP |

*All material © Copyright 2025 CISNET*

Reader's Guide
Model Purpose
Model Overview
Assumption Overview
Parameter Overview
Component Overview
Output Overview
Results Overview
Key References

# Component Overview

## Summary

A description of the basic computational building blocks (components) of the model.

## Overview

`Kystis` models the natural history of bladder cancer through a combination of parallel and sequential processes. It generates a population where each person has specific demographic attributes. From birth, the model simulates mortality from causes other than bladder cancer and tracks exposure history. Individuals can develop zero, one, or multiple lesions. The process of lesion formation begins when a person enters the simulation (spawn moment) and is influenced by their demographic attributes and exposure history. Once a lesion instantiated, it grows and progresses through several stages and can ultimately result in death from bladder cancer. Lesion growth and transitions impact symptom development, which in turn affects the clinical detection of bladder cancer.

## Component Listing

### Time clocks

We use time clock objects to count time unambiguously. These objects have an internal representation that is concordant with ISO 8601.[1] They have a common origin date, are aware of time units, and can be aligned with each other.

In the simulation, we measure time on *calendar*, *person* and *lesion* clocks, where for the $n$-th person and $j$-th lesion:

$$\texttt{calendar\_clock(n)}.years() = \texttt{person\_clock(n)}.years() + birth\_cohort_n + sim\_start\_age_n$$
$$= \texttt{lesion\_clock(n, j)}.years() + birth\_cohort_n + lesion\_start\_age_{nj}$$
$$\texttt{person\_clock(n)}.years() = \texttt{calendar\_clock(n)}.years() - birth\_cohort_n - sim\_start\_age_n$$
$$= \texttt{lesion\_clock(n, j)}.years() + lesion\_start\_age_{nj} - sim\_start\_age_n, \text{ and}$$
$$\texttt{lesion\_clock(n, j)}.years()$$
$$= \texttt{calendar\_clock()}.years() - birth\_cohort_n - lesion\_start\_age_{nj}$$
$$= \texttt{person\_clock(n)}.years() - lesion\_start\_age_{nj} + sim\_start\_age_n$$

### Person

When instantiated, each simulated person has or is associated with

- a $birth\_cohort = 1920, \ldots, 2000$;
- a simulation start age, $sim\_start\_age = 40$, measured in $years$;
- a `person_clock` that measures time in the simulation;
- a biological sex, $sex \in \{\text{male, female}\}$;
- a `smoking_exposure` object that defines a smoking exposure personal history;
- a `toxin_exposure` object that defines a personal history of environmental or occupational toxin exposure;
- a `baseline_risk` object, which may include genetic information. Currently, this is limited to having Lynch Syndrome.

### Urinary Bladder

Table 1 describes the parameters we chose for the simulation of a urinary bladder.

All material © Copyright 2025 CISNET

**Table 1. Urinary bladder specifications.**

| Item or variable | Description | Value | Units | Explanation |
|---|---|---|---|---|
| $R_{bladder}$ | Bladder sphere radius | $4.92 \cdot 10^{-2}$ | $m$ | A distended bladder is a sphere that has 500 mL capacity. |
| $V_{bladder}$ | Bladder sphere volume | $5 \cdot 10^{-4}$ | $m^3$ | |
| $S_{bladder}$ | Bladder sphere area | $3.05 \cdot 10^{-2}$ | $m^2$ | |
| $M$ | Number of tiles | 678 | [tiles] | |
| $V(G_{678})$ | Dual graph of tiling, number of vertices | 678 | [vertices] | As many as the tiles |
| $E(G_{678})$ | Dual graph of tiling, number of edges | 2028 | [edges] | As many as the boundaries between tiles |
| Tiles | Area | $4.49 \cdot 10^{-5}$ | $m^2$ | |
| | Circumscribed circle diameter | $8.32 \cdot 10^{-3}$ | $m$ | |
| Trigone | Convex hull on the tesselation (vertices in $G_{678}$) | 1, 36, 86 | | 1: urethra, 36: left ureter, 86: right ureter |
| | Arc length between ureteral orifices | $5.00 \cdot 10^{-2}$ | $m$ | distended |
| | Actual trigone arc lengths | $(5.03, 5.06, 5.16) \cdot 10^{-2}$ | $m$ | based on the tesselation |
| | Actual dihedral (included) angles of trigone vertices | (1.21, 1.22, 1.25) | [radians] | about 69-71 degrees |
| $S_{trigone}$ | Area of trigone | $1.29 \cdot 10^{-3}$ | $m^2$ | $\approx$ 4% of $S_{bladder}$ |

An empty bladder is approximately tetrahedral and its lining (urothelium) forms mucosal folds. As it fills, it first becomes ellipsoidal and then approximately spherical, and its folds get flattened out. We assume that a fully distended bladder (e.g., during cystoscopy) can be satisfactorily approximated with a *convex polyhedron* inscribed in a $500\ mL$ sphere. The surface of the polyhedron approximates the surface of the bladder sphere by inducing a *polygonal tiling (tesselation)* $\mathcal{T}_M = \{\mathbf{T}_m \mid m = 1, \ldots, M\}$ of the sphere's surface, where $\mathbf{T}_m$ is the $m$-th facet (*tile*) and $M$ is the total number of the facets (*tiles*) of the polyhedron.

**Figure 1 ** shows the chosen tiling $\mathcal{T}_{678}$, which has $M = 678$ tiles with mean area about $0.45\ cm^2$. The diameter of the circle that circumscribes each hexagonal tile is approximately $0.8\ cm$. The *dual graph* $G_{678}$ of the tiling $\mathcal{T}_{678}$ has 678 vertices and 2028 edges. *Vertices* represent the centroids of the tiles and are shown as circularly-arranged small black and red dots. The three vertices that define the *trigone* (vertices 1, 36, and 86) are depicted larger and in red. *Undirected edges* (thin gray lines) connect vertices when the respective tiles share a border. Most tiles are hexagonal and have six adjacent tiles, thus most vertices have degree six.

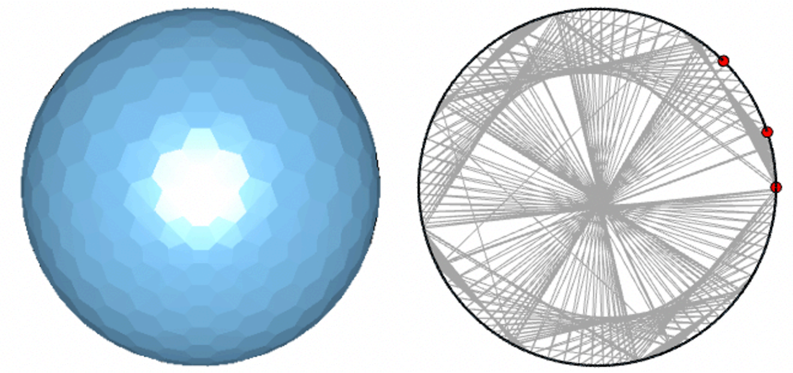

(a) $\mathcal{T}_{678}$: Tiling/polyhedral approximation of a sphere (b) $G_{678}$: The dual graph of the tiling $\mathcal{T}_{678}$

**Figure 1. Polyhedral approximation of the urinary bladder in the *Kystis*.**

---

## Bladder cancer lesion

A person may develop zero, one or several lesions. Precursor dysplastic lesions may appear and regress or grow to become benign or malignant tumors. We do not explicitly model the process of developing and

All material © Copyright 2025 CISNET

regressing dysplastic lesions. We model lesions conditional on that they will not regress. Such a non-regressing lesion

1. Starts on a single tile.

2. Starts as a noninvasive tumor.

3. Follows one of four archetypical growth patterns, which correspond to the WHO 2004 histological grading of bladder cancer tumors:

    - CIS: urothelial carcinoma *in situ*, which has a flat morphology and grows on the surface of the bladder.
    - VLIP: very low invasive potential. This type includes the WHO 2004 categories of *papillomas*, *inverted papillomas*, and *primarily papillary urothelial neoplasia with low malignant potential (PUNLMP)*. For simplicity, all VLIP lesions are assumed to be penduculated and papillary in appearance. Their risk of progression is assumed to be negligible.
    - HIP: higher invasive potential. This type includes urothelial neoplasias that can evolve to invasive cancer before developing symptoms. They can be papillary or non-papillary, but assumed to be more often sessile rather than penduculated. They include lesions that will evolve to low grade and high grade papillary urothelial carcinomas (LGPUC, HGPUC).
    - NUC: non-urothelial malignancies comprising squamous cell carcinomas and adenocarcinomas. They have a more-aggressive growth curve than the HIP category, so they become invasive at an earlier tumor burden. They are assumed to be non-papillary and sessile.

4. Has a $histology \in \{\text{HG} = \text{high grade}, \text{LG} = \text{low grade}\}$. Low-grade tends to grow slowly. High-grade is more likely to grow into the bladder wall and spread outside the bladder. Histology is important in determining the probability that a HIP will become muscle invasive.

5. Has a $morphology$ that is determined by the growth pattern. In terms of configuration morphology, a lesion may be flat, papillary, or non-papillary; and in terms of stalk morphology a lesion may be penduculated (with a stalk), sessile (protruding without a stalk) or flat (which also has no stalk). The stalk morphology determines the growth of the base area of a lesion as a function of the lesion's size or volume.

6. Has a $size$ measured in number of *cells*, which also determines a *volume* measured in $mm^3$. A lesion's size evolves over time following a Verhulst growth model.

7. Has a $base$, which is the part of the surface of the bladder that is occupied by the lesion. The lesion's base is represented as a set of contiguous tiles in $\mathcal{T}_M$, or, equivalently, a connected subgraph of $G_M$. We record which tiles of the base belong to the trigone because this is important for determining the probability of symptoms during micturition. As the lesion grows, so does its base; the growth curve for the base area is related to the growth curve of the lesion's number of cells, and the stalk morphology.

**Table 2** summarizes lesion stages, corresponding grades, and growth patterns in the `Kystis` model. The AJCC TNM staging system, commonly used for bladder cancer, describes the tumor's growth pattern and extent (T), lymph node involvement (N), and metastasis (M). `Kystis` can generate precancerous (PUNLMP) and benign (papillomas) lesions. It models non-muscle invasive cancer with Ta and Tis stages, which can progress to T1. `Kystis` does not differentiate between AJCC stages of muscle invasive cancer (T2, T3) and distant metastasis (M1a, M1b), and it does not model lymph node involvement (N). Lesion transitions are described in Model Assumptions.

**Table 2. Bladder cancer staging in the `Kystis`.**

| AJCC Staging System (8th edition, 2017) | Kystis Stage | Kystis Grade | Kystis Growth Pattern | Description |
|---|---|---|---|---|
| - | PUNLMP | - | VLIP | Papillary Urothelial Neoplasm of Low Malignant Potential; a pre-malignant tumor, very slow-growing and unlikely to spread. |
| - | Papilloma | - | VLIP | Urothelial papilloma, a rare benign tumor of the bladder. |

| AJCC Staging System (8th edition, 2017) | Kystis Stage | Kystis Grade | Kystis Growth Pattern | Description |
|---|---|---|---|---|
| Ta | Ta | Low, High | HIP | Non-invasive papillary carcinoma. It has grown toward the hollow center of the bladder but has not grown deeper into the connective tissue or muscle of the bladder wall. |
| Tis | Tis | - | CIS | Flat, non-invasive carcinoma. The cancer is growing in the inner lining layer of the bladder only. |
| T1 | T1 | Low, High | HIP, CIS, NUC | The cancer has grown into the layer of connective tissue under the lining layer of the bladder, but it has not reached the layer of muscle in the bladder wall. |
| T2a, T2b, T3a, T3b | Locally Advanced Disease (MIBC) | - | - | The cancer has grown through the muscle layer of the bladder (T2) or into the layer of fatty tissue surrounding the bladder (T3), but it has not spread to adjacent organs or distant parts of the body. |
| T4a, T4b, M1a, M1b | Metastasis | - | - | The cancer have spread into the prostate, seminal vesicles, uterus, vagina, pelvic or abdominal wall (T4) or to distant parts of the body (M1). |

## Lesion instantiation

In the `Kystis` model, the risk of lesion occurrence depends on person's demographic characteristics, such as sex, race and age, and exposure histories. We simulate lesions occurrence using non homogeneous Poisson point process (NHPPP). In an NHPPP, each event occurs independently of others, which doesn't accurately reflect the nature of cancer progression where existing lesions can influence the occurrence of new ones. Currently, we are extending the model functionality to implement Hawkes process for the generation of subsequent lesions. Hawkes process is self-exciting, meaning that each event increases the rate of future events temporarily.

## Lesion growth and progression

Once a person develops a lesion, it begins to grow following the Verhulst model, also known as the logistic growth model. The lesion grows rapidly at first, then slows as it approaches its carrying capacity. For Tis lesions, the carrying capacity is the number of cells that line half the surface of the bladder. For all other tumors, it is the number of tumor cells that occupy one-third of the volume of a distended bladder. The "steepness" of the growth curve is determined by the growth rate, which varies by lesion archetype (growth pattern).

Allowed lesion states and transitions are defined by the graph object. Papillomas, PUNLMPs, Tis, and low- and high-grade Ta tumors are primary lesions. PUNLMPs are not malignant but may occasionally evolve into malignant lesions, thus constituting precursor lesions. Papillomas and inverted papillomas are modeled as benign-only lesions that can manifest clinically. In the `Kystis` model, a lesion’s histologic grade is immutable once set at its inception (Ta-LG, Ta-HG). The model grows Ta lesions to T1 (T1-LG and T1-HG) without cross-transitions between the low- and high-grade paths. Tis lesions can become T1 lesions or lead directly to MIBC and then to metastatic disease. We use NHPPP to sample times of lesion transitions, where the instantaneous rate is a function of the size of the lesion.

## Symptoms

The `Kystis` model includes two types of symptoms: macroscopic hematuria (blood in the urine) and irritative symptoms during urination. The instantaneous rate of voiding symptoms is influenced by the location of the lesions (whether in the trigone or elsewhere) and the total area of the bladder covered by one or more lesions. The rate of hematuria is determined by the size of the lesion. Both symptom types are simulated using NHPPP.

## Clinical detection

We use NHPPP to simulate the clinical detection of bladder cancer. The instantaneous rate of clinical detection depends on sex, race, and the duration of bladder cancer symptoms, such as hematuria and voiding symptoms. Women and Black individuals tend to be diagnosed later on average due to the misattribution of symptoms to urinary infections for women or barriers to seeking care for non-Whites.[2]

## Exposure history

Exposure generator is currently limited to smoking history and utilises the CISNET Lung Cancer Group Smoking History Generator (SHG).[3] The SHG simulates life histories of people born in the U.S. between 1864 and 2100, tracking them up to age of 99. These histories include the year of birth and the age of death from all causes and causes other than lung cancer, conditional on smoking exposure. For each simulated person, the SHG simulates age they started smoking, smoking intensity defined as cigarettes per day at yearly intervals, and the age they quit smoking. Outputs are stratified by sex and race. The SHG does not account for recurrent smoking.

We adapted the SHG for R programming language and introduced sampling from NHPPP to draw times of smoking initiation, smoking sessation and deaths.

## Mortality

We sample death times using NHPPP based on data from the Human Mortality Database (overall mortality)[4] and the CISNET Lung Cancer Group Smoking History Generator[3] (mortality conditional on smoking history).

Bladder cancer death is modeled as one of the lesion transitions. A person can die from bladder cancer if one or more lesions progress to muscle-invasive disease or metastasis. The hazard of dying from bladder cancer at the non-muscle invasive stage (T1) is negligible; though it is still possible. The first occurrence of death, whether from bladder cancer or other causes, is the one that is taken into account.

## Surveillance

The surveillance module has not been implemented yet.

## Screening

The screening module has not been implemented yet.

# References

1. International Organization for Standardization. International Standard for the Representation of Dates and Times: ISO 8601 [Internet]. 2020. Available from: https://www.iso.org/iso-8601-date-and-time-format.html
2. NCI. SEER Cancer Stat Facts: Bladder Cancer 2019 [Internet]. 2019. Available from: https://seer.cancer.gov/statfacts/html/urinb.html
3. Jihyoun Jeon, Rafael Meza, Martin Krapcho, Lauren D Clarke, Jeff Byrne, David T Levy. Chapter 5: Actual and counterfactual smoking prevalence rates in the US population via microsimulation. Risk Analysis: An International Journal. Wiley Online Library; 2012;32:S51–S68.
4. John R Wilmoth, Kirill Andreev, Dmitri Jdanov, Dana A Glei, C Boe, M Bubenheim, et al. Methods protocol for the human mortality database. University of California, Berkeley, and Max Planck Institute for Demographic Research, Rostock URL: http://mortality org [version 31/05/2007]. 2007;9:10–11.

Reader's Guide
Model Purpose
Model Overview
Assumption Overview
Parameter Overview
Component Overview
Output Overview
Results Overview
Key References

# Output Overview

## Summary

Definitions and methodologies for the basic model outputs.

## Overview

The Kystis model simulates the U.S. population born between 1920 and 2000, encompassing their environmental exposures and the natural history of bladder cancer. This model generates two datasets containing person-level and lesion-level variables. These datasets are utilized to summarize population-level measures, including age- and stage-specific incidence of bladder cancer, and lifetime risk of developing bladder cancer. The outputs are stratified by race, sex, and exposure status.

## Output Listing

**Figure 1** illustrates the structure of the primary datasets generated by the Kystis model. The person-level dataset includes demographic variables, birth date, cohort, and spawn age, which is the age at the beginning of the simulation. Exposure variables in the person-level dataset are limited to ever-exposed status. However, detailed smoking history can be obtained from the Smoking History Generator dataset linked by `person_id`, which provides information on smoking initiation age, smoking cessation age, and smoking intensity measured in cigarettes per day. The variable `toxin_ever_exposed` serves as a placeholder for occupational and environmental exposures that will be incorporated in future model releases. Variables representing the natural history include ages at two types of modeled symptoms (voiding symptoms and macroscopic bleeding), age and stage at clinical detection, and ages at various stages of bladder cancer (NMIBC, MIBC, metastasis). The person-level dataset also includes bladder cancer mortality and mortality from other causes, with `age_dead_all_causes` representing the minimum of these two.

The person-level and lesion-level datasets are linked by `person_id`. The lesion-level dataset is in a long format and includes variables related to lesions (`lesion_id`) and lesion transitions (`transition_id`). Lesion-level variables encompass the inception moment of the lesion, i.e., the date when the lesion appeared, and the persons's age at that moment, as well as lesion morphology. Each lesion transition is described with a state name, moment at state, and age at state on both the lesion's and persons's clock. Additionally, each state is categorized as precancerous lesion (`is_VLIP`), low-grade lesion (`is_HIP_LG`), non-muscle invasive bladder cancer (`is_NMIBS`), muscle invasive bladder cancer (`is_MIBS`), metastatic disease (`is_metastasis`), or the terminal state (`is_bcdeath`).

All material © Copyright 2025 CISNET

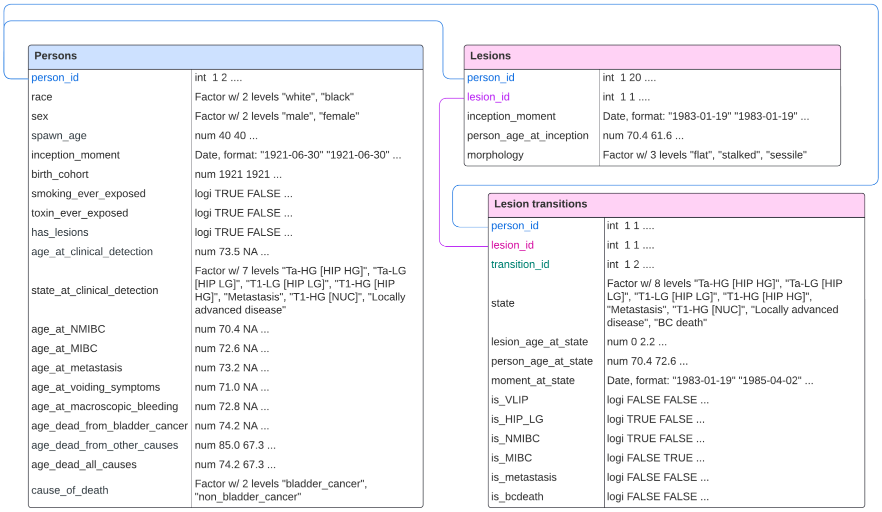


**Figure 1. Bladder cancer natural history data sets in the *Kystis* model.**

Population-level outputs include age- and stage-specific incidence of bladder cancer and lifetime risk of developing bladder cancer (Table 1).

**Table 1. Population-level outputs in the `Kystis` model.**

| Output | Definition | Description |
| --- | --- | --- |
| Age-specific incidence rate of bladder cancer per 100,000 | $I_a = 10^5 \frac{C_a}{N_a}$ | $C_a$ is a number of people diagnosed with bladder cancer and $N_a$ is a total population at risk in the age interval $a$. We use SEER 5-year age interval breaks starting from the age of 40, which is a spawn age in the model. Ages over 85 years are groupped as 85+ category. |
| Age-specific cumulative incidence rate of bladder cancer per 100,000, by stage | $I_{a,s} = 10^5 \frac{C_{a,s}}{N_{a,s}}$ | $s \in \{$"NMIBS low grade", "NMIBS, high grade", "MIBS", "metastasis"$\}$. |
| Lifetime risk of developing bladder cancer | $LR = \sum_{a=0}^{K} I_a * S_a$ | $K$ is the allowed maximum age in the simulation, $I_a$ is age-specific incidence of bladder cancer at age $a$, and $S_a$ is a probability of surviving by age $a$. |
| Mean sojourn time | $ST = \frac{\sum_i D_i - O_i}{C}$ | $D_i$ is person's age of bladder cancer clinical detection, $O_i$ is person's age at the first lesion inception moment, and $C$ is a total number of people diagnosed with bladder cancer. |

Reader's Guide
Model Purpose
Model Overview
Assumption Overview
Parameter Overview
Component Overview
Output Overview
Results Overview
Key References

# Results Overview

## Summary

A guide to the results obtained from the model.

## Overview

In the current version, we calibrated parameters of the natural history of bladder cancer for White Men, the population subgroup with the highest incidence of bladder cancer. We compared simulated data versus corresponding 2010 SEER estimates for a population-based simulation. We simulated the US population as stacked cohorts of people born between 1910 and 2010 (covering 0-100 years of age in 2010) with relative cohort sizes proportional to CENSUS data. We compared the simulated incidence in 5-year age groups (i.e., 40-44, 45-49, …, 80-84, 85+) with the corresponding age groups for the 2010 diagnosis year. The model was calibrated using EGO algorithm variant and BayCANN.[1,2]We used Latin-Hypercube sampling to generate design points and used the Poisson (pseudo)-likelihood as a calibration objective.

Additionaly, we examined how the calibrated model simulate key events, that are not directly observable in the data, such as tumor emergence, MIBC, and metastasis. We calculated age distributions at these events and the distributions of the time intervals between them for a cohort of White Men born in 1950.

## Results

**Figures 1 & 2** compare the simulated versus observed age- and stage-specific incidence of all bladder cancers at diagnosis in White Men for 2010. For almost all age- and stage-groups, the uncertainty intervals of the simulated and observed incidences overlap, indicating good agreement.

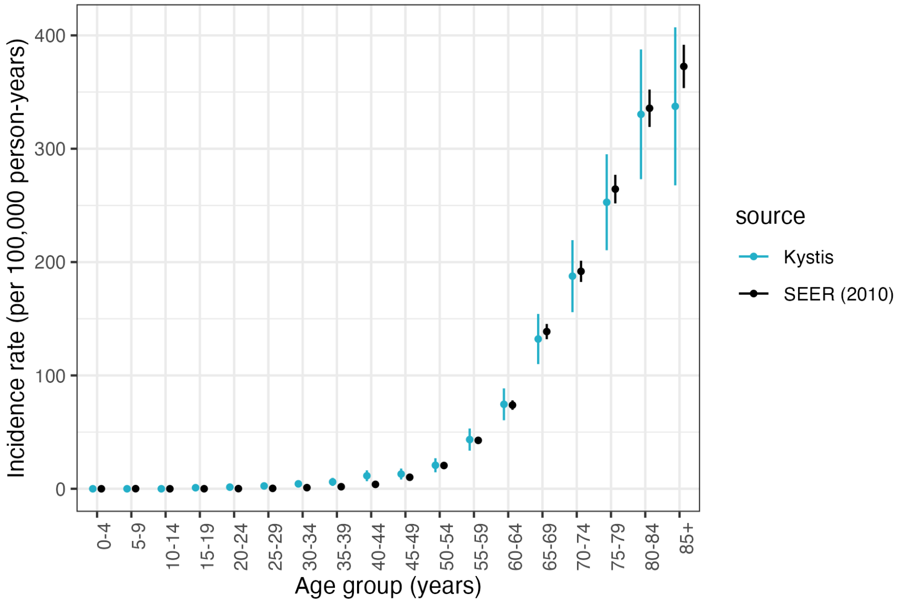


**Figure 1. Age-specific bladder cancer incidence in White Men (U.S. in 2010).**

All material © Copyright 2025 CISNET

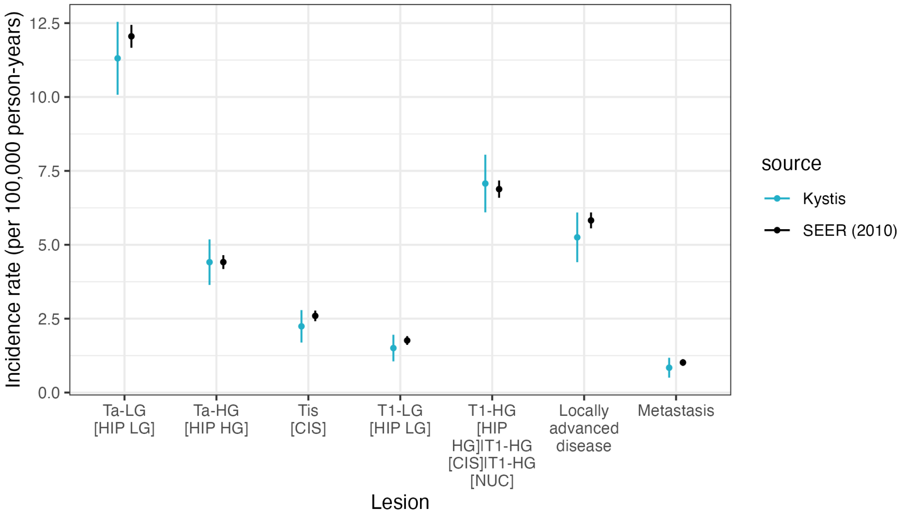


**Figure 2. Stage-specific bladder cancer incidence in White Men (U.S. in 2010).**

**Figure 4** illustrates the lifetime risk of being diagnosed with bladder cancer. According to the model, individuals born in 1900 had an estimated lifetime risk of 2.0 percent. This risk gradually increased over time, following changes in life expectancy and smoking exposure. Estimates from Kystis indicate that lifetime risk peaked at 3.6 percent for those born between 1940 and 1950, then declined before rising again among the 1970 to 1980 birth cohorts.

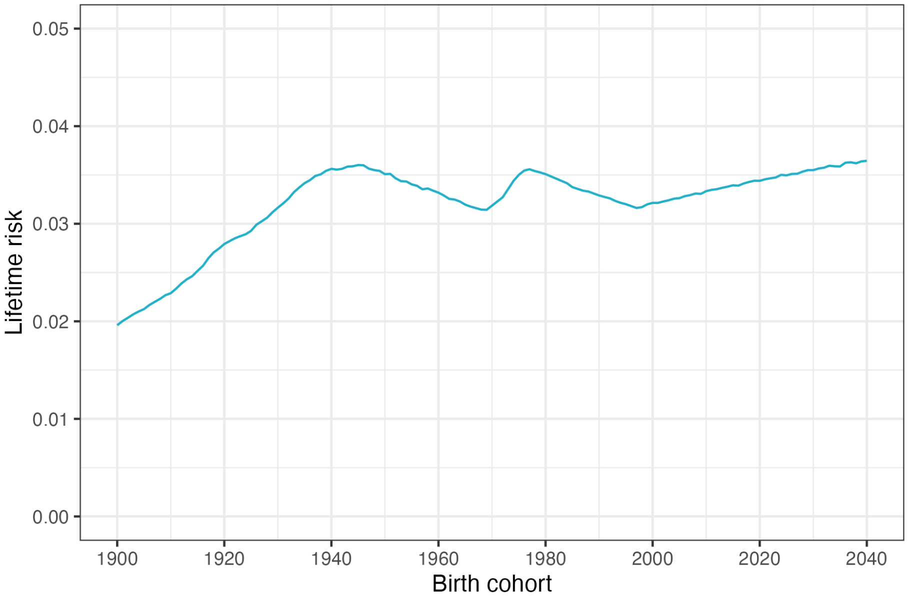


**Figure 3. Bladder cancer lifetime risk in White Men (U.S., 1900-2040).**

**Figure 4a** presents the predicted age-standardized incidence of bladder cancer from 2000 to 2040, using the US 2000 standard population as a reference. The model predictions align qualitatively with SEER data from 2000 to 2022, showing a slight decline in age-standardized incidence. SEER data indicates that incidence remained stable through 2004, followed by an annual decline of 1% from 2005 to 2022. `Kystis` estimates a more gradual decline of 0.6% per year between 2005 and 2021. The projected age-standardized incidence for 2040 is approximately 31.1 new cases per 100,000 person-years.

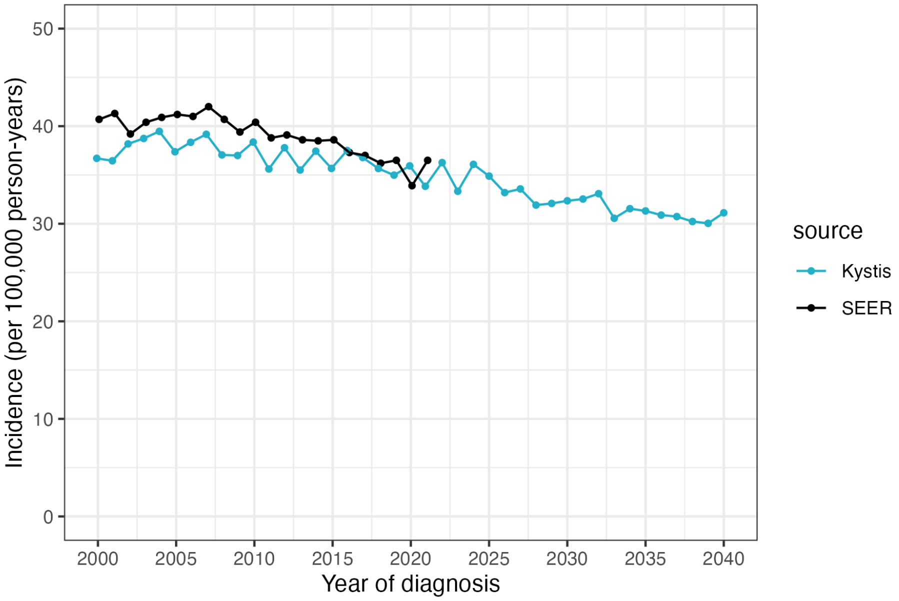


**Figure 4a. Annual bladder cancer incidence in White Men (Age-adjusted estimates, U.S. 2000 standard population).**

Projections of age-standardized incidence do not fully capture the expected changes in the population burden of bladder cancer. **Figure 4b** presents projections of the crude annual incidence of bladder cancer through 2040. According to `Kystis`, the annual incidence rate is expected to rise steadily from approximately 37 cases per 100,000 person-years in 2010 to around 45 cases per 100,000 person-years by 2040.

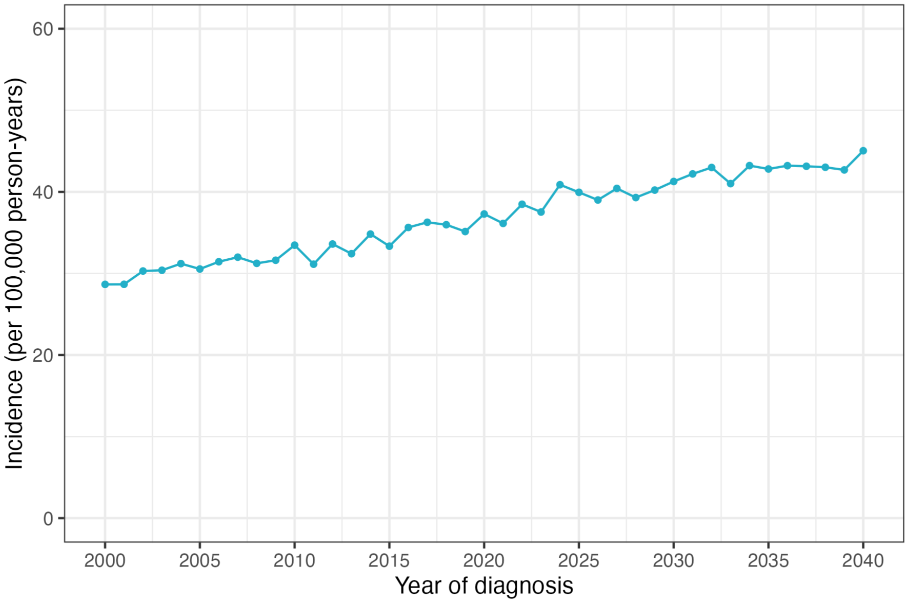


**Figure 4b. Annual bladder cancer incidence in White Men (Crude estimates, U.S. population).**

**Figure 5** shows predicted annual prevalence of bladder cancer. According to the model, bladder cancer prevalence is slowly decreasing by approximately 0.1% per year on average.

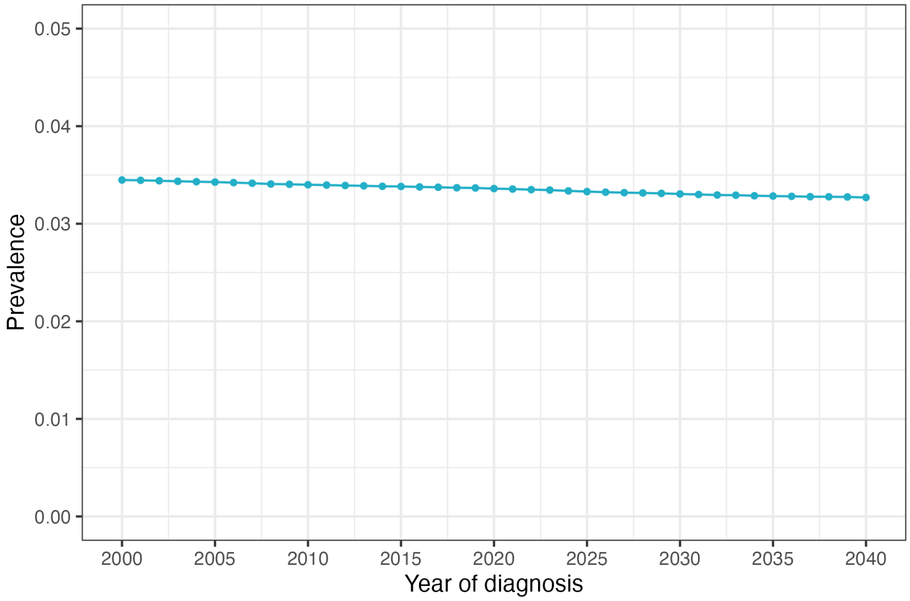


**Figure 5. Annual bladder cancer prevalence in White Men (U.S. population).**

**Table 1** shows the median and 25-th and 75-th percentiles of the ages of key events in the natural history of bladder cancer as well as the time intervals between key events for the three models for White Men born in 1950. The median interval between the time when a lesion is detectable and diagnosis is about 3.3 years. The median time to MIBC from lesion emergence is about 2.7 years, and from MIBC to metastasis is about 1 year, with a wide distribution across simulated individuals.

**Table 1. Ages of and time intervals between key events (White Men, 1950 cohort).**

| Description | Median (IQR) |
|---|---|
| **Ages** | |
| First lesion emergence | 73.0 (63.7, 80.7) |
| Diagnosis | 74.5 (65.7, 82.0) |
| MIBC | 75.9 (67.1, 83.0) |
| Metastasis | 76.4 (67.8, 83.6) |
| **Time intervals, years** | |
| Emergence to diagnosis | 3.3 (2.7, 5.3) |
| Emergence to MIBC | 2.7 (1.6, 4.4) |
| MIBC to metastasis | 1.0 (0.4, 2.1) |

# References

1. Donald R Jones, Matthias Schonlau, William J Welch. Efficient global optimization of expensive black-box functions. Journal of Global optimization. Springer; 1998;13:455–492.
2. Hawre Jalal, Thomas A Trikalinos, Fernando Alarid-Escudero. BayCANN: streamlining Bayesian calibration with artificial neural network metamodeling. Frontiers in Physiology. Frontiers Media SA; 2021;12:662314.

Reader's Guide
Model Purpose
Model Overview
Assumption Overview
Parameter Overview
Component Overview
Output Overview
Results Overview
Key References

# Key References

Elenir BC Avritscher, Catherine D Cooksley, H Barton Grossman, Anita L Sabichi, Lois Hamblin, Colin P Dinney, et al. Clinical Model of Lifetime Cost of Treating Bladder Cancer and Associated Complications. Urology. Elsevier; 2006;68(3):549–553.

Nicolas Bacaër. Verhulst and the logistic equation (1838). A short history of mathematical population dynamics. London: Springer London; 2011. p. 35–39.

Maximilian Burger, James WF Catto, Guido Dalbagni, H Barton Grossman, Harry Herr, Pierre Karakiewicz, et al. Epidemiology and Risk Factors of Urothelial Bladder Cancer. European Urology. Elsevier; 2013;63(2):234–241.

Marcus GK Cumberbatch, Ibrahim Jubber, Peter C Black, Francesco Esperto, Jonine D Figueroa, Ashish M Kamat, et al. Epidemiology of Bladder Cancer: A Systematic Review and Contemporary Update of Risk Factors in 2018. European Urology. Elsevier; 2018;74(6):784–795.

Marcus GK Cumberbatch, Aidan P Noon. Epidemiology, Aetiology and Screening of Bladder Cancer. Translational Andrology and Urology. AME Publications; 2019;8(1):5.

Jakub Dobruch, Siamak Daneshmand, Margit Fisch, Yair Lotan, Aidan P Noon, Matthew J Resnick, et al. Gender and Bladder Cancer: A Collaborative Review of Etiology, Biology, and Outcomes. European Urology. Elsevier; 2016;69(2):300–310.

Jonathan J Duplisea, Sharada Mokkapati, Devin Plote, Kimberly S Schluns, David J McConkey, Seppo Yla-Herttuala, et al. The development of interferon-based gene therapy for BCG unresponsive bladder cancer: from bench to bedside. World Journal of Urology. Springer; 2019;37:2041–2049.

Neal D Freedman, Debra T Silverman, Albert R Hollenbeck, Arthur Schatzkin, Christian C Abnet. Association between smoking and risk of bladder cancer among men and women. Jama. American Medical Association; 2011;306(7):737–745.

Petrisor Aurelian Geavlete. Endoscopic Diagnosis and Treatment in Urinary Bladder Pathology: Handbook of Endourology. Academic Press; 2016.

Dharmesh Gopalakrishnan, Vadim S Koshkin, Moshe C Ornstein, Athanasios Papatsoris, Petros Grivas. Immune Checkpoint Inhibitors in Urothelial Cancer: Recent Updates and Future Outlook. Therapeutics and Clinical Risk Management. Taylor & Francis; 2018;14:1019–1040.

International Organization for Standardization. International Standard for the Representation of Dates and Times: ISO 8601 [Internet]. 2020. Available from: https://www.iso.org/iso-8601-date-and-time-format.html

Hawre Jalal, Thomas A Trikalinos, Fernando Alarid-Escudero. BayCANN: streamlining Bayesian calibration with artificial neural network metamodeling. Frontiers in Physiology. Frontiers Media SA; 2021;12:662314.

Jihyoun Jeon, Rafael Meza, Martin Krapcho, Lauren D Clarke, Jeff Byrne, David T Levy. Chapter 5: Actual and counterfactual smoking prevalence rates in the US population via microsimulation. Risk Analysis: An International Journal. Wiley Online Library; 2012;32:S51–S68.

Donald R Jones, Matthias Schonlau, William J Welch. Efficient global optimization of expensive black-box functions. Journal of Global optimization. Springer; 1998;13:455–492.

Jack PC Kleijnen. Antithetic variates, common random numbers and optimal computer time allocation in simulation. Management Science. INFORMS; 1975;21(10):1176–1185.

Pierre L’Ecuyer, Christian Lécot, Bruno Tuffin. A randomized quasi-Monte Carlo simulation method for Markov chains. Operations research. INFORMS; 2008;56(4):958–975.

Virginia A Moyer. Screening for bladder cancer: US Preventive Services Task Force recommendation statement. Annals of internal medicine. American College of Physicians; 2011;155(4):246–251.

NCI. SEER Cancer Stat Facts: Bladder Cancer 2019 [Internet]. 2019. Available from: https://seer.cancer.gov/statfacts/html/urinb.html

Cyrill A Rentsch, Claire Biot, Joël R Gsponer, Alexander Bachmann, Matthew L Albert, Romulus Breban. BCG-Mediated Bladder Cancer Immunotherapy: Identifying Determinants of Treatment Response Using a Calibrated Mathematical Model. PloS one. 2013 Feb;8(2):e56327-17.

Rebecca L Siegel, Kimberly D Miller, Hannah E Fuchs, Ahmedin Jemal. Cancer Statistics, 2022. CA: A Cancer Journal for Clinicians. 2022;72(1).

John A Spratt, D Von Fournier, John S Spratt, Ernst E Weber. Decelerating growth and human breast cancer. Cancer. Wiley Online Library; 1993;71(6):2013–2019.

Robert S Svatek, Brent K Hollenbeck, Sten Holmäng, Richard Lee, Simon P Kim, Arnulf Stenzl, et al. The Economics of Bladder Cancer: Costs and Considerations of Caring for This Disease. European Urology. Elsevier; 2014;66(2):253–262.

Thomas A Trikalinos, Yuliia Sereda. The nhppp package for simulating non-homogeneous Poisson point processes in R. PLoS One. Public Library of Science San Francisco, CA USA; 2024;19(11):e0311311.

Thomas A Trikalinos, Yuliia Sereda. nhppp: Simulating Nonhomogeneous Poisson Point Processes in R. arXiv preprint arXiv:240200358. 2024;

Panagiotis J Vlachostergios, Christopher D Jakubowski, Muhammad J Niaz, Aileen Lee, Charlene Thomas, Amy L Hackett, et al. Antibody-drug conjugates in bladder cancer. Bladder Cancer. IOS Press; 2018;4(3):247–259.

Yu Wang, Qian Chang, Yang Li. Racial differences in urinary bladder cancer in the United States. Scientific Reports. Nature Publishing Group UK London; 2018;8(1):12521.

Adam B Weiner, Mary-Kate Keeter, Adarsh Manjunath, Joshua J Meeks. Discrepancies in staging, treatment, and delays to treatment may explain disparities in bladder cancer outcomes: an update from the National Cancer Data Base (2004–2013). Urologic Oncology: Seminars and Original Investigations. Elsevier; 2018. p. 237-e9.

John R Wilmoth, Kirill Andreev, Dmitri Jdanov, Dana A Glei, C Boe, M Bubenheim, et al. Methods protocol for the human mortality database. University of California, Berkeley, and Max Planck Institute for Demographic Research, Rostock URL: http://mortality org [version 31/05/2007]. 2007;9:10–11.

Wesley Yip, Giovanni Cacciamani, Sumeet K Bhanvadia. Disparities in bladder cancer outcomes based on key sociodemographic characteristics. Current Urology Reports. Springer; 2020;21:1–6.

*All material © Copyright 2025 CISNET*
