# Supplementary material for: Bladder Cancer Burden in the USA: Population Scenarios for 2040
Source: Eur Urol Open Sci. 2025 Dec 4;83:99–108. doi: 10.1016/j.euros.2025.11.013 (PMC12721285; doi:10.1016/j.euros.2025.11.013)
Supplement: Supplementary Data 3 [file mmc3.pdf]

SCOUT (Columbia University)

BLADDER

Columbia University
MPD Version:
1.0.00

Simulation of Cancers of the Urinary Tract (SCOUT)

Columbia University Irving Medical Center

Reader's Guide
Model Purpose
Model Overview
Assumption Overview
Parameter Overview
Component Overview
Output Overview
Results Overview
Key References

Contact

Stella Kang (sk5603@cumc.columbia.edu)

Suggested Citation

Forthcoming.

Version Table

Version	Release Date	Notes	Publications
1.0.00	2025-02-27	Initial version of the micro-simulation model (bladder cancer natural history).	N/A

All material © Copyright 2025 CISNET

Reader's Guide
Model Purpose
Model Overview
Assumption Overview
Parameter Overview
Component Overview
Output Overview
Results Overview
Key References

Reader's Guide

Core Profile Documentation

These topics will provide an overview of the model without the burden of detail. Each can be read in about 5-10 minutes. Each contains links to more detailed information if required.

Model Purpose
This document describes the primary purpose of the model.

Model Overview
This document describes the primary aims and general purposes of this modeling effort.

Assumption Overview
An overview of the basic assumptions inherent in this model.

Parameter Overview
Describes the basic parameter set used to inform the model, more detailed information is available for each specific parameter.

Component Overview
A description of the basic computational building blocks (components) of the model.

Output Overview
Definitons and methodologies for the basic model outputs.

Results Overview
A guide to the results obtained from the model.

Key References
A list of references used in the development of the model.

Further Reading

These topics will provide a intermediate level view of the model. Consider these documents if you are interested gaining in a working knowledge of the model, its inputs and outputs.

Advanced Reading

These topics denote more detailed documentation about specific and important aspects of the model structure

All material © Copyright 2025 CISNET

SCOUT (Columbia University)

BLADDER

Columbia University
Model Purpose

Reader's Guide
Model Purpose
Model Overview
Assumption Overview
Parameter Overview
Component Overview
Output Overview
Results Overview
Key References

Model Purpose

Summary

This document describes in broad strokes the purpose for the development of the Simulation of Cancers of the Urinary Tract (SCOUT) Model.

Purpose

The prevalence of chronic kidney disease (CKD) is high among older adults,[1] persons with hypertension,[2] and/or those with diabetes.[2] In turn, CKD is associated with increased risk of cardiovascular mortality [3] and also mortality related to specific cancers.[4,5] Specifically, the urinary tract (kidney, ureters, bladder) is the source of the second-most common cause of cancer deaths in this population.[4] CKD and smoking are independently associated with the risk of urinary tract cancer, including bladder cancer.[6,7] SCOUT incorporates major risk factors and comorbid conditions contributing to the risk of bladder cancer, cancer-specific mortality risk, and other competing mortality risks. By incorporating risk categories defined by individual characteristics and risk factors, SCOUT is enabled for assessing the comparative effectiveness of screening and management strategies for bladder cancer in both the general population as well as specific populations with higher incidence of bladder cancers.

The purpose of the SCOUT model can be summarized in four aims:

1. Simulate a hypothetical population of individuals with and without CKD with broad-ranging risk for urinary system cancers and competing mortality risks
2. Simulate bladder cancer incidence and mortality in individuals with and without CKD according to the nationally representative data
3. Incorporate bladder cancer treatment pathways according to the clinical guidelines along with post-treatment events
4. Identify whether circumstances exist under which benefits outweigh the harms of urinary system cancer screening in the general population or in subpopulations with elevated risk of bladder cancer

References

1. United States Renal Data System. 2023 USRDS Annual Data Report: Epidemiology of kidney disease in the United States [Internet]. 2023. Available from: https://adr.usrds.org/2023
2. A. X. Garg, B. A. Kiberd, W. F. Clark, R. B. Haynes, C. M. Clase. Albuminuria and renal insufficiency prevalence guides population screening: results from the NHANES III. Kidney Int. 2002;61(6):2165–75.
3. A. S. Go, G. M. Chertow, D. Fan, C. E. McCulloch, C. Y. Hsu. Chronic kidney disease and the risks of death, cardiovascular events, and hospitalization. N Engl J Med. 2004;351(13):1296–305.
4. S. D. Navaneethan, J. D. Schold, S. Arrigain, S. E. Jolly, Jr. Nally J. V. Cause-Specific Deaths in Non-Dialysis-Dependent CKD. J Am Soc Nephrol. 2015;26(10):2512–20.
5. A. Christensson, C. Savage, D. D. Sjoberg, A. M. Cronin, M. F. O'Brien, W. Lowrance, et al. Association of cancer with moderately impaired renal function at baseline in a large, representative, population-based cohort followed for up to 30 years. Int J Cancer. 2013;133(6):1452–8.
6. W. T. Lowrance, J. Ordonez, N. Udaltsova, P. Russo, A. S. Go. CKD and the risk of incident cancer. J Am Soc Nephrol. 2014;25(10):2327–34.
7. N. D. Freedman, D. T. Silverman, A. R. Hollenbeck, A. Schatzkin, C. C. Abnet. Association between smoking and risk of bladder cancer among men and women. JAMA. 2011;306(7):737–45.

All material © Copyright 2025 CISNET

SCOUT (Columbia University)

BLADDER

Columbia University
Model Overview

Reader's Guide
Model Purpose
Model Overview
Assumption Overview
Parameter Overview
Component Overview
Output Overview
Results Overview
Key References

Model Overview

Summary

This document provides a summary of the Simulation of Cancers of the Urinary Tract (SCOUT) model. SCOUT is a microsimulation model that simulates a hypothetical cohort with established risk factors for bladder cancers such as chronic kidney disease, smoking, and hypertension.

Purpose

The SCOUT model is developed to assess the comparative effectiveness of screening in general and specific populations for bladder cancers, while accounting for major risk factors including CKD severity, smoking, sex, age, and race. See Model Purpose for more details.

Background

Chronic kidney disease (CKD) is a prevalent condition that is associated with multiple other disease, including cancer. The prevalence of CKD is approximately 14% among U.S. adults overall and 33.2% among older adults (≥65 years).[1] Persons with CKD are at risk for early mortality compared to those with normal kidney function. According to a retrospective cohort study using the third National Health and Nutrition Examination Survey, the 10-year cumulative cardiovascular mortality rate was 9.9% (95% CI: 7.9–11.9%) among individuals with kidney disease, compared to 3.4% (95% CI: 3.1–3.7%) among healthy individuals.[2] Furthermore, a recent systematic review and meta-analysis reported that persons with CKD are 1.35 times more likely to develop urothelial cancers compared to those with preserved kidney function.[3]

Among the types of urinary tract cancers, bladder cancers have the highest incidence. In 2023, an estimated 82,290 of new cases of bladder cancer were diagnosed making it the sixth highest incident cancer in the US.[4] Moreover, urinary bladder cancers were the eighth most common cause of cancer-specific deaths in 2023 resulting in an estimated 16,710 deaths.[4] Despite the significant burden of bladder cancer and the availability of increasingly accurate detection tests, there are currently no recommendations for urinary tract cancer screening programs. Use of microsimulation modeling can serve as a valuable tool for addressing health policy questions regarding risk-based urinary bladder screening approaches among high-risk individuals such as persons with CKD, where early stage disease is crucial for the greatest chance at effective therapy. This decision-analytic tool can be applied to the design and guidance of randomized controlled trials as new technologies and risk information are available, aiming to improve in early-stage diagnosis and treatment.

Model Description

The SCOUT model is a discrete-time non-homogenous Markov microsimulation model. Our model simulates persons with various risk factors and comorbidities relevant to risk of urinary tract cancer including sex, tobacco smoking status, diabetes mellitus, hypertension, and CKD. SCOUT first initiates a hypothetical cohort of the same sex and race at the age of 30 with individual risk factors. A simulated person receives a static risk factor of smoking status (Never, Former, Current) and time-varying risk factors of diabetes mellitus, hypertension, and CKD. Specifically, each person may develop diabetes and/or hypertension over their lifetime, which further influences the development and progression of CKD and bladder cancer (**Figure 1**). SCOUT is written in Python.

All material © Copyright 2025 CISNET

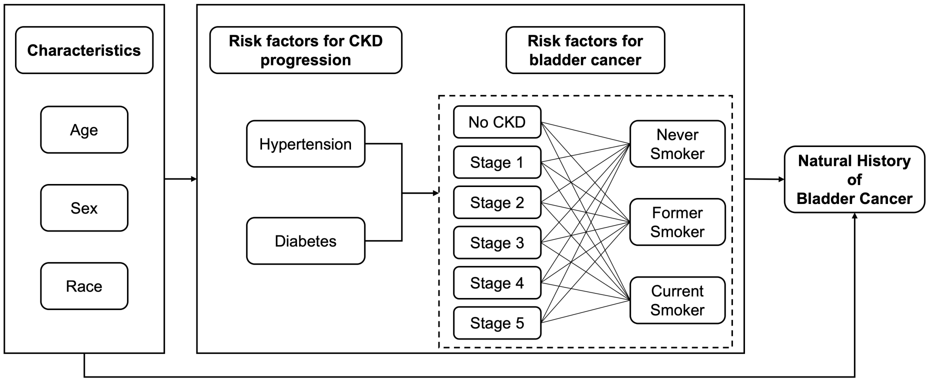

Figure 1. Individual characteristics and risk factors associated with CKD progression and natural history of bladder cancer in the SCOUT model.

SCOUT consists of 4 components:

1. Chronic kidney disease development and progression
2. Natural history of bladder cancer
3. Screening (in development)
4. Treatment and surveillance (in development)

Chronic kidney disease

SCOUT simulates the development and progression of CKD with seven stages (no CKD, stage 1, stage 2, stage 3a, stage 3b, stage 4, stage 5) using a yearly cycle. The CKD stage is defined by glomerular filtration rate (GFR) and albuminuria categories (normal, microalbuminuria, macroalbuminuria) according to Kidney Diseases: Improving global Outcomes (KDIGO).[5] At the beginning of the time horizon, each simulated person is assigned diabetes and/or hypertension status (presence or absence), GFR, and albuminuria level of normal or micro-albuminuria. At each cycle, a person may develop hypertension and/or diabetes, experience a decline in GFR, develop micro-albuminuria or have a transition from micro- to macro-albuminuria. The rates of GFR decline, incidence rates of microalbuminuria, and the transition probability from micro- to macroalbuminuria depends on age, sex, and presence/absence of hypertension and diabetes.[6-8] A person with CKD progresses in increasing severity of CKD stages. The severity of CKD stages further informs the risk of bladder cancer, non-cardiovascular disease (CVD) and CVD mortality risk. The CKD component was calibrated to the prevalence of CKD and incidence of end-stage renal disease obtained from the United States Renal Data System (USRDS) report.[1] See Chronic Kidney Disease component for more details.

Natural history of bladder cancer

SCOUT simulates the development and progression of bladder cancers with four main health states: disease-free, non-muscle invasive bladder cancers or NMIBC (Ta, Tis, T1), muscle invasive bladder cancers or MIBC (T2, T3, T4 excluding metastatic disease), and metastasis using a monthly cycle. At the beginning of the time horizon, each simulated person is assigned established risk factors associated with bladder cancer including static smoking status, and CKD stage as described in the CKD component. At each cycle, a person may experience one of the following: remain tumor-free, develop low-grade Ta or high-grade Ta that is initially undetectable (presence of tumor without detectable clinical signs or symptoms), transition from undetectable to detectable state (with clinical signs or symptoms) for those with NMIBC, transition from detectable to detected stage (for those with NMIBC), transition from low to high grade (for those with NMIBC), and experience an increase in T stage. The probability of developing tumors depends on age, sex, smoking status, and CKD stage. For those with bladder tumors, SCOUT applied the probability transitioning from less severe to more severe health states depending on age and sex. All probabilities applied to the model are updated monthly and are conditional probabilities depending only on the health state from the previous month. Once a person reach metastasis, they are subject to metastatic death in addition to other causes of death including non-CVD and CVD. The natural history of bladder cancer was first calibrated to SEER observed cancer incidence for white men, given the predominance of bladder cancer diagnoses occurring in this population. See Natural history of bladder cancer component for more details.

References

1. United States Renal Data System. 2023 USRDS Annual Data Report: Epidemiology of kidney disease in the United States [Internet]. 2023. Available from: https://adr.usrds.org/2023
2. M. Afkarian, M. C. Sachs, B. Kestenbaum, I. B. Hirsch, K. R. Tuttle, J. Hinnmelfarb, et al. Kidney Disease and Increased Mortality Risk in Type 2 Diabetes. Journal of the American Society of Nephrology. 2013;24(2):302–308.
3. Emily R Brooks, Mutita Siriruchatanon, Vinay Prabhu, David M Charytan, William C Huang, Yu Chen, et al. Chronic kidney disease and risk of kidney or urothelial malignancy: systematic review and meta-analysis. Nephrology Dialysis Transplantation. 2023;
4. R. L. Siegel, K. D. Miller, N. S. Wagle, A. Jemal. Cancer statistics, 2023. CA Cancer Journal for Clinicians. 2023;73(1):17–48.
5. A. Levin, P. E. Stevens. Summary of KDIGO 2012 CKD Guideline: behind the scenes, need for guidance, and a framework for moving forward. Kidney Int. 2014;85(1):49–61.
6. L. E. Boulware, B. G. Jaar, M. E. Tarver-Carr, F. L. Brancati, N. R. Powe. Screening for proteinuria in US adults: a cost-effectiveness analysis. JAMA. 2003;290(23):3101–14.
7. B. O. Yarnoff, T. J. Hoerger, S. S. Shrestha, S. K. Simpson, N. R. Burrows, A. H. Anderson, et al. Modeling the impact of obesity on the lifetime risk of chronic kidney disease in the United States using updated estimates of GFR progression from the CRIC study. PLoS One. 2018;13(10):e0205530.
8. T. J. Hoerger, J. S. Wittenborn, J. E. Segel, N. R. Burrows, K. Imai, P. Eggers, et al. A health policy model of CKD: 1. Model construction, assumptions, and validation of health consequences. Am J Kidney Dis. 2010;55(3):452–62.

All material © Copyright 2025 CISNET

Columbia University
Assumption Overview

Reader's Guide
Model Purpose
Model Overview
Assumption Overview
Parameter Overview
Component Overview
Output Overview
Results Overview
Key References

Assumption Overview

Summary

An overview of the basic assumptions inherent in this model. This document briefly describes the assumptions made by the Simulation of Cancers of the Urinary Tract (SCOUT) model.

Background

To create a microsimulation model, assumptions are required to simplify the complexity of disease progression.

Assumption Listing

Model assumptions are listed for each component of the SCOUT model.

Chronic kidney disease

- Initial distribution of GFR for adults of age 30 years follows the truncated normal distribution.
- Systolic blood pressure by age, sex, and CKD status is assumed to follow a distribution based on percentile rank values derived from NHANES III.
- For adults of age 30, the prevalence of diabetes and microalbuminuria is assumed to be zero.
- Albumin-to-creatinine ratio (ACR) is assumed to be the only marker of kidney damage in the current version of SCOUT. Other markers such as urine sediment abnormalities, history of kidney transplant, or electrolyte and other abnormalities due to tubular disorders are excluded from the model.
- The annual rate of decline in GFR is assumed to be heterogeneous among the simulated cohort with the same risk factors, achieved by applying an individual coefficient multiplier following a symmetric triangular distribution.
- Higher rates of CKD progression are applied to persons who develop diabetes and/or hypertension after a duration of two cycles.
- The mortality rate for end-stage renal disease is applied to persons who reach CKD stage 5 after a duration of one cycle.

Natural history of bladder cancer

- The undetectable tumor state refers to a presence of bladder cancer tumor without any sign of symptoms.
- The detectable tumor state refers to bladder cancer tumor that exhibits common symptoms such as microscopic or macroscopic hematuria.
- The detected tumor state refers to any bladder cancer tumors that are clinically diagnosed.
- Any muscle invasive bladder cancers (MIBC T2-T4 excluding metastatic disease) are assumed to be symptomatic and detectable.
- For each of the T stages among MIBC, we assumed a proportion of these tumors are detected, where a higher proportion of tumors is detected in more severe T stages.
- We assumed the transition time from undetectable to detectable TaHG is no longer than 1.5 years.[1] Consequently, the transition time from undetectable to detectable TaLG is assumed to be longer than that of TaHG, while the transitioning time for T1 is assumed to be shorter.[2-6]
- The probability of developing a bladder tumor and the probability of transitioning between T stages increase with age.

References

1. H. Calkins, Y. Shyr, H. Frumin, A. Schork, F. Morady. The value of the clinical history in the differentiation of syncope due to ventricular tachycardia, atrioventricular block, and neurocardiogenic syncope. Am J Med. 1995;98(4):365–73.
2. Y. Lotan, K. Elias, R. S. Svatek, A. Bagrodia, G. Nuss, B. Moran, et al. Bladder cancer screening in a high risk asymptomatic population using a point of care urine based protein tumor marker. J Urol. 2009;182(1):52–7; discussion 58.
3. E. M. Messing, T. B. Young, V. B. Hunt, E. B. Roecker, A. M. Vaillancourt, W. J. Hisgen, et al. Home screening for hematuria: results of a multiclinic study. J Urol. 1992;148(2 Pt 1):289–92.
4. E. M. Messing, T. B. Young, V. B. Hunt, J. M. Wehbie, P. Rust. Urinary tract cancers found by homescreening with hematuria dipsticks in healthy men over 50 years of age. Cancer. 1989;64(11):2361–7.
5. E. M. Messing, T. B. Young, V. B. Hunt, K. W. Gilchrist, M. A. Newton, L. L. Bram, et al. Comparison of bladder cancer outcome in men undergoing hematuria home screening versus those with standard clinical presentations. Urology. 1995;45(3):387–96; discussion 396-7.
6. E. M. Messing, T. B. Young, V. B. Hunt, M. A. Newton, L. L. Bram, A. Vaillancourt, et al. Hematuria home screening: repeat testing results. J Urol. 1995;154(1):57–61.

All material © Copyright 2025 CISNET

Columbia University
Parameter Overview

Reader's Guide
Model Purpose
Model Overview
Assumption Overview
Parameter Overview
Component Overview
Output Overview
Results Overview
Key References

Parameter Overview

Summary

This document provides the parameters used in the Simulation of Cancers of the Urinary Tract (SCOUT) model.

Background

Parameters in the SCOUT model are informed by systematic review, observational data from the literature or nationally representative datasets. Any unknow parameters are calibrated to the targets derived from U.S. observed data.

Parameter Listing Overview

Parameters are listed for each component of the SCOUT model.

Chronic kidney disease

1. Incidence rate of diabetes by age, sex, and race
2. Incidence rate of micro-albuminuria by age, sex, race, and diabetes and hypertension status
3. Initial systolic blood pressure for middle-aged adults
4. Change in systolic blood pressure by age, sex, and presence/absence of CKD
5. Annual rate of decline in GFR by level of GFR, presence of macroalbuminuria, diabetes status, and hypertension status
6. Annual transition probability from micro- to macro-albuminuria by age, sex, diabetes status, and hypertension status
7. Hazard ratios of non-CVD and CVD mortality risk for those with CKD compared to those without CKD stratified by CKD stage
8. CVD mortality rates by age, sex, and race
9. Background mortality rate by age, sex, and race
10. Mortality rates among people with end-stage renal disease by age, sex, race, diabetes status, and hypertension status

Natural history of bladder cancer

1. Prevalence of smoking status in adults age ≥ 40 by sex
2. Proportion of hematuria among non-muscle invasive bladder cancer
3. Probability of developing undetectable low-grade papillary tumor (TaLG) by age, sex, smoking status, and CKD severity
4. Probability of developing undetectable high-grade papillary tumor (TaHG) by age, sex, smoking status, and CKD severity
5. Probability of transitioning from undetectable TaLG to detectable TaLG
6. Probability of transitioning from undetectable TaLG to undetectable TaHG/Tis
7. Probability of transitioning from undetectable TaLG to detectable TaHG/Tis
8. Probability of transitioning from detectable TaLG to detectable TaHG/Tis
9. Probability of transitioning from undetectable TaHG/Tis to detectable TaHG/Tis
10. Probability of transitioning from undetectable TaHG/Tis to undetectable T1 by age
11. Probability of transitioning from undetectable TaHG/Tis to detectable T1 by age
12. Probability of transitioning from detectable TaHG/Tis to detectable T1 by age
13. Probability of transitioning from undetectable T1 to detectable T1
14. Probability of transitioning from undetectable T1 to T2 by age
15. Probability of transitioning from T2 to T3 by age

All material © Copyright 2025 CISNET

16. Probability of transitioning from T3 to T4
17. Probability of transitioning from T4 to metastasis
18. Rate of detection of detectable TaLG
19. Rate of detection of detectable TaHG
20. Rate of detection of detectable T1
21. Proportion of tumor detected in MIBC by T stage, age, and sex
22. Probability of death from metastatic bladder cancer
23. Hazard ratios of risk of bladder cancer for those with severe CKD stage compared to those without CKD
24. Hazard ratios of risk of bladder cancer for former/current smokers compared to non-smokers
25. Hazard ratios of non-CVD and CVD mortality risk for those with CKD compared to those without CKD stratified by CKD stage
26. CVD mortality rates by age, sex, and race
27. Mortality rates among people with end-stage renal disease by age, sex, race, diabetes status, and hypertension status
28. Background mortality rate by age, sex, and race

All material © Copyright 2025 CISNET

Reader's Guide
Model Purpose
Model Overview
Assumption Overview
Parameter Overview
Component Overview
Output Overview
Results Overview
Key References

Component Overview

Summary

This document provides the overview of major components of the Simulation of Cancers of the Urinary Tract (SCOUT) model.

Overview

SCOUT consists of four major components: 1) Chronic kidney disease, 2) Natural history of bladder cancer, 3) Screening (in development), and 4) Treatment and surveillance (in development). All components are designed to run simultaneously by default. However, the chronic kidney disease and the natural history components can be run independently. Consequently, SCOUT can simulate a cohort with CKD progression and without cancer progression. Similarly, a cohort with only cancer progression can be simulated as needed.

Component Listing

Chronic Kidney Disease

The chronic kidney disease component simulates a hypothetical cohort of individuals with the same sex, age, and race who may develop diabetes and/or hypertension over time. These comorbid conditions influence the rate of CKD progression. The CKD component models the progression from no CKD to CKD stages 1 through 5. The severity of CKD stages further informs the risk of bladder cancer, as well as non-CVD and CVD mortality risk. Additionally, SCOUT can simulate a cohort with the same initial CKD stage as well.

Natural history of bladder cancer

By default, the natural history component is developed to run simultaneously with the CKD component, accounting for individual changes in CKD severity in addition to the static smoking status. The smoking status is initialized based on the prevalence of smoking among U.S. adults from age 40, obtained from a national dataset. In the current version of SCOUT, smoking status is assigned at the start of the model and is independent of CKD and, therefore, does not influence CKD progression. The natural history component simulates the progression of bladder cancers, from a disease-free state to the development of bladder tumors, ranging from non-muscle invasive bladder cancers, muscle invasive bladder cancers, and metastasis. All simulated individuals are subject to non-CVD and CVD mortality risks, with varying risks based on CKD severity. Only those with metastasis are subject to death from metastatic disease. Additionally, the natural history of bladder cancer component can be run independent of CKD component as well.

All material © Copyright 2025 CISNET

Reader's Guide
Model Purpose
Model Overview
Assumption Overview
Parameter Overview
Component Overview
Output Overview
Results Overview
Key References

Output Overview

Summary

This document provides an overview of outputs generated from the Simulation of Cancers of the Urinary Tract (SCOUT) model.

Overview

SCOUT tracks individual trajectories of characteristics, risk factors, cancer stage, time to cancer development, time of progression between cancer stages, time of death, and causes of death over a lifetime. Accordingly, SCOUT can generate the outputs for the overall simulated cohort and for sub-cohorts based risk factors and comorbidities.

Output Listing

The model outcomes are generated using a lifetime horizon and can be stratified by smoking status and/or CKD stage. The main outputs include:

- Age- and stage-specific bladder cancer incidence
- Mean age at diagnosis
- Cumulative cancer mortality
- Cumulative all-cause mortality
- Cumulative CVD mortality
- Cumulative end-stage renal disease mortality
- Prevalence of CKD by age group
- Cumulative incidence of end-stage renal disease
- Life expectancy

Additional outputs will be provided when the screening and treatment components are completed.

All material © Copyright 2025 CISNET

[Reader's Guide]
[Model Purpose]
[Model Overview]
[Assumption Overview]
[Parameter Overview]
[Component Overview]
[Output Overview]
[Results Overview]
[Key References]

Results Overview

Summary

This document provides is a summary of the model results from developing the Simulation of Cancers of the Urinary Tract (SCOUT) model and its application.

Overview

SCOUT is a microsimulation following a discrete-time non-homogeneous Markov model. In the current version, we developed and calibrated our model for two components: chronic kidney disease and natural history of bladder cancer. For the chronic kidney disease component, we calibrated the parameter relevant to CKD progression using grid search. Most of our model targets fall within the USRDS reported ranges of prevalence of CKD by age and the cumulative incidence of end-stage renal disease. Lastly, we applied the calibrated parameters relevant to CKD and calibrated the parameters relevant to bladder cancer development and progression. The natural history model was calibrated using incremental mixture approximate Bayesian computation. Our model outcomes match well with the age-specific cancer incidence and stage-specific cancer incidence derived from SEER Cancer registry for white men (Figure 1 and 2).

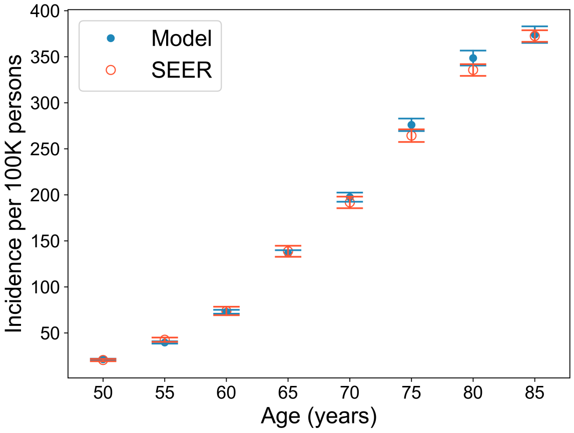

Figure 1. Model calibration for age-specific incidence comparing SCORE against SEER for white men diagnosed in 2010

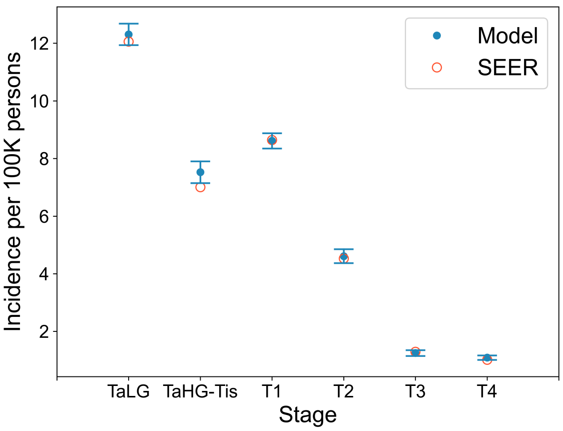

Figure 2. Model calibration for stge-specific incidence comparing SCORE against SEER for white men diagnosed in 2010

Results List

Model results in detail will be updated as a list of publications associated with brief description.

All material © Copyright 2025 CISNET

Columbia University
CKD Component

Reader's Guide
Model Purpose
Model Overview
Assumption Overview
Parameter Overview
Component Overview
Output Overview
Results Overview
Key References

CKD Component

Summary

This document describes the development and disease progression of chronic kidney disease (CKD) of the Simulation of Cancers of the Urinary Tract (SCOUT) model.

Overview

The chronic kidney disease component of SCOUT simulates CKD development and its progression to end-stage renal disease based on a decline in glomerular filtration rate and the development of micro- and macroalbuminuria. Additionally, SCOUT incorporate risk factors associated with CKD by simulating the incidence of diabetes and changes in systolic blood pressure, indicating the presence of hypertension.

Details

SCOUT simulates the development and progression of CKD with seven stages: no CKD, stage 1, stage 2, stage 3a, stage 3b, stage 4, stage 5 (Table 1) using a yearly cycle. Following KDIGO 2012,[1] we categorize CKD stage based on six levels of glomerular filtration rate (GFR) and three albuminuria categories (normal, microalbuminuria, macroalbuminuria). SCOUT incorporated risk factors associated with the risk of CKD including age, sex, race, and two comorbid conditions: hypertension and diabetes (presence/absence). At the beginning of the time horizon, SCOUT simulates a hypothetical cohort of the same sex, age, and race. Each person is assigned GFR level (continuous variable following truncated normal distribution), either having normal or microalbuminuria, presence or absence of diabetes, and level of systolic blood pressure (SBP). Hypertension is defined as SBP > 140 mmHg. At each cycle, SCOUT tracks individual change in diabetes status and SBP level, which further affect rates of progression in CKD stage.

For persons without CKD, they may develop CKD through one of the two pathways: 1) the onset of microalbuminuria, defined as kidney damage or an albumin-to-creatinine ratio (ACR) ≥ 30 mg/g or 2) a decrease in GFR to < 60 mL/min/1.73m^2 (Figure 1). For persons who initially have normal albuminuria but later develop microalbuminuria with GFR ≥ 60 mL/min/1.73m^2, they may transition from a no-CKD state to either CKD stage 1 or stage 2 depending on their GFR levels. The microalbuminuria incidence rate depends on age, sex, race, and the presence/absence of diabetes and hypertension. During their lifetime, persons with microalbuminuria may progress to have macroalbuminuria. For those with a decreased GFR < 60 mL/min/1.73m^2 and without microalbuminuria, the transition occurs directly from a no-CKD state to CKD stage 3a. For those with CKD, they may transition to more severe stages of CKD ranging from stage 1 to stage 5. The rates of transitioning are informed by 1) an annual rate of decline in GFR, 2) the incidence rate of microalbuminuria, and 3) a transition probability from micro- to macroalbuminuria. SCOUT models heterogenous declines in GFR among a simulated cohort with the same risk factors by applying a small adjustment to the rate of decline. To adjust the decline rate, each person receives a coefficient multiplier sampling from the symmetric triangular distribution. Compared to those without macroalbuminuria, those with macroalbuminuria are subject to higher rates of GFR decline. Additionally, those with comorbidity are subject to higher rates of GFR decline, microalbuminuria incidence rates, and probability of transitioning from micro- to macroalbuminuria. From any alive state, a person may die from various causes of death including CVD and non-CVD mortality. CVD and non-CVD mortality rates are higher in persons with more severe CKD stages. For persons with CKD stage 5, they are subject to the mortality rate of end-stage renal disease only. Over the time horizon, SCOUT tracks individual trajectories of age, GFR, SBP, albuminuria category, diabetes status, hypertension status, and CKD stage. See Parameter Overview and Assumption Overview for more details.

Table 1. CKD stage defined by albuminuria and glomerular filtration rate (GFR).

CKD Stage	Albuminuria category	GFR
No CKD	Normal	60+

All material © Copyright 2025 CISNET

CKD Stage	Albuminuria category	GFR
1	Microalbuminuria or Macroalbuminuria	90+
2	Microalbuminuria or Macroalbuminuria	60-89
3a	Any	45-60
3b	Any	30-45
4	Any	15-30
5	Any	<15

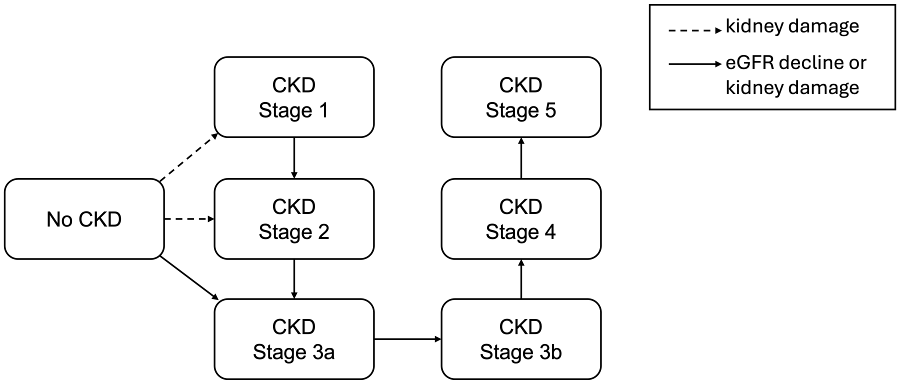

Figure 1. Chronic kidney disease development and progression.

To align with the U.S. observed data, we calibrated the CKD component for men to targets derived from the USRDS 2023 Annual Report.[2] The targets include the prevalence of CKD by age, the cumulative incidence of end-stage renal disease, the prevalence of CKD by GFR and age, and the prevalence of micro- and macroalbuminuria by GFR and age. We prioritized matching our model outcomes to the prevalence of CKD by age and the cumulative incidence of end-stage renal disease. The calibrated parameters include diabetes incidence rates, microalbuminuria incidence rates, and the transition probability from microalbuminuria to macroalbuminuria. All model parameters were informed by systematic review, existing models, and NHANES. We used grid search and imposed a range constraint such that the calibrated parameters are varied within allowable ranges of ±30% of the initial parameter values.

Relevant Assumptions

The main assumption governing the chronic kidney disease component is that the risk of developing CKD and progressing to more severe CKD stages depends on the presence of risk factors including age, sex, race, diabetes, and hypertension. Additionally, worsening kidney functions such as low GFR and/or macroalbuminuria leads to an acceleration of disease worsening

Relevant Parameters

Parameters associated with the Chronic kidney disease component are listed in the Parameter Overview.

Dependent Outputs

- Prevalence of CKD
- Prevalence of micro- or macroalbuminuria among persons with CKD
- Incidence of end-stage renal disease
- End-stage renal disease mortality

References

1. A. Levin, P. E. Stevens. Summary of KDIGO 2012 CKD Guideline: behind the scenes, need for guidance, and a framework for moving forward. Kidney Int. 2014;85(1):49–61.

2. United States Renal Data System. 2023 USRDS Annual Data Report: Epidemiology of kidney disease in the United States [Internet]. 2023. Available from: https://adr.usrds.org/2023

SCOUT (Columbia University)

BLADDER

Columbia University
Natural History
Component

Reader's Guide
Model Purpose
Model Overview
Assumption Overview
Parameter Overview
Component Overview
Output Overview
Results Overview
Key References

Natural History Component

Summary

This document describes the natural history model including the progression from a cancer-free state to death from metastatic disease.

Overview

The natural history model of SCOUT simulates the development of undetectable low-grade or high-grade papillary tumors. These tumors may progress by increasing in grades, transition from undetectable to detectable or from detectable to detected state, and advancing in T stages, and eventually reaching metastatic disease. The disease progression follows nonhomogeneous Markov model with a monthly cycle and a lifetime horizon. SCOUT incorporates risk factors associated with risk of bladder cancer including sex, age, race, risk behavior of smoking, and chronic kidney disease (CKD) severity.

Details

SCOUT simulates the development and progression of bladder cancers with four main health states: disease-free, non-muscle invasive bladder cancers or NMIBC (Ta, Tis, T1), muscle invasive bladder cancers or MIBC (T2, T3, T4 excluding metastatic disease), and metastasis, using a monthly cycle. The model incorporates risk factors associated with the risk of bladder cancers including age, sex, race, smoking status, and CKD stage. At the beginning of the time horizon, SCOUT simulates a hypothetical cohort of the same sex, age, and race. Each person is assigned one of the three smoking statuses (Never, Former, Current), which remains static in the current version of SCOUT, and a CKD stage based on their glomerular filtration rate and albuminuria category (See Chronic Kidney Disease component for more details). SCOUT tracks individual change in CKD stage, which in turn influence the rates of bladder tumor development and the mortality rates. The natural history of bladder cancer component is initiated when the cohort reaches age 40, given the low prevalence of bladder cancer before this age.

SCOUT explicitly models three states—undetectable (tumor presence without symptoms), detectable (microscopic or macroscopic hematuria), and detected—for each of TaLG and TaHG-Tis combined in NMIBC. For MIBC, any T stages are assumed to be symptomatic and detectable. However, only a proportion of these tumors are detected according to the observed cancer incidence from SEER cancer registry. Any detected cancers are assumed to be treated according the NCCN guidelines. The treatment module is currently in development. The development and progression of bladder cancer in SCOUT follows a Markov model (Figure 1), where the transition probability at cycle t depends only on the health state at cycle t–1. To apply a Markov model, SCOUT follows the steps below:

1. A transition matrix is first established based on all transition probability inputs listed in the Parameter Overview.
2. The probability of remaining in the same health state is calculated as 1 minus the sum of probabilities of transitioning to other states.
3. At each cycle, the next health state is determined by sampling from a categorical distribution, using the probability distribution derived from the transition matrix.
4. The transition matrices are updated monthly to account for individual changes in various risk factors.

All material © Copyright 2025 CISNET

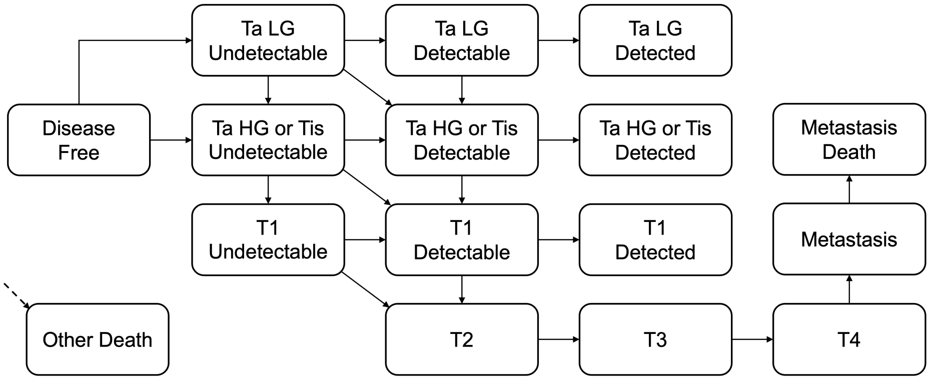

Figure 1. State-transition diagram depicting the progression of bladder cancer from a disease-free state to T stages among non-muscle and muscle invasive bladder cancers, metastasis, and death from metastasis or other death.

Persons who are initially disease-free may develop bladder tumor through one of the two pathways: 1) developing low-grade papillary bladder tumor or TaLG or 2) developing high-grade papillary bladder tumor or TaHG. For simplicity, TaHG and Tis are grouped into a combined state. At any cycle, persons with a bladder tumor may transition to more severe states including transitioning from undetectable to detectable state, from detectable to detected state, from low to high grade, increase in T stage, and may eventually reach metastasis. At any cycle, persons may die from one of the competing causes of death including CVD, non-CVD, or metastasis death (applied only to persons with metastasis). SCOUT tracks individual trajectories of age, smoking status, CKD stage, health state at cycle t and cycle t–1, death status, and causes of death.

To align with the observational bladder cancer incidence from SEER, we calibrated SCOUT for white men using incremental mixture approximate Bayesian computation algorithm [1] to the age- and stage-specific cancer incidence. The calibrated parameters include the probability of developing bladder tumor, the probability of transitioning from undetectable to detectable state, the probability of transitioning from detectable to detected state, the probability of transitioning from less severe to more severe T stages, and the proportion of cancer detected in MIBC.

Relevant Assumptions

The main assumption governing the natural history model is that all cancers must arise from either low-grade or high-grade papillary tumors. Among NMIBC, all tumors go from an undetectable state to a detectable state and reach a detected state if they are clinically diagnosed. MIBC is assumed to be symptomatic and detectable with a proportion assumed to be detected according to SEER observed incidence. Additionally, the risk for bladder cancer increases for those with older age, having severe CKD stages, or are smokers.

Relevant Parameters

Parameters associated with the Natural history component are listed in the Parameter Overview.

Dependent Outputs

- Age- and stage-specific cancer incidence
- Cancer stage at diagnosis
- Cancer mortality

References

1. Carolyn M. Rutter, Jonathan Ozik, Maria DeYoreo, Nicholson Collier. Microsimulation model calibration using incremental mixture approximate Bayesian computation. The Annals of Applied

Statistics. 2019;13(4):2189–2212, 24.

All material © Copyright 2025 CISNET

Columbia University
Key References

[Reader's Guide](#)
[Model Purpose](#)
[Model Overview](#)
[Assumption Overview](#)
[Parameter Overview](#)
[Component Overview](#)
[Output Overview](#)
[Results Overview](#)
[Key References](#)

Key References

M. Afkarian, M. C. Sachs, B. Kestenbaum, I. B. Hirsch, K. R. Tuttle, J. Hinnmelfarb, et al. Kidney Disease and Increased Mortality Risk in Type 2 Diabetes. Journal of the American Society of Nephrology. 2013;24(2):302–308.

L. E. Boulware, B. G. Jaar, M. E. Tarver-Carr, F. L. Brancati, N. R. Powe. Screening for proteinuria in US adults: a cost-effectiveness analysis. JAMA. 2003;290(23):3101–14.

Emily R Brooks, Mutita Siriruchatanon, Vinay Prabhu, David M Charytan, William C Huang, Yu Chen, et al. Chronic kidney disease and risk of kidney or urothelial malignancy: systematic review and meta-analysis. Nephrology Dialysis Transplantation. 2023;

H. Calkins, Y. Shyr, H. Frumin, A. Schork, F. Morady. The value of the clinical history in the differentiation of syncope due to ventricular tachycardia, atrioventricular block, and neurocardiogenic syncope. Am J Med. 1995;98(4):365–73.

A. Christensson, C. Savage, D. D. Sjoberg, A. M. Cronin, M. F. O'Brien, W. Lowrance, et al. Association of cancer with moderately impaired renal function at baseline in a large, representative, population-based cohort followed for up to 30 years. Int J Cancer. 2013;133(6):1452–8.

N. D. Freedman, D. T. Silverman, A. R. Hollenbeck, A. Schatzkin, C. C. Abnet. Association between smoking and risk of bladder cancer among men and women. JAMA. 2011;306(7):737–45.

A. X. Garg, B. A. Kiberd, W. F. Clark, R. B. Haynes, C. M. Clase. Albuminuria and renal insufficiency prevalence guides population screening: results from the NHANES III. Kidney Int. 2002;61(6):2165–75.

A. S. Go, G. M. Chertow, D. Fan, C. E. McCulloch, C. Y. Hsu. Chronic kidney disease and the risks of death, cardiovascular events, and hospitalization. N Engl J Med. 2004;351(13):1296–305.

T. J. Hoerger, J. S. Wittenborn, J. E. Segel, N. R. Burrows, K. Imai, P. Eggers, et al. A health policy model of CKD: 1. Model construction, assumptions, and validation of health consequences. Am J Kidney Dis. 2010;55(3):452–62.

A. Levin, P. E. Stevens. Summary of KDIGO 2012 CKD Guideline: behind the scenes, need for guidance, and a framework for moving forward. Kidney Int. 2014;85(1):49–61.

Y. Lotan, K. Elias, R. S. Svatek, A. Bagrodia, G. Nuss, B. Moran, et al. Bladder cancer screening in a high risk asymptomatic population using a point of care urine based protein tumor marker. J Urol. 2009;182(1):52–7; discussion 58.

W. T. Lowrance, J. Ordonez, N. Udaltsova, P. Russo, A. S. Go. CKD and the risk of incident cancer. J Am Soc Nephrol. 2014;25(10):2327–34.

E. M. Messing, T. B. Young, V. B. Hunt, J. M. Wehbie, P. Rust. Urinary tract cancers found by homescreening with hematuria dipsticks in healthy men over 50 years of age. Cancer. 1989;64(11):2361–7.

E. M. Messing, T. B. Young, V. B. Hunt, E. B. Roecker, A. M. Vaillancourt, W. J. Hisgen, et al. Home screening for hematuria: results of a multiclinic study. J Urol. 1992;148(2 Pt 1):289–92.

E. M. Messing, T. B. Young, V. B. Hunt, K. W. Gilchrist, M. A. Newton, L. L. Bram, et al. Comparison of bladder cancer outcome in men undergoing hematuria home screening versus those with standard clinical presentations. Urology. 1995;45(3):387–96; discussion 396-7.

E. M. Messing, T. B. Young, V. B. Hunt, M. A. Newton, L. L. Bram, A. Vaillancourt, et al. Hematuria home screening: repeat testing results. J Urol. 1995;154(1):57–61.

S. D. Navaneethan, J. D. Schold, S. Arrigain, S. E. Jolly, Jr. Nally J. V. Cause-Specific Deaths in Non-Dialysis-Dependent CKD. J Am Soc Nephrol. 2015;26(10):2512–20.

Carolyn M. Rutter, Jonathan Ozik, Maria DeYoreo, Nicholson Collier. Microsimulation model calibration using incremental mixture approximate Bayesian computation. The Annals of Applied Statistics. 2019;13(4):2189–2212, 24.

R. L. Siegel, K. D. Miller, N. S. Wagle, A. Jemal. Cancer statistics, 2023. CA Cancer Journal for Clinicians. 2023;73(1):17–48.

All material © Copyright 2025 CISNET

United States Renal Data System. 2023 USRDS Annual Data Report: Epidemiology of kidney disease in the United States [Internet]. 2023. Available from: https://adr.usrds.org/2023

B. O. Yarnoff, T. J. Hoerger, S. S. Shrestha, S. K. Simpson, N. R. Burrows, A. H. Anderson, et al. Modeling the impact of obesity on the lifetime risk of chronic kidney disease in the United States using updated estimates of GFR progression from the CRIC study. PLoS One. 2018;13(10):e0205530.
