# Supplementary material for: Bladder Cancer Burden in the USA: Population Scenarios for 2040
Source: Eur Urol Open Sci. 2025 Dec 4;83:99–108. doi: 10.1016/j.euros.2025.11.013 (PMC12721285; doi:10.1016/j.euros.2025.11.013)
Supplement: Supplementary Data 4 [file mmc4.docx]

# Supplement table of contents

[Supplement table of contents 1](#_Toc194581199)

[Table: Model grid 2](#_Toc194581200)

[Figure: 2010 stage specific incidence in US white men with each model 5](#_Toc194581201)

[Cancer Incidence and Surveillance Modeling Network (CISNET) Bladder Cancer Site: Overview 7](#_Toc194581202)

[COBRAS Model Profile 10](#_Toc194581203)

[Kystis Model Profile 10](#_Toc194581204)

[SCOUT Model Profile 10](#_Toc194581205)

[CISNET Bladder Cancer Modeling Investigators list 10](#_Toc194581206)

# Table: Model grid

| **Property** | **COBRAS** | **Kystis** | **SCOUT** |
| --- | --- | --- | --- |
| **Model characteristics** |  |  |  |
| Model type | Continuous time DES | Continuous time DES | Discrete Time State-Transition |
| Population model | Yes | Yes | Yes |
| Model individuals (microsimulation) | Yes | Yes | Yes |
| Model of organ | No | Yes | No |
| **Lesion risk** |  |  |  |
| Mechanism | Random-Intercept Zero-Inflated Poisson Model | Non-Homogenous Poisson Point Process | Non-Homogeneous Markov Model |
| Demographic risk factors | Age, sex, race, birth-year | Age, sex, race, birth-year | Age, sex, race, birth-year |
| Modifiable risk factors: |  |  |  |
| Smoking | Yes | Yes | Yes |
| Occupational/ environmental toxins | Yes | Yes | No |
| Genetic risk factors | No | Lynch Syndrome | No |
| Comorbidities | No | No | Chronic Kidney Disease Stage, Diabetes Mellitus, Hypertension |
| Varies randomly across individuals | Yes | Yes | Yes |
| Multiple lesions possible | Yes | Yes | Yes |
| **Lesion attributes** |  |  |  |
| Transitional cell carcinoma | PUNLMP, Ta, T1-4, Tis | Lesions of low invasive potential (Ta, T1, T2+, Tis) | Ta, T1-4, Tis |
| Grade | Low/High (Ta only) | Low/High (Ta and T1) | Low/High (Ta only) |
| Non-transitional cell carcinoma | Yes | Yes | No |
| Morphology/configuration | Not explicitly modeled | Flat, Sessile, Pedunculated | No |
| Size | Lesion diameter and volume | Cell number and surface area on bladder | No |
| Location within bladder | No | Mapped to 678 unit areas/tiles on a bladder | No |
| **Lesion growth model** |  |  |  |
| Mechanism | Gompertzian | Generalized logistic (Verhulst) | Not applicable |
| Size modeled as continuous | Yes | Yes | Not applicable |
| Vary randomly across individuals | Yes | Yes | Not applicable |
| Vary randomly across lesions | Yes | Yes | Not applicable |
| **Transitions among lesions** |  |  |  |
| Initial lesion | Ta-LG, Ta-HG, Tis | Ta-LG, Ta-HG, Tis, T1-HG (for non urothelial neoplams) | Ta-LG, Ta-HG/Tis |
| Allowable transitions | Ta-LG → Ta-HG → T1 → T2 → T3 → Metastasis | For both LG and HG lesions, Ta → T1 → T2/T3 → Metastasis, and similar for Tis | Ta-LG → Ta-HG/Tis → T1 → T2 → T3 → non-Metastasis T4 → Metastasis |
| Mechanism | Stochastic function of lesion size | Stochastic function of lesion size | Age-dependent Stochastic matrix |
| Vary systematically by | Lesion stage and grade | Lesion stage and grade | Lesion stage and grade |
| Vary randomly across individuals | Yes | Yes | Yes |
| Vary randomly across same-size lesions within individuals | Yes | Yes | Yes |
| Nodal metastases explicitly modeled | Yes | No | No |
| Metastasis process explicitly modeled | Yes | Yes | Yes |
| **Mortality** |  |  |  |
| Cancer deaths | Can only occur if metastasis occurs | Can occur if MIBC (T2/T3) or metastasis (T4) occur | Can only occur if metastasis occurs |
| Other deaths | Same as general population | Same as general population | Risk of all-cause and cardiovascular deaths stratified by CKD stages |
| **Transitions to Clinical Disease** |  |  |  |
| Mechanism | Time-to-event distribution | Stochastic function of lesion size | Age-dependent Stochastic matrix |
| Symptoms modeled explicitly | No (only diagnosis modeled) | Microscopic or macroscopic hematuria, voiding symptoms | Microscopic or macroscopic hematuria (detectable state) |
| Vary systematically by | Sex, race, lesion T-stage | Sex, race, lesion T-stage | Sex, race, lesion T-stage, lesion grade |
| Vary randomly across individuals | Yes | Yes | Yes |
| Vary randomly across same-size lesions within individuals | Yes | Yes | Yes |
| **Post-Tx Recurrence & Progression** |  |  |  |
| Recurrence mechanisms: |  |  |  |
| Incomplete TUR/regrowth | No | Yes | No |
| New lesions | Yes | Yes | Yes |
| Missed lesions | Yes | Yes | Yes |
| Vary systematically by | Lesion histology, grade, T-stage, lesion size, concomitant Tis, treatment | Lesion histology, grade, T-stage, lesion size, treatment | Lesion grade, T-stage, treatment |
| Progression mechanisms: |  |  |  |
| Transitions among preclinical new and missed lesions | Yes | Yes | Yes |
| Transitions among regrown lesions | No | Yes | No |
| Understaged lesions | Yes | Yes | Yes |
| Vary systematically by | Lesion histology, grade, T-stage, lesion size, concomitant CIS, treatment | Lesion histology, grade, T-stage, lesion size, treatment | Lesion grade, T-stage, treatment |
| **Development** |  |  |  |
| Software | R | R | Python |
| Uncertainty | Stochastic | Stochastic | Stochastic |
| Calibration | Bayesian | Bayesian | Bayesian |

# Figure: 2010 stage specific incidence in US white men with each model

Shown is the age stage specific incidence for US white men in 2010 with the three models versus the SEER data. The models simulate the age composition of the US population of white men in 2010 as described in the legend of **Figure 1** in the main text. The stage specific incidence is calculated as the number of new bladder cancer cases diagnosed at each stage in 2010 irrespective of age at diagnosis divided by the total number of person-years observed in 2010 times ${10}^{5}$. Panels **a** (COBRAS), **b** (Kystis), and **c** (SCOUT) show results with the most granular stage categories in the corresponding model.

| a   |
| --- |
| b   |
| c   |

# Cancer Incidence and Surveillance Modeling Network (CISNET) Bladder Cancer Site: Overview

**Background**

Bladder cancer is the sixth most common cancer in the United States, with an estimated 83,000 new cases expected in 2024.^1^ While most cases are diagnosed at an early stage, the disease remains among the most expensive to manage due to its high recurrence rate.^2,3^ Despite reductions in major risk factors such as smoking, progress in lowering bladder cancer incidence and mortality has been limited.^4,5^ Currently, routine screening for bladder cancer is not recommended due to insufficient evidence.^6^ Available screening tools – such as urine cytology and biomarker tests – lack the sensitivity and specificity needed to reliably detect early-stage disease.^7^ The gold standard diagnostic tool, cystoscopy, is invasive and costly, making it impractical for widespread population use.^8^ Post-diagnosis surveillance also presents challenges, with questions remaining about how best to tailor the frequency and combination of follow-up tests based on individual risk.^9,10^

To address these gaps, the CISNET Bladder Cancer Incubator was established in 2021. Its goal is to identify effective and cost-efficient strategies for bladder cancer screening, surveillance, and treatment through advanced population modeling, ultimately supporting future guideline development. The Incubator began with two modeling groups:

- **COBRAS** (Cancer of the Bladder R-based Analytic Simulator), with investigators from the University of Ottawa, Stanford University, and the University of Pittsburgh.
- **Kystis**, with investigators from Brown University and Tufts University.

In 2022, the **SCOUT** (Simulation of Cancers of the Urinary Tract) group from Columbia University joined as an affiliate member. All three modeling groups maintain methodological independence and are supported by the Coordinating Center at Brown University.

**Model Structure**

The three CISNET Bladder Cancer models simulate individual life histories to estimate population-level trends in bladder cancer. COBRAS and Kystis operate in continuous time, while SCOUT uses monthly cycles. All models simulate U.S. birth cohorts beginning in 1900, aging them forward with realistic patterns of bladder cancer incidence, progression, and death, but without accounting for migration. The simulations incorporate age-specific bladder cancer hazards, and individuals may develop one or more tumors that can progress from non-invasive to invasive and metastatic stages. Diagnosis is determined by a combination of tumor characteristics, symptoms (e.g., bleeding or voiding), and demographic factors like race and sex to capture observed differences in stage and age at diagnosis.

All models incorporate smoking exposure. COBRAS and Kystis use historically accurate smoking patterns generated by CISNET’s Smoking History Generator,^11^ while SCOUT applies fixed, age-specific smoking rates. All models simulate key events such as tumor onset, progression, metastasis, diagnosis, and cause-specific death, informed by U.S. mortality data.^12^ In addition to bladder cancer natural history, SCOUT simulates chronic kidney disease progression by modeling declines in kidney function and related risk factors including diabetes and hypertension.

Model inputs and calibration targets included the Surveillance, Epidemiology, and End Results (SEER) data on bladder cancer incidence, stage, age distribution, mortality, and relative survival;^13^ individual and published data from nine EORTC trials on early-stage bladder cancer to inform recurrence and progression;^14^ cohort-specific U.S. mortality projections from the CDC, stratified by sex and race;^12^ population estimates and forecasts from the U.S. Census;^15,16^ and additional data sourced from a related project that develops and maintains evidence maps of the bladder cancer literature. Calibration was performed using distinct optimization strategies, and all models underwent extensive verification to ensure biological plausibility, logical consistency, and dimensional correctness. Model outcomes are probabilistic, reflecting randomness in event timing and uncertainty in parameter values, with final results drawn from the top 1,000 best-fitting parameter sets.

**Public Health Impact**

Understanding the natural history of bladder cancer is crucial for evaluating prevention and control strategies, yet many events, such as the duration between when a cancer is first screen-detectable and when it presents clinically, are not directly observable in routine data. The CISNET Bladder Cancer models address this challenge by using available epidemiological and clinical data to infer unobserved events. These models provide a valuable tool for decision-makers to assess the long-term impact of health policies and newly emergent technologies on bladder cancer incidence, prevalence, and mortality in the U.S. – for example, evaluating how environmental carcinogen control, targeted early detection, intensified surveillance for high-risk groups, or novel treatments for advanced cancers could influence trends in bladder cancer outcomes.

**References**

1. Siegel RL, Giaquinto AN, Jemal A. Cancer statistics, 2024. *CA Cancer J Clin*. Jan-Feb 2024;74(1):12-49. doi:10.3322/caac.21820

2. Burger M, Catto JW, Dalbagni G, et al. Epidemiology and risk factors of urothelial bladder cancer. *Eur Urol*. Feb 2013;63(2):234-41. doi:10.1016/j.eururo.2012.07.033

3. Clark O, Sarmento T, Eccleston A, et al. Economic Impact of Bladder Cancer in the USA. *Pharmacoecon Open*. Nov 2024;8(6):837-845. doi:10.1007/s41669-024-00512-8

4. Leas EC, Trinidad DR, Pierce JP, McMenamin SB, Messer K. Trends in cigarette consumption across the United States, with projections to 2035. *PLoS One*. 2023;18(3):e0282893. doi:10.1371/journal.pone.0282893

5. Su X, Tao Y, Chen F, Han X, Xue L. Trends in the global, regional, and national burden of bladder cancer from 1990 to 2021: an observational study from the global burden of disease study 2021. *Sci Rep*. Mar 5 2025;15(1):7655. doi:10.1038/s41598-025-92033-5

6. Moyer VA, Force USPST. Screening for bladder cancer: U.S. Preventive Services Task Force recommendation statement. *Ann Intern Med*. Aug 16 2011;155(4):246-51. doi:10.7326/0003-4819-155-4-201108160-00008

7. Chou R, Dana T. Screening adults for bladder cancer: a review of the evidence for the U.S. preventive services task force. *Ann Intern Med*. Oct 5 2010;153(7):461-8. doi:10.7326/0003-4819-153-7-201010050-00009

8. Devlies W, de Jong JJ, Hofmann F, et al. The Diagnostic Accuracy of Cystoscopy for Detecting Bladder Cancer in Adults Presenting with Haematuria: A Systematic Review from the European Association of Urology Guidelines Office. *Eur Urol Focus*. Jan 2024;10(1):115-122. doi:10.1016/j.euf.2023.08.002

9. Parrao D, Lizana N, Saavedra C, et al. Active Surveillance in Non-Muscle Invasive Bladder Cancer, the Potential Role of Biomarkers: A Systematic Review. *Curr Oncol*. Apr 12 2024;31(4):2201-2220. doi:10.3390/curroncol31040163

10. Su ZT, Florissi IS, Mahon KM, et al. Varying the intensity of cystoscopic surveillance for high-risk non-muscle-invasive bladder cancer. *BJU Int*. Jan 2025;135(1):148-155. doi:10.1111/bju.16521

11. Jeon J, Holford TR, Levy DT, et al. Smoking and Lung Cancer Mortality in the United States From 2015 to 2065: A Comparative Modeling Approach. *Ann Intern Med*. Nov 20 2018;169(10):684-693. doi:10.7326/M18-1250

12. Prevention CfDCa. National Vital Statistics System – Mortality Data. Accessed March 31, 2025. <https://www.cdc.gov/nchs/nvss/deaths.htm>.

13. Institute NC. Surveillance, Epidemiology, and End Results (SEER) Program. SEER*Stat Database. Accessed March 31, 2025. <https://seer.cancer.gov/data/>

14. Sylvester RJ, van der Meijden AP, Oosterlinck W, et al. Predicting recurrence and progression in individual patients with stage Ta T1 bladder cancer using EORTC risk tables: a combined analysis of 2596 patients from seven EORTC trials. *Eur Urol*. Mar 2006;49(3):466-5; discussion 475-7. doi:10.1016/j.eururo.2005.12.031

15. Bureau USC. Population and Housing Unit Estimates. Accessed March 31, 2025. <https://www.census.gov/popest>

16. Bureau USC. Population Projections. Accessed March 31, 2025. <https://www.census.gov/popest>

#

# COBRAS Model Profile

Separate pdf in this supplement (25 pages)

# Kystis Model Profile

Separate pdf in this supplement (29 pages)

# SCOUT Model Profile

Separate pdf in this supplement (22 pages)

# CISNET Bladder Cancer Modeling Investigators list

| Bladder Site member (leads) | Institution |
| --- | --- |
| Fernando Alarid-Escudero | Stanford University |
| Jamie Cheng | Brown University |
| Tanvi Chiddarwar | University of Minnesota |
| Stavroula Chrysanthopoulou | Brown University |
| Eric Feuer | NCI |
| David Garibay-Treviño | Health Research Consortium (CISIDAT), Mexico |
| Andrew Huang | Brown University |
| Bruce Jacobs | University of Pittsburgh |
| **Hawre Jalal (lead, COBRAS)** | University of Ottawa, Canada |
| **Stella Kang (lead, SCOUT)** | NYU Grossman School of Medicine |
| Praveen Kumar | University of Pittsburgh |
| Karen Kuntz | University of Minnesota |
| Annie Liang | Brown University |
| Paul Mathew | Tufts Medical Center |
| Evangelia Ntzani | Brown University and University of Ioannina, Greece |
| Howard Parnes | NCI |
| Jonah Popp | Brown University |
| Yuliia Sereda | Brown University |
| Mutita Siriruchatanon | NYU Langone Health |
| Natasha Stout | NCI |
| Chen Suen | NCI |
| Nikolaos Trichakis | MIT |
| **Thomas Trikalinos (lead Kystis)** | Brown University |
| John Wong | Tufts Medical Center |
